# Supplementary material for: Time-to-recovery from severe pneumonia and its predictors among children 2–59 months of age admitted to the pediatric ward of Jimma University Medical Center, Southwest Ethiopia, 2023: A retrospective cohort study
Source: PLoS One. 2025 Apr 15;20(4):e0316839. doi: 10.1371/journal.pone.0316839 (PMC11999157; doi:10.1371/journal.pone.0316839)
Supplement: Attachment — Submitted filename: PONE-D-24-04566_R1.pdf [file pone.0316839.s003.pdf]

PLOS ONE

Time-to-Recovery from Severe Pneumonia and Its Predictors Among Children 2-59 Months of Age Admitted to Pediatric Ward of Jimma University Medical Center, Southwest Ethiopia, 2023: A Retrospective Cohort Study.

--Manuscript Draft--

**Manuscript Number:**	PONE-D-24-04566R1
**Article Type:**	Research Article
**Full Title:**	Time-to-Recovery from Severe Pneumonia and Its Predictors Among Children 2-59 Months of Age Admitted to Pediatric Ward of Jimma University Medical Center, Southwest Ethiopia, 2023: A Retrospective Cohort Study.
**Short Title:**	Time-to-Recovery from Severe Pneumonia and Its Predictors Among Children 2–59 Months of Age
**Corresponding Author:**	Getu Girma Bekele, masters<br>Jimma University Medical Center<br>Jimma, Oromia ETHIOPIA
**Keywords:**	Time to Recovery, Severe Pneumonia, predictors, Children 2-59 Months, Jimma
**Abstract:**	Background: Pneumonia is an inflammation of the lung parenchyma. The World Health Organization estimates that 156 million cases of pneumonia, including up to 20 million cases severe enough to require hospitalization, and 1.2 million deaths each year among under-five children. Ethiopia is ranked sixth among top fifteen countries in terms of pneumonia related morbidity and mortality. The study aimed to assess the time to recovery from severe pneumonia and its predictors among children aged 2-59 months admitted to the pediatric ward of Jimma University Medical Center; Southwest, Ethiopia, 2023.<br>Methods: A facility-based retrospective cohort study was carried out among 426 children aged between 2 and 59 months. Five years of medical records, from 2018 – 2022, were reviewed. A simple random sampling technique was used. Data entry was done using epidata version 4.6 and exported to and analyzed by STATA version 15. Kaplan Meier's plot was used to compare the survival probability of different groups and the multivariable Cox regression model was used to investigate the independent effect of covariates on recovery time.<br>Result: The median recovery time was 4 days (IQR: 3, 7). Incidence rate of recovery was 15.78 per 100-person day (95% CI 14.2 – 17.5). The presence of comorbidity (AHR; 0.7, 95% CI (0.54 – 0.91)), being treated with ceftazidime and vancomycin (AHR; 0.29, 95% CI (0.14 – 0.60)), antibiotic change (AHR; 0.74, 95% CI (0.58 – 0.95)) and late presentation to the Hospital (AHR; 0.58, 95% CI (0.43 -0.78)) were statistically significant predictors that prolong recovery time.<br>Conclusion: The median recovery time was longer than other similar studies. Therefore, due attention should be given concerning the identified predictors of the recovery time.
**Order of Authors:**	Getu Girma Bekele, BSc, MSc
	Masrie Getnet, Bsc, Msc
	Dawit Regassa, BSc, MSc
	Tola Getachew Bekele, BSc, Msc
**Response to Reviewers:**	PLOS ONE| Editorial Office - Collaborative Peer Review Team Comments to Author:<br>Independent Review Report, Reviewer 1<br>Abstract: inadequately clear<br>Response: We appreciate the comments and suggestions; the recommendation you raised were briefly written but we deleted them for the sake of word limit and now we have made corrections in the specified section as per the comments.<br>Background: needs further enrichment for relevance and contextual factors<br>Response: we have made corrections in the specified section as per the comments.<br>Methods: should be presented in explicit and comprehensive manner including ethical considerations.

Powered by Editorial Manager® and ProduXion Manager® from Aries Systems Corporation

	Response: Thank you for your constructive comments and we made appropriate corrections in the specified section as per the comments<br>Operational definition: Good if all are referenced with WHO standards or any other = optional<br>Response: Thank you. We made corrections in the section<br>Survival time: What is its difference from that of time-to-recovery?<br>Response: Thank you for the comments, survival time is the general term that indicates the time it takes for the certain event(out come of interest) to occur and our event in this particular study was recovery. Indeed there is no such difference between them and we have deleted survival time<br>Do you have graph?<br>Response: Thank you for the comments, yes we do have graphs like Kaplan Meier survival curve and Cox-snell.<br>Why? What is unique about residence?<br>Response: Thank you for the comments, in this particular study event(recovery) was higher than censoring but we made a mistake about residence during typing (simply typing error) and so we made corrections in the section.<br>Results and discussion: lacks brief and comprehensive presentation with appropriate references and justification<br>Response: Thank you. We made corrections in the section.<br>Table 2 Revisit your percentages<br>Response: Thank you. We made corrections in the section.<br>Vaccination status: Repeated<br>Response: Thank you. We mistakenly repeated and we deleted repetition.<br>After performing multivariable Cox regression, adjusted hazard ratios with a 95% confidence interval (CI) and a p-value of less than or equal to 0.05. Thus, variables like the presence of comorbidities, duration of time before seeking health care, antibiotic first administered and antibiotics change were statistically significant predictors of time to recovery from severe pneumonia in this study. Seems complete?<br>Response: Thank you. We made corrections in the section as After performing multivariable Cox regression, adjusted hazard ratios with a 95% confidence interval (CI) and a p-value of less than or equal to 0.05 were used to declare statistical significance. Thus, Variables such as presence of co-morbidities, duration of time before seeking health care, antibiotic first administered and antibiotics change were statistically significant predictors of time to recovery from severe pneumonia in this study.<br>Table 3: Age did not contribute. Why?<br>Response: Thank you. After controlling the potential confounding factors, there is no siginficant difference in the age between 2-11 months and 12-35 months, indicating that the crude effect was likely not due to the factors being studied, but rather the other confounding factors.<br>In discussion part:<br>Have you read any single article that tells you this information?<br>Response: Thank you for the comments and we cited the references as per the comments in our manuscript.<br>Rewrite. It is a long sentence to capture your idea. So, split into two and rewrite it again<br>Rewrite in a clear way<br>Response: Thank you. We made corrections in the section.
**Additional Information:**	
**Question**	**Response**
**Financial Disclosure**<br><br>Enter a financial disclosure statement that describes the sources of funding for the work included in this submission. Review the submission guidelines for detailed requirements. View published research articles from *PLOS ONE* for specific examples.	The author(s) received no specific funding for this work.

This statement is required for submission and **will appear in the published article** if the submission is accepted. Please make sure it is accurate.<br><br>**Funded studies**<br>Enter a statement with the following details:<br>• Initials of the authors who received each award<br>• Grant numbers awarded to each author<br>• The full name of each funder<br>• URL of each funder website<br>• Did the sponsors or funders play any role in the study design, data collection and analysis, decision to publish, or preparation of the manuscript?<br><br>Did you receive funding for this work?	
**Competing Interests**<br><br>Use the instructions below to enter a competing interest statement for this submission. On behalf of all authors, disclose any competing interests that could be perceived to bias this work—acknowledging all financial support and any other relevant financial or non-financial competing interests.<br><br>This statement is required for submission and **will appear in the published article** if the submission is accepted. Please make sure it is accurate and that any funding sources listed in your Funding Information later in the submission form are also declared in your Financial Disclosure statement.<br><br>View published research articles from *PLOS ONE* for specific examples.	The authors have declared that no competing interests exist.

**NO authors have competing interests**<br><br>Enter: *The authors have declared that no competing interests exist.*<br><br>**Authors with competing interests**<br><br>Enter competing interest details beginning with this statement:<br><br>*I have read the journal's policy and the authors of this manuscript have the following competing interests: [insert competing interests here]*<br><br>* typeset	
**Ethics Statement**<br><br>Enter an ethics statement for this submission. This statement is required if the study involved:<br><br>• Human participants<br>• Human specimens or tissue<br>• Vertebrate animals or cephalopods<br>• Vertebrate embryos or tissues<br>• Field research<br><br>Write "N/A" if the submission does not require an ethics statement.<br><br>General guidance is provided below. Consult the submission guidelines for detailed instructions. **Make sure that all information entered here is included in the Methods section of the manuscript.**	Before data collection, this research protocol has been submitted to the Institutional Review Board (IRB) of Jimma University and meets the ethical and scientific standards outlined in national and international guidelines. Jimma University, Institute of Health (Reference No JUIH/IRB/361/23) approved the consent wavier. The letter of permission was written to Jimma University Medical Center managers. Letter to access the patient's chart was obtained from the chief executive officer (CEO) of Jimma University Medical Center and the health management information system focal person verbally consented to use the patients' medical chart for data collection. The study was conducted following the relevant international guidelines, regulations, and principles of Helsinki declaration. Verbal or written consent was not obtained from the study participants due to the fact that the data were collected from patients' medical charts and there was no direct contact between patients and data collectors. Throughout the study, the information obtained from the patient's medical records was kept entirely confidential and only accessed by the research team, and solely for the purpose of the research. For data collection purposes, medical record number were utilized, and no personal identifiers were gathered or utilized in the research report.

**Format for specific study types**<br><br>**Human Subject Research (involving human participants and/or tissue)**<br>• Give the name of the institutional review board or ethics committee that approved the study<br>• Include the approval number and/or a statement indicating approval of this research<br>• Indicate the form of consent obtained (written/oral) or the reason that consent was not obtained (e.g. the data were analyzed anonymously)<br><br>**Animal Research (involving vertebrate animals, embryos or tissues)**<br>• Provide the name of the Institutional Animal Care and Use Committee (IACUC) or other relevant ethics board that reviewed the study protocol, and indicate whether they approved this research or granted a formal waiver of ethical approval<br>• Include an approval number if one was obtained<br>• If the study involved *non-human primates*, add *additional details* about animal welfare and steps taken to ameliorate suffering<br>• If anesthesia, euthanasia, or any kind of animal sacrifice is part of the study, include briefly which substances and/or methods were applied<br><br>**Field Research**<br><br>Include the following details if this study involves the collection of plant, animal, or other materials from a natural setting:<br>• Field permit number<br>• Name of the institution or relevant body that granted permission	
**Data Availability**<br><br>Authors are required to make all data underlying the findings described fully available, without restriction, and from the time of publication. PLOS allows rare exceptions to address legal and ethical concerns. See the PLOS Data Policy and FAQ for detailed information.	Yes - all data are fully available without restriction

A Data Availability Statement describing where the data can be found is required at submission. Your answers to this question constitute the Data Availability Statement and **will be published in the article**, if accepted.<br><br>**Important:** Stating 'data available on request from the author' is not sufficient. If your data are only available upon request, select 'No' for the first question and explain your exceptional situation in the text box.<br><br>Do the authors confirm that all data underlying the findings described in their manuscript are fully available without restriction?	
**Describe where the data may be found in full sentences. If you are copying our sample text, replace any instances of XXX with the appropriate details.**<br><br>• If the data are **held or will be held in a public repository**, include URLs, accession numbers or DOIs. If this information will only be available after acceptance, indicate this by ticking the box below. For example: *All XXX files are available from the XXX database (accession number(s) XXX, XXX.).*<br>• If the data are all contained **within the manuscript and/or Supporting Information files**, enter the following: *All relevant data are within the manuscript and its Supporting Information files.*<br>• If neither of these applies but you are able to provide **details of access elsewhere**, with or without limitations, please do so. For example:<br><br>*Data cannot be shared publicly because of [XXX]. Data are available from the XXX Institutional Data Access / Ethics Committee (contact via XXX) for researchers who meet the criteria for access to confidential data.*<br><br>*The data underlying the results presented in the study are available from (include the name of the third party*	All relevant data are within the manuscript and its Supporting Information files

and contact information or URL).<br>• This text is appropriate if the data are owned by a third party and authors do not have permission to share the data.<br><br>* typeset	
Additional data availability information:	

Powered by Editorial Manager® and ProduXion Manager® from Aries Systems Corporation

Manuscript Click here to access/download;Manuscript;Manuscript PLOS one.docx

Time-to-Recovery from Severe Pneumonia and Its Predictors Among Children 2-59 Months of Age Admitted to Pediatric Ward of Jimma University Medical Center, Southwest, Ethiopia, 2023: A Retrospective Cohort Study.

Getu Girma Bekele[1*], Masrie Getnet[2], Dawit Regassa[2], Tola Getachew Bekele[1]

Authors' Affiliation

[1] Jimma University Medical Center, Jimma, Ethiopia

[2]Jimma University, Institute of Health, Faculty of Public Health, Department of Epidemiology, Jimma, Ethiopia

Authors' Email Addresses

Getu Girma Bekele: Email:getgirma2013@gmail.com, Jimma, Ethiopia

Masrie Getnet: masriegetnet16biostat@gmail.com, Jimma, Ethiopia

Dawit Regassa: dawitregassa4@gmail.com, Jimma, Ethiopia

Tola Getachew Bekele: tolagetachew613@gmail.com, Jimma, Ethiopia

Corresponding Author: Getu Girma Bekele **Email:** getgirma2013@gmail.com

Phone: +251937640136

Abstract

Background: Pneumonia is an inflammation of the lung parenchyma. The World Health Organization estimates that 156 million cases of pneumonia, including up to 20 million cases severe enough to require hospitalization, and 1.2 million deaths each year among under-five children. Ethiopia is ranked sixth among top fifteen countries in terms of pneumonia related morbidity and mortality. The study aimed to assess the time to recovery from severe pneumonia and its predictors among children aged 2-59 months admitted to the pediatric ward of Jimma University Medical Center; Southwest, Ethiopia, 2023.

Methods: A facility-based retrospective cohort study was carried out among 426 children aged between 2 and 59 months. Five years of medical records, from 2018 – 2022, were reviewed. A simple random sampling technique was used. Data entry was done using epidata version 4.6 and exported to and analyzed by STATA version 15. Kaplan Meier's plot was used to compare the survival probability of different groups and the multivariable Cox regression model was used to investigate the independent effect of covariates on recovery time.

Result: The median recovery time was 4 days (IQR: 3, 7). Incidence rate of recovery was 15.78 per 100-person day (95% CI 14.2 – 17.5). The presence of comorbidity (AHR; 0.7, 95% CI (0.54 – 0.91)), being treated with ceftazidime and vancomycin (AHR; 0.29, 95% CI (0.14 – 0.60)), antibiotic change (AHR; 0.74, 95% CI (0.58 – 0.95)) and late presentation to the Hospital (AHR; 0.58, 95% CI (0.43 -0.78)) were statistically significant predictors that prolong recovery time.

Conclusion: The median recovery time was longer than other similar studies. Therefore, due attention should be given concerning the identified predictors of the recovery time.

Keywords: Time to Recovery, Severe Pneumonia, predictors, Children 2-59 Months, Jimma.

INTRODUCTION

The World Health Organization estimates that 156 million cases of pneumonia occur annually in children under the age of five, including up to 20 million cases severe enough to require hospitalization, and 1.2 million deaths each year.(1) The greatest incidence occurring in south Asia (2,500cases per 100,000 children) and west and central Africa (1,620 cases per 100,000 children).(2) Approximately 490,000 children under the age of five died of pneumonia in Sub Saharan Africa (SSA) in 2016.(3) In Ethiopia, approximately 3,370,000 children contract pneumonia each year and accounts for 14% of all deaths of children under five years old.(4)

Severe pneumonia is the major cause of hospitalization in under-five children, among 156,847 admitted children identified in seven hospitals in Bangladesh; pneumonia was the most diagnosed (17.9%).(5) Hospitalization alone was found to contribute to 46.8% of a household price for one severe pneumonia episode.(6) Effective management of pneumonia is not just the ratio of the children recovered, but also the time of recovery. Spending each night in the hospital increases the risk of adverse medication reactions, ulcers, and infections by 0.5%, 0.5%, and 1.6%, respectively.(7) Tremendous independent clinical factors marked up mortality and duration of stay associated with pneumonia, with significant variability among hospitals. Some studies identify different forms of malnutrition, comorbidities, diarrhea, antibiotics first administered, being underweight, and age are the major factors that affect recovery time.(8)

A longer hospital stay has a significant negative impact on the admitted child, his or her parents, and the healthcare system, particularly in societies with resource-limited settings and no health insurance scheme.(9) Between 26.6% and 115.8% of monthly household income is spent on the direct medical costs of treating severe pneumonia in a hospital setting. Non-medical costs (such as travel and food) during a child's hospital stay for severe pneumonia account for 9.0% and 31.0%

of the household's monthly income in Kenya and Guinea, respectively.[10] Patients with prolonged length of stay extremely account for the consumption of more hospital resources, Hospital acquired infection, denies critically ill patients' timely access to treatment and contributes to capacity shortages.[11] Hence, these children are often discharged against medical advice without receiving full therapy, which results in an increased rate of readmission and complications such as pneumonic effusion, empyema, necrotizing pneumonia, and lung abscess.[12]

The Federal Ministry of Health of Ethiopia (FMOHE) has been working to prevent and reduce under-five mortality by introducing Pneumococcal Conjugate Vaccine (PCV) and Haemophilus influenzae type b (Hib) vaccines, as well as by putting into practice strategies such as integrated community case management (ICCM) to train, support, and supply community health workers to provide diagnostic, treatment and referral services and integrated management of newborn and child illnesses (IMNCI) to improve case management skills of health care providers, improving health systems to provide quality care and improving family and community health practice for health, growth, and development.[13, 14] Despite those-mentioned programs and endeavors, still the main causes of illness and death in children under the age of five in Ethiopia is pneumonia; with an approximated contribution of around 40,000 fatalities per year.[15]

The reason the researcher is interested in this study is that pneumonia is one of the major public health importance and despite the intervention that has been implemented in Ethiopia; pneumonia continues as one of the main reasons of hospitalization and mortality among under-five children (9). The widespread nature of the problem in Ethiopia has already killed thousands of children hospitalized with severe pneumonia, which requires everlasting solutions.[16] Understanding the recovery time help the health managers in evaluating the quality of health care delivered by the given health institution because the average length of stay provides information about the

efficiency of care delivery, the standard of care provided, and the effective use of resources. Further, it can provide valuable information to physicians and other healthcare providers for counseling parents or caregivers about the duration of hospitalization so that the caregivers can timely mobilize resources for a child's hospital care.

Up to the best of our knowledge and as far as our literature search, there are limited studies and evidence on the recovery time from severe pneumonia and its predictors in the current study area. As a result, the aim of this study was to estimate the median time of recovery from severe pneumonia and its predictors in children aged 2-59 months admitted to the pediatric ward of Jimma University Medical Center, Southwest, Ethiopia, 2023.

METHODS AND MATERIALS

Study area and Period

The study was conducted at Jimma University Medical Center (JUMC). It is the only teaching and tertiary federal hospital found in southwest Ethiopia. Geographically, it is located in Jimma town, Oromia region, 353 km southwest of Addis Ababa, the capital city of Ethiopia. It was established in 1930 E.C. by Italian invaders for the service of their soldiers. Currently, JUMC provides services for approximately 18,289 inpatients on 800 beds, 232,000 outpatient attendants, 79,000 emergency service cases, and 6,500 deliveries in a year coming to the hospital from the catchment area. JUMC serves as a referral hospital in southwest Ethiopia. The population in the catchment area is estimated to be 15 million.[17, 18]

The pediatric department of JUMC has a pediatric outpatient, pediatric ward, and pediatric intensive care unit. The pediatric ward has three units namely level I, level II, and nutritional rehabilitation unit (NRU) with a total of 78 beds, and provides services for an average of 55-65

patients including severe pneumonia. On average over 400 children were diagnosed with severe pneumonia each year in this hospital. Children diagnosed with severe pneumonia were directly admitted to level I and treated by senior Pediatricians, Residents, Nurses, and Medical Interns. The pedantic ward has a total of 60 health professionals with 35 nurses, 7 senior physicians, and 16 residents. There are also pharmacy professionals and laboratory technologists who were working there by rotation.[18] The five-year data were reviewed from January 1, 2018, to December 31, 2022. The patient charts were retrieved from May 14- June 15, 2023.

Study design

A facility-based retrospective cohort study was conducted.

Population

Source population

All children 2 to 59 months of age who were admitted to the pediatric ward of Jimma University Medical Center (JUMC) with severe pneumonia.

Study population

All children with severe pneumonia aged 2-59 months who were managed at the pediatric ward of Jimma University Medical Center (JUMC) from January 1, 2018- December 31, 2022.

Eligibility Criteria

Inclusion Criteria

All children with severe pneumonia aged 2-59 months who were admitted to the pediatric ward of Jimma University Medical Center (JUMC) from January 1, 2018- December 31, 2022, were included.

Exclusion Criteria

Children's charts with no Medical Registration Number (MRN), admission date, discharge date, and no or ambiguous age were excluded.

Sample size determination

The sample size was calculated by using the Schoenfeld formula for survival analysis by taking the following assumptions into account.

- Significance level (α) (two-sided) =0.05, $Z_{\alpha/2} = 1.96$
- Power = 80%,$\beta = 0.2$ $Z_{\beta=}$0.842
- AHR = Adjusted hazard ratio was used from different similar studies.
- E = number of events (recovery).
- Pr(E) = probability of recovery was used from different similar studies.
- Finally, a 10% non-response rate (NRR) for an incomplete chart was added.
- $E = (z\alpha + z\beta)2$ / (ln AHR)^2 P (1-P), n = E/Pr(E).[19]

The final largest sample size **426** was considered to conduct the study.

Sampling Technique and Procedure

Sampling Technique

A simple random sampling technique was used to select study populations.

Sampling Procedure

The registry logbook of all children who were admitted with severe pneumonia to the pediatric ward of Jimma University Medical Center (JUMC) from January 1, 2018 – December 31, 2022, was reviewed. Since there was no case-specific registration log book, the list of all children aged

2–59 months admitted with severe pneumonia from January 1, 2018 – December 31, 2022, was prepared by excluding those with no MRN, admission date, incomplete and ambiguous age. A total of 1985 children with severe pneumonia were noted from the registration logbook during the study period, of which 1697 completed charts were available. Then, 426 charts were sampled using a simple random sampling technique by a computer-generated method. (Figure 1)

Data collection tool and procedure

Data collection tool

The data was collected by using a structured checklist adapted from previous similar studies.([12, 20, 21, 22]) The tool was designed based on the study objectives and contains socio-demographic characteristics, clinical and treatment-related factors, vaccination and nutritional status as well as comorbidities.

Data collection procedure

The unique medical record number of each patient was used to get their records (charts). All available information on the patient chart was checked. Then all the relevant variables were extracted from patient charts using an English version data extraction tool to meet the study objectives. Two skilled BSc nurses working in the pediatric ward of Jimma University Medical Center (JUMC) extracted the data from patient charts under the supervision of a supervisor who is the MSC holder in pediatric and child health working in the pediatric ward of JUMC as a Head Nurse. Training was given to data extractors and supervisor regarding the significance of the study and ways of the data extraction process by the principal investigator.

Study variables

Outcome variable

Time to recovery from severe pneumonia measured in days.

Independent variables

Socio-demographic characteristics

The socio-demographic characteristics include the age, sex of the children, residence, and health insurance scheme.

Clinical and treatment-related factors

Under clinical and treatment-related factors Presence of Danger signs at admission, the duration time before seeking health care, Grunting, Head nodding, HIV status, Antibiotics first administered, Antibiotic change, and Convulsion were used.

Nutrition and Vaccination status

Nutrition and Vaccination status include Vaccination status, height for age, Weight for height, weight for age, Exclusive breastfeeding, Inability to feed, and different comorbidities.

Operational definition

Severe Pneumonia: children presented with inability to drink, persistent vomiting, oxygen saturation less than 90%, convulsion, and lethargy or reduced level of consciousness.[23]

Time to Recovery: The time from admission to when the child is discharged from the hospital due to improvement in symptoms calculated by number of days.

Median Recovery Time: the time in which half (50%) of the children had recovered.

Censored: A child with severe pneumonia who has been admitted to the ward and happens to be dead, self-discharged, or with unknown outcome status.

Survival Time: is the time starting from the date of admission to recovery from severe pneumonia.

Comorbidity: Any disease acute or chronic present during admission in addition to severe pneumonia.

Danger signs: Children with difficulty breathing, chest in drawing, oxygen saturation less than 90%, unconsciousness (24).

Vaccination Status: Fully Vaccinated which refers to Children who had received all forms of vaccination; Partially Vaccinated (Children who had taken at least one PCV dose) and Unvaccinated (Children who have never received PCV or any form of vaccination).

Discharge status: indicate the condition (reason) for the patient's termination from the program.

Exclusive Breastfeeding: Children who were introduced to only breast milk except for drugs, ORS (Oral Rehydration Solution), during the first six months of age.[25]

Underweight: Children having weight for age is less than -2SD (standard deviation).[26]

Wasting: Children having weight for height is less than -2SD (standard deviation).[27]

Stunting: Children having height for age is less than -2SD (standard deviation).[27]

Data Management

Data Quality Assurance

Data collectors were given orientation on the content of the data extraction tool and procedure. Two skillful BSc nurses working in the pediatric ward of Jimma University Medical Center (JUMC) reviewed the records after they were given training by the principal investigator. Before

the actual data collection, the data extraction checklist was tested on 21 medical charts at Shenen Gibe Hospital to ensure that the data extraction format agreed with the needs of the study. During the data collection process, regular supervision was done by the supervisor to ensure the quality of the data. Throughout the data collection process, the primary investigator and the supervisor routinely examined the accuracy and completeness of the data collected. Data entry and cleaning were done daily and timely feedback was being communicated to the data collectors.

Data analysis procedure

The data were entered using epidata version 4.6 exported to and analyzed using STATA version 15. The data were coded and cleaned before the analysis. The data were described in terms of central tendency (median) and dispersion (interquartile range) for continuous data and frequency distribution for categorical data and presented using graphs, tables, and text. A day was used to calculate the recovery time. Kaplan Meier's plot was used to compare the survival experience of different groups of patients by using survival curves. The association between the independent variable and time to recovery from severe pneumonia was analyzed using the Cox proportional hazard regression model. Log rank test was used to identify the presence of significant difference in median survival time between different groups.

Univariate analysis was performed and predictors that have an association with the outcome variable at a p-value of 0.25 or less were entered into the multivariable Cox regression model. The presence of Multicollinearity was checked using the variance inflation factor. A Variance Inflation Factor value of <10 was used as a cutoff point for indicating the absence of multicollinearity. A Cox proportional hazard assumption was checked for each predictor graphically using a log minus log plot of survival and statistically using the global residual test. The final model adequacy was tested by using the Cox-Snell residual test and the baseline distribution of the cumulative hazard

function of residuals against Cox-Snell residuals was follows 45 degree indicating adequately fitted data. Adjusted hazard ratio (AHR) with a 95% confidence interval (CI) was estimated by multivariable Cox regression and a p-value less than or equal to 0.05 was declared the presence of a statistical association between the recovery time and explanatory variable.

Results

Socio-demographic and treatment-related characteristics of study populations

The study included records of children 2-59 months of age admitted to the pediatric ward of Jimma University Medical Center (JUMC) with a diagnosis of severe pneumonia for five consecutive years (2018-2022). From the samples of 426, information was extracted from 394 patient charts making the response rate 92.5%. The female-to-male ratio was 1:1.21 and 52.28% of the study participants came from the countryside. The median age of the study participants was 10 months IQR (5 - 21) and the majority 218 (55.33%) of them were under the age group of 2-11 months. Almost two third 244 (62%) of the study participants were not enrolled in health insurance schemes.

About 47.72% of the children visited the hospital within three days of becoming ill. Danger signs were present in about 135 (34.26%) of the children at admission. The commonest danger signs were difficulty breathing and severe chest in-drawing accounting for 30 (7.61%) and 25(6.35%) respectively. Among 394 children 188 (47.72%) and 71 (18.02%) had grunting and convulsion respectively. Crystalline penicillin was given to 166 (42.35%) of the children at admission as a first-line treatment and antibiotics were changed for 128 (33%) of the children (Table 1).

Table 1: Frequency distribution of socio-demographic and treatment-related characteristics of children aged between 2-59 months with severe pneumonia admitted to the pediatric ward of Jimma University Medical Center (JUMC) from Jan 1, 2018-Dec 31, 2022(n=394)

Variable	Category	Status	
		Recovered (%)	Censored (%)
Age in month	2-11	196 (49.7)	22 (5.5)
	12-35	111 (28.2)	17 (4.3)
	36-59	42 (10.6)	6 (1.5)
Sex	Male	191 (48.5)	25 (6.3)
	Female	158 (40.1)	20 (5.1)
Danger signs	No	238 (60.4)	21 (5.3)
	Yes	111 (28.2)	24 (6.0)
Residence	Urban	18 (4.5)	170 (43.1)
	Rural	27 (6.8)	179 (45.4)
Antibiotics change	No	229 (59.0)	31 (7.98)
	Yes	114 (29.4)	14 (3.60)
Antibiotic first given	Crystalline penicillin	152 (38.77)	14 (3.57)
	Ampicillin and gentamicin	77 (19.64)	12 (3.06)
	Ceftriaxone	109 (27.80)	16 (4.08)
	Ceftazidime & vancomycin	9 (2.29)	3 (0.76)
Grunting	No	179 (45.4)	27 (6.85)
	Yes	170 (43.1)	18 (4.56)

Vaccination, nutritional status, and comorbidities of the study populations

Most 281 (71.32%) of the children had a history of exclusive breastfeeding. Of the study participants, about 166(42.13%) of the children were fully vaccinated whereas 60(15.23%) were unvaccinated. Stunting, wasting, and being underweight were found in 143 (36.29%), 186 (47.21%), and 161 (40.86%) of the study participants respectively. About 82 (20.81%) of the children were born before the expected date of delivery. Almost 201(51%) of the children tested non-reactive for HIV and 6 (1.53%) of the children among the study participants were found reactive for the HIV test. About 98 (24.87%) of the children admitted with severe pneumonia had different comorbidities. Severe acute malnutrition was one of the most common diseases in addition to severe pneumonia among the study participants. Other medical conditions like Bronchial asthma, Down syndrome, and pertussis accounted for 9 (2.29%) (Table 2)

Table 2: Frequency distribution of vaccination, nutritional status, and comorbidities of children aged between 2-59 months with severe pneumonia admitted to the pediatric ward of Jimma University Medical Center (JUMC) from Jan 1, 2018-Dec 31, 2022(n=394)

Variable	Category	Status	
		Recovered (%)	Censored (%)
Vaccination status	Fully vaccinated	152 (38.57)	14 (3.55)
	Partially vaccinated	151 (38.32)	17 (4.31)
	Unvaccinated	46 (11.67)	14 (3.55)
Weight for height	Normal	183 (46.44)	25 (6.3)
	Wasted	166 (42.13)	20 (5.07)

Exclusive breastfeeding	No	99 (25.12)	14 (3.55)
	Yes	250 (63.45)	31 (7.86)
Weight for age	Normal	210 (53.2)	23 (5.83)
	Underweight	139 (35.2)	22 (5.58)
Height for age	Normal	219 (55.5)	32 (8.12)
	Stunted	130 (32.9)	13 (3.29)
Comorbidities	No	262 (66.5)	34 (8.6)
	Yes	87 (22.1)	11 (2.8)
Vaccination status	Fully vaccinated	152 (38.6)	14 (3.5)
	Partially vaccinated	151 (38.3)	17 (4.3)
	Unvaccinated	46 (11.7)	14 (3.5)
HIV status	Reactive	5 (1.3)	1 (0.2)
	Non-reactive	187 (47.5)	14 (3.5)
	Unknown	157 (39.9)	30 (7.6)

Treatment Outcome and Incidence Rate of Recovery

Regarding the treatment outcome of severe pneumonia, the majority 349 (88.58%) of the children recovered, 22 (5.58%) died, 14 (3.55%) were left against medical advice and 9 (2.28%) were discharged with unknown condition (Figure 2). The shortest and longest length of hospital stay was 1 and 29 days respectively. The total person-day (person time risk) contributed by the study participants was 2,211 days. The overall incidence rate recovery from severe pneumonia was 15.78 per 100-person day (95% CI, 14.2 – 17.5) observation.

The median time to recovery from severe pneumonia

The Kaplan-Meier survival curve was used to estimate the survival status of the children with severe pneumonia. According to the Kaplan-Meier survival estimation, the median survival time without using any stratification factors was 4 days IQR (3, 7). The graph shows a tendency to fall off quickly in the first five days, showing that most children recovered from severe pneumonia during this time period (Figure 3).

Kaplan Meier Survival plot was performed to compare survival probability between categories of different predictors. Accordingly, survival probability among different categorical predictors such as comorbidities, the duration of time before seeking health care, weight for age, antibiotics first administered, and antibiotic change were different (Figure 4).

Comparison of Median Survival Time

To compare the significance difference in median survival times of various categorical predictors, the log-rank test was used. Accordingly, there was a significant difference in median survival time between the categorical predictors like weight for age, height for age, comorbidity, antibiotics first administered, and antibiotics change. For instance, the median recovery time of children without comorbidities was 4 days (IQR, 3 – 6) but for children with comorbidities, it was 6 days (IQR, 4 – 11). Children who visited the Hospital within three days after their illness had a median recovery time of 4 days (IQR, 2 – 5) while those who were visited after seven days was 6 days (IQR, 4 – 13). For children for whom antibiotics were changed, the median recovery time was 5 days (IQR, 3 – 9) whereas for children for whom no antibiotics were changed, the median recovery time was 4 days (IQR, 3 – 6) but, there was no significant variation of median recovery time among categorical predictors like HIV status, residence, grunting, and age.

Proportional Hazard Assumption Test

The Cox proportional hazard assumption was checked by graphically (log-log plot) and statistically (Global test; p-value = 0.37 indicating the Cox proportional hazard assumption was not violated). Finally, Cox-Snell residual test was performed to check the final model adequacy and confirmed that the model is good to fit the data (Figure 5).

Predictors of time to recovery from severe pneumonia

After running univariate Cox regression, predictors such as Sex, age, residence, grunting, antibiotic first administered, antibiotic change, vaccination status, height for age, weight for age, weight for height, comorbidities, and duration of time before seeking healthcare were selected as a candidate variable for multivariable Cox regression analysis. After carrying out the multivariable Cox regression, hazard ratios with a 95% confidence interval (CI) and a p-value less than or equal to 0.05. Thus, Variables like the presence of comorbidities, duration of time before seeking health care, antibiotic first administered and antibiotics change were statistically significant predictors of time to recovery from severe pneumonia in this study.

The recovery time of children with comorbidity is delayed by 30% as compared to children without comorbidity (AHR; 0.70, (95% CI, (0.54 - 0.92)). Regarding the treatment regimen, the recovery time of children treated with ceftazidime and vancomycin as first-line antibiotics during admission was delayed by 71% as compared to children treated with crystalline penicillin (AHR; 0.29, (95% CI, 0.145 - 0.614)). Children for whom antibiotics were changed were 26% less likely to recover quickly than children for whom antibiotics were not changed (AHR; 0.74, (95% CI, 0.57 - 0 .94)). Children with severe pneumonia who came between 4 and 7 days to the Hospital had 27% slower recovery time as compared to children who came within 3 days (AHR; 0.73(95% CI, (0.561-

0.959)) and those who came after 7 days of their illness to the Hospital had 42% slower recovery time as compared to the reference group (AHR; 0.58, (95% CI, 0.43 - 0.79)) (Table 3).

Table 3: Multivariable Cox regression analysis for independent predictors of recovery time among children aged 2-59 months with severe pneumonia admitted to the pediatric ward of Jimma University Medical Center (JUMC) from Jan 1, 2018- Dec 31, 2022

Variable	Category	p-value	CHR, 95%CI	AHR, 95%CI
Sex	Male		1	1
	Female	0.038	0.79(0.645-0.988)	0.92(0.727 - 1.155)
Residence	Urban		1	1
	Rural	0.225	0.87(0.711-1.083)	0.94 (0.752 - 1.175)
Age	2-11 months		1	1
	12-35 months	0.226	0.86(0.684-1.093)	1.00(0.736 - 1.382)
	36-59 months	0.204	0.80(0.576-1.124)	1.05(0.678 - 1.636)
Grunting	No		1	1
	Yes	0.122	0.84(0.686-1.045)	0.85 (0 .680 - 1.071)
Antibiotics first administered	Cr. penicillin		1	1
	Ampicillin & Gentamycin	0.000	0.50(0.379-0.665)	**0.59(0.439- 0.798) ****
	Ceftriaxone	0.000	0.55(0.432-0.714)	**0.56(0.426 - 0.727) ****
	Ceftazidime & Vancomycin	0.000	0.23(0.117-0.457)	**0.29(0.143- 0.600) ****

Antibiotic change	No<br>Yes	 0.001	1<br>0.66(0.532-0.839)	1<br>**0.74(0.581 - 0.953) ***
Vaccination status	Fully vaccinated<br>Partially vaccinated<br>unvaccinated	 0.734<br>0.001	1<br>1.04(0.829-1.303)<br>0.57(0.411-0.799)	1<br>1.01 (0.728 - 1.402)<br>0.70(0.489 - 1.029)
Time prior to seeking health care	Up to 3 days<br>4 - 7days<br>Above 7 days	 0.003<br>0.000	1<br>0.67(0.525-0.872)<br>0.51(0.392-0.669)	1<br>**0.73 (0.561- 0.959) ***<br>**0.58 (0.432 - 0.789) ****
Comorbidities	No<br>Yes	 0.000	1<br>0.60(0.473-0.774)	1<br>**0.70 (0.543 - 0.914) ***
Height for age	Normal<br>Stunted	 0.059	1<br>0.81(0.651-1.007)	1<br>0.85(0.671- 1.072)
Weight for height	Normal<br>Wasted	 0.055	1<br>1.23(.995-1.528)	1<br>1.15(0.919 - 1.439)
Weight for age	Normal<br>Underweight	 0.003	1<br>0.71(0.576-0.890)	1<br>0.89(0.699 - 1.134)

P-Value =< 0.05(*) and ** for p-value < 0.01

Discussion

Children between the ages of 2-59 months with severe pneumonia who were admitted to the pediatric ward of Jimma University Medical Center (JUMC) were assessed for recovery time and its predictors. In the current study, the median recovery time from severe pneumonia was 4 days IQR (3, 7). Different predictors were found to contribute to the recovery time of children admitted with severe pneumonia in this study. Predictors like antibiotic first administered, antibiotic change, duration of time before seeking health care, and comorbidity had significant associations with recovery time.

According to the current study, among children who were admitted to the pediatric ward of Jimma University Medical Center between the ages of 2-59 months, the median recovery time from severe pneumonia was 4 days. This finding is in line with other studies conducted in Debre Markos[(28)], Hosanna [(8)] and Gambia. [(12)] This finding is longer than the British Thoracic Society guideline [(29)] and the study conducted in Nepal that estimates the recovery time of 2 days.[(30)] The gap may be explained by the difference in socioeconomic status of the research areas, unique circumstances, such as treatment protocols, and the age of the study participants, as the study involved Nepalese children between the ages of 2-35 months.

The current finding is lower than a study conducted in Tanzania which estimated the median recovery time of 10 days.[(31)] The discrepancy could be a result of the time difference between study periods. The difference in the age of the study population could also explain the discrepancy, as the Tanzanian study was conducted among children aged between the ages of 0 and 144 months. The analysis approach (model) used, as the Tanzanian study was logistic regression, which does not take the censoring of study participants into account and the study design as they conducted a prospective cohort study which is better in generating strong evidence could explain the variability.

According to the study conducted in Poland, patients stay an average of 8.2 to 10.1 days.[32] This variation might be due to the fact that in our country Ethiopia, the bed occupancy rate is high and patients are discharged, as soon as their signs and symptoms subside without sending and waiting for any laboratory confirmation but, this country is high-income country compared to our country, and their health care system might be well organized and developed. Due to that the bed occupancy rate is probably low and people can pay any charge at the hospital; this may create an opportunity for patients to stay longer at the hospital until complete stability from all signs and symptoms of the disease.

Duration of time before seeking health care was an independent significant predictor of the recovery time of severe pneumonia. Children who came to the hospital within three days of illness recovered quicker than those children who came after seven days of illness. This finding is supported by a prospective follow-up study conducted in the Gambia [12] and other retrospective studies conducted in Debre Markos Hospital [28], Ayder Comprehensive Specialized Hospital [22], and Central and North Gondar zones.[33] This could be because the late presenter might develop a complication that needs further investigation to treat and also as the condition of the disease increases, the length of time required to recover from it increases.[34]

The existence of co-morbidity was another significant predictor that was connected with the length of time needed to recover from severe pneumonia in this study. Those children with any type of co-morbidity were found to stay longer in the hospital than their counterparts who were free of comorbidities. This finding is consistent with other studies conducted at Debre Markos [28], Central and North Gondar zones [33], and Nepal.[30] The reason why children with comorbidity stay for a prolonged time at the hospital might be due to the fact that Comorbidities significantly increase the severity of pneumonia, including the need for oxygen, and are significantly associated with

more complications, thus reducing the recovery time.[31] Since the major comorbidity in the current study was severe acute malnutrition, as adequate protein and energy are needed for proper immune system functioning; undernourished children have a compromised immune system and are more vulnerable to developing serious infections and complications could be possible explanation for a prolonged stay among acutely malnourished children.[35]

The antibiotic first administered was one of the significant clinical and treatment-related predictors in this study. Children with severe pneumonia who were treated with ceftriaxone and ampicillin and gentamicin had a prolonged recovery time. This might be due to ampicillin and gentamicin being administered together for patients with bacterial meningitis after testing for cerebrospinal fluid (CSF)[36] and for those children who presented with severe acute malnutrition in addition to severe pneumonia.[37] Children with severe pneumonia who were treated with ceftazidime and vancomycin as the first antibiotic at the time of admission stayed longer in the hospital. This might be because these drugs are used to treat seriously ill children and infections caused by staphylococcus aureus and gram-negative enteric bacilli pneumonia, especially those with penicillin resistance properties. Furthermore, ceftazidime and vancomycin together administered for children with comorbidity, particularly for severe bacterial infections like endocarditis. [38]

Antibiotic change is also a significant predictor of recovery time. Compared to children whose medications were not altered, those whose antibiotics were changed required lengthier hospital stays to recover from severe pneumonia. Antibiotics are altered whenever there is treatment failure on first-line drugs due to drug resistance or selection of inappropriate drug to treat the patients.[39] For this reason, the additional time required by second-line drugs to act could explain the longer recovery time. This finding is supported by other study conducted in Hosanna.[8]

Strength and Limitations of the Study

Although this study uses five years of data, important variables like parent's income and educational status were not included. The study also didn't take any laboratory investigation into account like baseline complete blood count, blood culture, and electrolyte sedimentation rate.

Conclusion

This study assessed the recovery time from severe pneumonia and its predictors among children aged 2-59 months admitted to the pediatric ward of Jimma University Medical Center (JUMC). The finding of this study revealed that the median recovery time was longer than other similar studies. Late presentation to the hospital, antibiotic first administered, antibiotic change and the presence of comorbidities were significant predictors that prolonged the recovery time. Healthcare providers should have to give due attention to the identified predictors of recovery time. Measures that help children to recover quickly from their illness should be strengthened.

Abbreviations

AHR: Adjusted Hazard Ratio; **BSc:** Bachelor of Science; **CEO:** Chief Executive Officer; **CI:** Confidence Interval; **CHR**: Crude Hazard Ratio; **CSF:** Cerebrospinal Fluid; **FMOHE:** Federal Ministry of Health of Ethiopia; **HIV:** Human Immune Virus; **Hib:** Haemophilus influenzae type b; **ICCM:** Integrated Community Case Management**; IMNCI:** Integrated Management of Newborn and Childhood Illness**; IQR:** Interquartile range**; JUMC:** Jimma University Medical Center**; MRN:** Medical Registration Number**; ORS:** Oral Rehydration Solution**; PCV:** Pneumococcal Conjugate Vaccine**; SAM:** Severe Acute Malnutrition**; SD:** Standard Deviation**; SSA:** Sub-Saharan Africa**; TB:** Tuberculosis**; WHO:** World Health Organization

Ethical Considerations

Before data collection, this research protocol has been submitted to the Institutional Review Board (IRB) of Jimma University and meets the ethical and scientific standards outlined in national and international guidelines. Jimma University, Institute of Health (Reference No JUIH/IRB/361/23) approved the consent wavier. The letter of permission was written to Jimma University Medical Center managers. Letter to access the patient's chart was obtained from the chief executive officer (CEO) of Jimma University Medical Center and the health management information system focal person verbally consented to use the patients' medical chart for data collection. The study was conducted following the relevant international guidelines, regulations, and principles of Helsinki declaration. Verbal or written consent was not obtained from the study participants due to the fact that the data were collected from patients' medical charts and there was no direct contact between patients and data collectors. Throughout the study, the information obtained from the patient's medical records was kept entirely confidential and only accessed by the research team, and solely for the purpose of the research. For data collection purposes, medical record number were utilized, and no personal identifiers were gathered or utilized in the research report.

Acknowledgments

The authors would like to acknowledge Jimma University Ethical Review Board for providing the ethical clearance letter and Shenen Gibe General Hospital for their valuable and preliminary support. We also extend our heart full gratitude to all the data collectors and supervisors for their efforts in the data collection and chart review process.

Funding

No funds were obtained for this particular study.

Disclosure

No conflict of interest in this particular study.

References

1. McAllister DA, Liu L, Shi T, Chu Y, Reed C, Burrows J, et al. Global, regional, and national estimates of pneumonia morbidity and mortality in children younger than 5 years between 2000 and 2015: a systematic analysis. Lancet Glob Health. 2019;7(1):e47-e57.

2. Rudan I, O'Brien KL, Nair H, Liu L, Theodoratou E, Qazi S, et al. Epidemiology and etiology of childhood pneumonia in 2010: estimates of incidence, severe morbidity, mortality, underlying risk factors and causative pathogens for 192 countries. J Glob Health. 2013;3(1):010401.

3. UNICEF. A fair chance for every child. 2016. 2016.

4. Pneumonia in children 2019 [Available from: https://www.who.int/news.

5. Naheed A, Breiman RF, Islam MS, Saha SK, Tabassum Naved R. Disparities by sex in care-seeking behaviors and treatment outcomes for pneumonia among children admitted to hospitals in Bangladesh. PLoS One. 2019;14(3):e0213238.

6. Madsen HO, Hanehøj M, Das AR, Moses PD, Rose W, Puliyel M, et al. Costing of severe pneumonia in hospitalized infants and children aged 2-36 months, at a secondary and tertiary level hospital of a not-for-profit organization. Tropical Medicine & International Health. 2009;14(10):1315-22.

7. Hauck K, Zhao X. How Dangerous is a Day in Hospital?: A Model of Adverse Events and Length of Stay for Medical Inpatients. Medical Care. 2011;49(12):1068-75.

8. Tirore LL, Abame DE, Sedoro T, Ermias D, Arega A, Tadesse T, et al. Time to Recovery from Severe Pneumonia and Its Predictors Among Children 2–59 Months of Age Admitted to Pediatric Ward of Nigist Eleni Mohammed Memorial Comprehensive Specialized Hospital, Hossana, Ethiopia: Retrospective Cohort Study. Pediatric Health, Medicine and Therapeutics. 2021;Volume 12:347-57.

9. Mulholland E, Smith L, Carneiro I, Becher H, Lehmann D. Equity and child-survival strategies. Bull World Health Organ. 2008;86(5):399-407.

10. Zhang S, Sammon PM, King I, Andrade AL, Toscano CM, Araujo SN, et al. Cost of management of severe pneumonia in young children: systematic analysis. J Glob Health. 2016;6(1):010408.

11. Marfil-Garza BA, Belaunzaran-Zamudio PF, Gulias-Herrero A, Zuniga AC, Caro-Vega Y, Kershenobich-Stalnikowitz D, et al. Risk factors associated with prolonged hospital length-of-stay: 18-year retrospective study of hospitalizations in a tertiary healthcare center in Mexico. PLoS One. 2018;13(11):e0207203.

12. Kuti BP, Adegoke SA, Oyelami OA, Ota MO. Predictors of prolonged hospitalisation in childhood pneumonia in a rural health centre. South African Journal of Child Health. 2014;8(1).

13. Black RE, Morris SS, Bryce J. Where and why are 10 million children dying every year? Lancet. 2003;361(9376):2226-34.

14. WHO. Revised WHO classification and treatment of childhood pneumonia at health facilities.

15. Alamneh YM, Adane F. Magnitude and Predictors of Pneumonia among Under-Five Children in Ethiopia: A Systematic Review and Meta-Analysis. J Environ Public Health. 2020;2020:1606783.

16. Tegenu K, Geleto G, Tilahun D, Bayana E, Bereke B. Severe pneumonia: Treatment outcome and its determinant factors among under-five patients, Jimma, Ethiopia. SAGE Open Med. 2022;10:20503121221078445.

17. Jimma University Medical Center (JUMC): lucca incrocci foundation; [Available from: https://www.lucaincroccifoundation.org/JUMC.

18. Jimma university medical center health managment information system. 2023.

19. D D. Sample-Size Formula for the Proportional-Hazards Regression Model. 1983.

20. Tamirat ZG, Gelagay AA, Boke MM. Time to recovery and its predictors among children age of 2 up to 59 months admitted with severe community acquired pneumonia in Public Hospitals of Central and North Gondar Zones, Ethiopia 2021. 2022.

21. Tirore LL, Abame DE, Sedoro T, Ermias D, Arega A, Tadesse T, et al. Time to Recovery from Severe Pneumonia and Its Predictors Among Children 2-59 Months of Age Admitted to Pediatric Ward of Nigist Eleni Mohammed Memorial Comprehensive Specialized Hospital, Hossana, Ethiopia: Retrospective Cohort Study. Pediatric Health Med Ther. 2021;12:347-57.

22. Amare RA, Fisseha G, Berhe A, Tirore LL. Incidence of recovery from severe pneumonia and its predictors among children 2-59 months admitted to pediatric ward of Ayder Comprehensive Specialized Hospital, Tigray, Ethiopia: A retrospective Cohort study. J Family Med Prim Care. 2022;11(9):5285-92.

23. WHO. pneumonia fact sheet. American thoracic society. 2016.

24. Ndu IK, Ekwochi U, Osuorah CD, Onah KS, Obuoha E, Odetunde OI, et al. Danger Signs of Childhood Pneumonia: Caregiver Awareness and Care Seeking Behavior in a Developing Country. Int J Pediatr. 2015;2015:167261.

25. Ayalew T. Exclusive breastfeeding practice and associated factors among first-time mothers in Bahir Dar city, North West Ethiopia, removed: A community based cross sectional study. Heliyon. 2020;6(9):e04732.

26. Tiruneh CM, Emiru TD, Tibebu NS, Abate MW, Nigat AB, Bantie B, et al. Underweight and its associated factors among pediatrics attending HIV Care in South Gondar Zone public health facilities, Northwest Ethiopia, 2021. BMC Pediatr. 2022;22(1):575.

27. Luzingu JK, Stroupe N, Alaofe H, Jacobs E, Ernst K. Risk factors associated with under-five stunting, wasting, and underweight in four provinces of the Democratic Republic of Congo: analysis of the ASSP project baseline data. BMC Public Health. 2022;22(1):2422.

28. Mengist B, Tesfa M, Kassie B. Time to recovery and predictors of severe community-acquired pneumonia among pediatric patients in Debre Markos referral hospital, North West Ethiopia: A retrospective follow-up study. PLoS One. 2020;15(9):e0239655.

29. Harris M, Clark J, Coote N, Fletcher P, Harnden A, McKean M, et al. British Thoracic Society guidelines for the management of community acquired pneumonia in children: update 2011. Thorax. 2011;66 Suppl 2:ii1-23.

30. Basnet S, Sharma A, Mathisen M, Shrestha PS, Ghimire RK, Shrestha DM, et al. Predictors of duration and treatment failure of severe pneumonia in hospitalized young Nepalese children. PLoS One. 2015;10(3):e0122052.

31. Caggiano S, Ullmann N, De Vitis E, Trivelli M, Mariani C, Podagrosi M, et al. Factors That Negatively Affect the Prognosis of Pediatric Community-Acquired Pneumonia in District Hospital in Tanzania. Int J Mol Sci. 2017;18(3).

32. Gajewska M, Lewtak K, Scheres J, Albrecht P, Gorynski P. Trends in Hospitalization of Children with Bacterial Pneumonia in Poland. Cent Eur J Public Health. 2016;24(3):188-92.

33. Tamirat ZG, Gelagay AA, Boke MM. Time to recovery and its predictors among children age of 2 up to 59 months admitted with severe community acquired pneumonia in Public Hospitals of Central and North Gondar Zones, Ethiopia 2021. 2022.

34. MacKian S. A review of health seeking behaviour: problems and prospects. 2018.

35. Kidane GF, Zereabruk K, Aberhe W, Hailay A, Mebrahtom G, Gebremeskel GG, et al. Time to recovery from severe acute malnutrition and its predictors among under five children admitted to therapeutic feeding units of general and referral hospitals in Tigray, Ethiopia, 2020: a prospective cohort study. BMC Pediatr. 2023;23(1):325.

36. Asghar R, Banajeh S, Egas J, Hibberd P, Iqbal I, Katep-Bwalya M, et al. Chloramphenicol versus ampicillin plus gentamicin for community acquired very severe pneumonia among children aged 2-59 months in low resource settings: multicentre randomised controlled trial (SPEAR study). BMJ. 2008;336(7635):80-4.

37. UNICEF. Management of Severe Acute Malnutrition in children: working towards results at scale. 2015.

38. Matsumoto K, Takesue Y, Ohmagari N, Mochizuki T, Mikamo H, Seki M, et al. Practice guidelines for therapeutic drug monitoring of vancomycin: a consensus review of the Japanese Society of Chemotherapy and the Japanese Society of Therapeutic Drug Monitoring. J Infect Chemother. 2013;19(3):365-80.

39. Muro RP, Masoza TS, Kasanga G, Kayange N, Kidenya BR. Predictors and outcome of first line treatment failure among under-five children with community acquired severe pneumonia at Bugando Medical Centre, Mwanza, Tanzania: A prospective cohort study. PLoS One. 2020;15(12):e0243636.

Manuscript Click here to access/download;Manuscript;Manuscript.docx

Time-to-Recovery from Severe Pneumonia and its Predictors among Children 2-59 Months of Age Admitted to Pediatric Ward of Jimma University Medical Center, Southwest Ethiopia, 2023: A Retrospective Cohort Study

Getu Girma Bekele[1*], Masrie Getnet[2], Dawit Regassa[2], Tola Getachew Bekele[1]

Authors' Affiliation

[1] Jimma University Medical Center, Jimma, Ethiopia

[2]Jimma University, Institute of Health, Faculty of Public Health, Department of Epidemiology, Jimma, Ethiopia

Authors' Email Addresses

Getu Girma Bekele: Email:getgirma2013@gmail.com, ORCID: https://orcid.org/0009-0003-2864-4414, Jimma, Ethiopia

Masrie Getnet: masriegetnet16biostat@gmail.com, Jimma, Ethiopia

Dawit Regassa: dawitregassa4@gmail.com, Jimma, Ethiopia

Tola Getachew Bekele: tolagetachew613@gmail.com, ORCID: https://orcid.org/0000-0003-1374-0166, Jimma, Ethiopia

Corresponding Author: Getu Girma Bekele **Email:** getgirma2013@gmail.com

Phone: +251937640136

Abstract

Background: Pneumonia is an inflammation of lung parenchyma. The World Health Organization estimated 156 million cases of pneumonia occur annually. Out of them, 20 million cases severe enough to require hospitalization, and each year 1.2 million deaths occur among under-five children. Despite studies and initiatives aimed at reducing pneumonia related deaths in children, Ethiopia is ranked sixth among top fifteen countries in terms of pneumonia related morbidity and mortality.

Objectives: This study aimed to assess the time to recovery from severe pneumonia and its predictors among children aged 2-59 months admitted to the pediatric ward of Jimma University Medical Center; Southwest, Ethiopia, 2023.

Methods: A facility-based retrospective cohort study was carried out among 426 children aged between 2 and 59 months. Five years of medical records, from 2018 – 2022, were reviewed. A simple random sampling technique was used. Data entry was done in epidata version 4.6 and exported to and analyzed by STATA version 15. Variables with p-value < 0.25 at Bivariable Cox regression analysis were selected for the multivariable Cox proportional model . A multivariable Cox regression model with 95% confidence interval and Adjusted Hazard Rtio was used to identify a significant predictor of time to recovery at a p-value < 0.05.

Result: The median recovery time was 4 days (IQR: 3, 7). Incidence rate of recovery was 15.78 per 100-person day (95% CI 14.2 – 17.5). The presence of co-morbidity (AHR; 0.7, 95% CI (0.54 – 0.91)), being treated with ceftazidime and vancomycin (AHR; 0.29, 95% CI (0.14 – 0.60)), antibiotic change (AHR; 0.74, 95% CI (0.58 – 0.95)) and late presentation to the Hospital (AHR; 0.58, 95% CI (0.43 -0.78)) were statistically significant predictors that prolong recovery time.

Conclusion: The median recovery time was longer than other similar studies. Therefore, due attention should be given to the identified predictors of the recovery time.

Keywords: Time to recovery, Severe Pneumonia, predictors, Children 2-59 Months, Jimma.

INTRODUCTION

The World Health Organization estimated that 156 million cases of pneumonia occur annually in children under age of five, including up to 20 million cases severe enough to require hospitalization, and 1.2 million deaths each year.(1) The greatest incidence was occurring in south Asia (2,500cases per 100,000 children) and west and central Africa (1,620 cases per 100,000 children).(2) Approximately 490,000 children under the age of five died of pneumonia in Sub Saharan Africa (SSA) in the year 2016.(3) In Ethiopia, approximately 3,370,000 children contract pneumonia each year which accounts for 14% of all deaths of children under five years old. (4)

Severe pneumonia is the major cause of hospitalization in under-five children. Among 156,847 admitted children identified in seven hospitals in Bangladesh, pneumonia was the most diagnosed (17.9%).(5) Hospitalization alone was found to contribute to 46.8% of a household price for one severe pneumonia episode.(6) Effective management of pneumonia is not just only the ratio of the children recovered, but also the time of recovery. Spending each night in the hospital increases the risk of developing adverse medication reactions, ulcers, and infections by 0.5%, 0.5%, and 1.6%,

respectively.[7] Tremendous independent clinical factors marked up mortality and duration of stay associated with pneumonia, with significant variability among hospitals. Some studies identify different forms of malnutrition, co-morbidities, diarrhea, antibiotics first administered, being underweight, and age are the major factors that affect recovery time.[8]

A longer hospital stay has a significant negative impact on the admitted child, his or her parents, and the healthcare system, particularly in societies with resource-limited settings and no health insurance scheme.[9] Between 26.6% and 115.8% of monthly household income is spent on the direct medical costs of treating severe pneumonia in a hospital setting. Non-medical costs (such as travel and food) during a child's hospital stay for severe pneumonia account for 9.0% and 31.0% of the household's monthly income in Kenya and Guinea, respectively.[10] Patients with prolonged length of stay extremely account for the consumption of more hospital resources, Hospital acquired infection, and denies critically ill patients' timely access to treatment and contributes to capacity shortages.[11] Hence, these children are often discharged against medical advice without receiving full therapy, which results in an increased rate of readmission and complications such as pneumonic effusion, empyema, necrotizing pneumonia, and lung abscess.[12]

The Federal Ministry of Health of Ethiopia (FMOHE) has been working to prevent and reduce under-five mortality by introducing Pneumococcal Conjugate Vaccine (PCV) and Haemophilus influenzae type b (Hib) vaccines. The ministry is also implementing key strategies such as integrated community case management (ICCM) to train, support, and supply community health workers to enhance diagnostic, treatment and referral services and integrated management of newborn and child illnesses (IMNCI) to improve case management skills of health care providers, improving health systems to provide quality care and improving family and community health practice for health, growth, and development.[13, 14] Ethiopian Health Sector Transformation Plan

in 2015, to reduce the mortality rate of children to 31 and 41 per 1000 live births in 2025 and 2035 respectively.(15) Despite these program intervention, pneumonia remain the major cause and of illness and death in children under age of five in Ethiopia; with an approximated contribution of around 40,000 fatalities per year.(16)

Pneumonia is one of the major public health importances. Despite the intervention that has been implemented in Ethiopia, pneumonia continues as one of the main reasons of hospitalization and mortality among under-five children.(9) The widespread nature of the problem in Ethiopia has already killed thousands of children hospitalized with severe pneumonia, which requires everlasting solutions.(17) Understanding the average length of stay in hospitals helps the health managers in evaluating the quality of health care delivered by a given health institution because the average length of stay indicates information about efficiency of care service delivery, and the standard of care being provided, and effective use of resources. Furthermore, it can also provide valuable information to physicians and other healthcare providers to counsel parents or caregivers about the duration of hospitalization so that the caregivers can timely mobilize resources for a child's hospital care.

To the best of our knowledge and as far as our literature search is concerned, there are limited studies and evidence on recovery time from severe pneumonia and its predictors in the current study area. As a result, the aim of this study was to estimate the median time of recovery from severe pneumonia and its predictors in children aged 2-59 months admitted to the pediatric ward of Jimma University Medical Center, Southwest, Ethiopia, 2023.

MATERIALS AND METHODS

Study Area and Period

The study was conducted at Jimma University Medical Center (JUMC). It is the only teaching and tertiary federal hospital found in southwest Ethiopia. Geographically, it is located in Jimma town, Oromia region, 353 km southwest of Addis Ababa, the capital city of Ethiopia. It was established in 1930 E.C. by Italian invaders for the service of their soldiers. Currently, JUMC provides services for approximately 18,289 inpatients on 800 beds, 232,000 outpatient attendants, 79,000 emergency service cases, and 6,500 deliveries in a year coming to the hospital from the catchment area. JUMC serves as a referral hospital in southwest Ethiopia. The population in the catchment area is estimated to be 15 million.(18, 19)

The pediatric department of JUMC has a pediatric outpatient, pediatric ward, and pediatric intensive care unit. The pediatric ward has three units namely level I, level II, and nutritional rehabilitation unit (NRU) with a total of 78 beds, and provides services for an average of 55-65 patients including severe pneumonia. On average over 400 children were diagnosed with severe pneumonia each year in this hospital. Children diagnosed with severe pneumonia were directly admitted to level I and treated by senior Pediatricians, Residents, Nurses, and Medical Interns. The pediatric ward has a total of 60 health professionals with 35 nurses, 7 senior physicians, and 16 residents. There are also pharmacy professionals and laboratory technologists who were working there by rotation.(19) The five-year data were reviewed from January 1, 2018, to December 31, 2022. The patient charts were retrieved from May 14- June 15, 2023.

Study Design

A facility-based retrospective cohort study was conducted.

Population

Source population

All children 2 to 59 months of age who were admitted to the pediatric ward of Jimma University Medical Center (JUMC) with severe pneumonia were the target population.

Study population

All children with severe pneumonia aged 2-59 months who were managed at the pediatric ward of Jimma University Medical Center (JUMC) from January 1, 2018- December 31, 2022 were the study population.

Eligibility Criteria

Inclusion criteria

All children with severe pneumonia aged 2-59 months who were admitted to the pediatric ward of Jimma University Medical Center (JUMC) from January 1, 2018- December 31, 2022, were included.

Exclusion criteria

Children's charts with no Medical Registration Number (MRN), admission date, discharge date, and no or ambiguous age were excluded.

Sample size determination

The sample size was calculated by using the Schoenfeld formula for survival analysis by taking the following assumptions into account.[20]

- Significance level (α) (two-sided) =0.05, $Z_{\alpha/2} = 1.96$
- Power = 80%,$\beta = 0.2$ $Z_{\beta=}$0.842
- AHR = Adjusted hazard ratio was used from different similar studies

- E = number of events (recovery)
- Pr(E) = probability of recovery was used from different similar studies
- Finally, a 10% non-response rate (NRR) for an incomplete chart was added
- $E = (z\alpha + z\beta)2$ / (ln AHR) ^{2} P (1-P), n = E/Pr(E)[20]

The final largest sample size **426** was considered to conduct the study.

Sampling Technique and Procedure

Sampling technique

A simple random sampling technique was used to select the study populations.

Sampling procedure

The registry logbook of all children who were admitted with severe pneumonia to the pediatric ward of Jimma University Medical Center (JUMC) from January 1, 2018 – December 31, 2022, was reviewed. Since there was no case-specific registration log book, the list of all children aged 2–59 months admitted with severe pneumonia from January 1, 2018 – December 31, 2022, was prepared by excluding those with no MRN, admission date, discharge date, incomplete and ambiguous age. A total of 1985 children with severe pneumonia were noted from the registration logbook during the study period, of which 1697 completed charts were available. Then, 426 charts were sampled using a simple random sampling technique via a computer-generated method (Figure 1).

Data collection tool and procedure

Data collection tool

The data were collected by using a structured checklist adapted from previous similar studies.[8, 12, 21, 22] The tool was designed based on the study objectives and contains socio-demographic

characteristics, clinical and treatment-related factors, vaccination and nutritional status as well as co-morbidities.

Data collection procedure

The unique medical record number of each patient was used to get their records (charts). All available information on the patient chart was checked. Then all the relevant variables were extracted from patient charts using an English version data extraction tool to meet the study objectives. Two skilled BSc nurses working in the pediatric ward of Jimma University Medical Center (JUMC) extracted the data from patient charts under the supervision of an MSc holder in pediatric and child health working in the pediatric ward of JUMC as a Head Nurse. Training was given to data extractors and supervisor regarding the significance of the study and ways of the data extraction process by the principal investigator.

Study variables

Outcome variable

Time to recovery from severe pneumonia measured in days.

Independent variables

Socio-demographic characteristics

The socio-demographic characteristics include the age, sex of the children, residence, and health insurance scheme.

Clinical and treatment-related factors

Under clinical and treatment-related factors Presence of Danger signs at admission, the duration time before seeking health care, Grunting, Head nodding, HIV status, Antibiotics first administered, Antibiotic change, and Convulsion were used.

Nutrition and Vaccination status

Nutrition and Vaccination status include Vaccination status, height for age, Weight for height, weight for age, Exclusive breastfeeding, Inability to feed, and different co-morbidities.

Operational definition

Severe Pneumonia: children presented with inability to drink, persistent vomiting, oxygen saturation less than 90%, convulsion, and lethargy or reduced level of consciousness.[(23)]

Time to Recovery: The time from admission to when the child is discharged from the hospital due to improvement in symptoms calculated by number of days.

Median Recovery Time: the time in which half (50%) of the children had recovered.

Censored: A child with severe pneumonia who has been admitted to the ward and happens to be dead, self-discharged, or with unknown outcome status.

Comorbidity: Any disease acute or chronic present during admission in addition to severe pneumonia.

Danger signs: Children with difficult breathing, chest in drawing, oxygen saturation less than 90%, unconsciousness.[(24)]

Vaccination Status: Fully Vaccinated which refers to Children who had received all forms of vaccination; Partially Vaccinated (Children who had taken at least one PCV dose) and Unvaccinated (Children who have never received PCV or any form of vaccination).

Discharge status: indicate the condition (reason) for the patient’s termination from the program.

Exclusive Breastfeeding: Children who were introduced to only breast milk except for drugs, ORS (Oral Rehydration Solution), during the first six months of age.[(25)]

Underweight: Children having weight for age is less than -2SD (standard deviation).[26]

Wasting: Children having weight for height is less than -2SD (standard deviation).[27]

Stunting: Children having height for age is less than -2SD (standard deviation).[27]

Data Management

Data Quality Assurance

Data collectors were given orientation on the content of the data extraction tool and procedure. Two skillful BSc nurses working in the pediatric ward of Jimma University Medical Center (JUMC) reviewed the records after they were given training by the principal investigator. Prior to actual data collection, the data extraction checklist was tested on 21 medical charts at Shenen Gibe Hospital to ensure that the data extraction format is agreed with the objectives of the study. During the data collection process, regular supervision was performed by the supervisor to ensure the quality of data. Throughout the data collection process, the primary investigator and the supervisor routinely examined the accuracy and completeness of the data collected. Data entry and cleaning were done daily and timely feedback was being provided to the data collectors.

Data analysis procedure

The data were entered in epidata version 4.6 exported to and analyzed using STATA version 15. The data were coded and cleaned prior to analysis. The data were described in terms of central tendency (median) and dispersion (interquartile range) for continuous data and frequency distribution for categorical data and presented using graphs, tables, and text. A day was used to calculate the recovery time. Kaplan Meier's plot was used to compare the survival experience of different groups of patients by using survival curves. The association between the independent variable and time to recovery from severe pneumonia was analyzed using the Cox proportional

hazard regression. Log rank test was used to identify the presence of significant difference in median survival time between different groups.[28]

Univariate analysis was performed and predictors that have an association with the outcome variable at a p-value of 0.25 or less were entered into the multivariable Cox regression model. The presence of Multicollinearity was checked using the variance inflation factor (VIF). A Variance Inflation Factor value of <10 was used as a cutoff point for indicating the absence of multicollinearity. A Cox proportional hazard assumption was checked statistically using the global residual test. The final model adequacy was tested by using the Cox-Snell residual test and the baseline distribution of the cumulative hazard function of residuals against Cox-Snell residuals was follows 45 degree indicating adequately fitted data. Adjusted hazard ratio (AHR) with a 95% confidence interval (CI) was estimated by multivariable Cox regression and a p-value less than or equal to 0.05 was used to declare the presence of statistically significant association between recovery time and explanatory variable.

RESULTS

Socio-demographic and treatment-related characteristics of study population

The study included records of children 2-59 months of age admitted to the pediatric ward of Jimma University Medical Center (JUMC) with a diagnosis of severe pneumonia for five consecutive years (2018-2022). From the sample of 426, information was extracted from 394 patient charts and gives a response rate of 92.5%. The female-to-male ratio was 1:1.21 and 52.28% of the study participants came from the countryside. The median age of the study participants was 10 months (IQR: 5 - 21) and the majority (218: 55.33%) of them were under the age group of 2-11 months.

Almost two third (244: 62%) of the study participants were not enrolled in health insurance schemes.

About 47.72% of the children visited the hospital within three days of onset of illness. Danger signs were present in about (135: 34.26%) of the children at admission. The commonest danger signs were difficult breathing and severe chest in-drawing accounting for (30: 7.61%) and (25: 6.35%) respectively. Among 394 children (188: 47.72%) and (71: 18.02%) had grunting and convulsion respectively. Crystalline penicillin was given to (166: 42.35%) of the children at admission as a first-line treatment and antibiotics were changed for (128: 33%) of the children (Table 2).

Table 1: Frequency distribution of socio-demographic and treatment-related characteristics of children aged between 2-59 months with severe pneumonia admitted to the pediatric ward of Jimma University Medical Center (JUMC) from Jan 1, 2018-Dec 31, 2022(n=394)

Variable	Category	Status	
		Recovered (%)	Censored (%)
Age in month	2-11	196 (49.7)	22 (5.6)
	12-35	111 (28.2)	17 (4.3)
	36-59	42 (10.7)	6 (1.5)
Sex	Male	191 (48.5)	25 (6.3)
	Female	158 (40.1)	20 (5.1)
Danger signs	No	238 (60.4)	21 (5.3)
	Yes	111 (28.2)	24 (6.1)
Residence	Urban	170(43.16)	18(4.56)

	Rural	179(45.43)	27(6.85)
Antibiotics change	No<br>Yes	229 (59.0)<br>114 (29.4)	31 (7.99)<br>14 (3.61)
Antibiotic first given	Crystalline penicillin<br>Ampicillin and gentamicin<br>Ceftriaxone<br>Ceftazidime & vancomycin	152 (38.78)<br>77 (19.64)<br>109 (27.81)<br>9 (2.3)	14 (3.57)<br>12 (3.06)<br>16 (4.08)<br>3 (0.77)
Grunting	No<br>Yes	179 (45.4)<br>170 (43.19)	27 (6.85)<br>18 (4.56)

Vaccination, nutritional status, and co-morbidities of the study populations

Majority (281: 71.32%) of the children had a history of exclusive breastfeeding. Of the study participants, about (166: 42.13%) of the children were fully vaccinated whereas (60: 15.23%) were unvaccinated. Stunting, wasting, and being underweight were found in (143: 36.29%), (186: 47.21%), and (161: 40.86%) of the study participants respectively. About (82: 20.81%) of the children were born before the expected date of delivery. Almost (201:51%) of the children tested non-reactive for HIV and (6: 1.53%) of the children among the study participants were found reactive for the HIV test. About (98: 24.87%) of the children admitted with severe pneumonia had different co-morbidities. Severe acute malnutrition was one of the most common diseases in addition to severe pneumonia among the study participants. Other medical conditions like Bronchial asthma, Down syndrome, and pertussis accounted for 9 (2.29%) (Table 3)

Table 2: Frequency distribution of vaccination, nutritional status, and co-morbidities of children aged between 2-59 months with severe pneumonia admitted to the pediatric ward of Jimma University Medical Center (JUMC) from Jan 1, 2018-Dec 31, 2022(n=394)

Variable	Category	Status	
		Recovered (%)	Censored (%)
Vaccination status	Fully vaccinated	152 (38.58)	14 (3.55)
	Partially vaccinated	151 (38.32)	17 (4.33)
	Unvaccinated	46 (11.67)	14 (3.55)
Weight for height	Normal	183 (46.45)	25 (6.34)
	Wasted	166 (42.13)	20 (5.08)
Exclusive breastfeeding	No	99 (25.13)	14 (3.55)
	Yes	250 (63.45)	31 (7.87)
Weight for age	Normal	210 (53.29)	23 (5.84)
	Underweight	139 (35.27)	22 (5.58)
Height for age	Normal	219 (55.58)	32 (8.14)
	Stunted	130 (32.99)	13 (3.29)
Comorbidities	No	262 (66.5)	34 (8.6)
	Yes	87 (22.1)	11 (2.8)
HIV status	Reactive	5 (1.3)	1 (0.2)
	Non-reactive	187 (47.5)	14 (3.5)

	Unknown	157 (39.9)	30 (7.6)

Treatment Outcome and Incidence Rate of Recovery

Regarding the treatment outcome of severe pneumonia, the majority (349: 88.58%) of children recovered, (22: 5.58%) died, (14: 3.55%) were left against medical advice and (9: 2.28%) were discharged with unknown condition (Figure 2). The shortest and longest length of hospital stay was 1 and 29 days respectively. The total person-day (person time risk) contributed by the study participants was 2,211 days. The overall incidence rate recovery from severe pneumonia was 15.78 per 100-person day (95% CI, 14.2 – 17.5) observation.

The median time to recovery from severe pneumonia

The Kaplan-Meier survival curve was used to estimate the survival status of the children with severe pneumonia. According to the Kaplan-Meier survival estimation, the median survival time without using any stratification factors was 4 days (IQR: 3, 7). The graph shows a tendency to fall off quickly in the first five days, showing that most children recovered from severe pneumonia during this time period (Figure 3).

Kaplan Meier survival plot was performed to compare survival probability between categories of different predictors. Accordingly, survival probability among different categorical predictors such as co-morbidities, duration of time before seeking health care, weight for age, antibiotics first administered, and antibiotic change were different (See Figure 4).

Comparison of Median Survival Time

To compare the significance difference in median survival times of various categorical predictors, the log-rank test was used. Accordingly, there was a significant difference in median survival time between categorical predictors such as weight for age, height for age, co-morbidity, antibiotics first administered, and antibiotics change. For instance, the median recovery time of children without comorbidities was 4 days (IQR, 3 – 6) but for children with co-morbidities, it was 6 days (IQR, 4 – 11). Children who visited the Hospital within three days of onset of illness had a median recovery time of 4 days (IQR, 2 – 5) while those who were visited after seven days was 6 days (IQR, 4 – 13). For children for whom antibiotics were changed, the median recovery time was 5 days (IQR, 3 – 9) whereas for children for whom no antibiotics were changed, the median recovery time was 4 days (IQR, 3 – 6) but, there was no significant variation of median recovery time among categorical predictors like HIV status, residence, grunting, and age.

Proportional Hazard Assumption Test

The Cox proportional hazard assumption was checked statistically (Global test; p-value = 0.37 indicating the Cox proportional hazard assumption was not violated). Finally, Cox-Snell residual test was performed to check the final model adequacy and confirmed that the model is good to fit the data (Figure 5).

Predictors of time to recovery from severe pneumonia

After running univariate Cox regression, predictors such as sex, age, residence, grunting, antibiotic first administered, antibiotic change, vaccination status, height for age, weight for age, weight for height, comorbidities, and duration of time before seeking healthcare were selected as a candidate variable for multivariable Cox regression analysis. After performing multivariable Cox regression,

adjusted hazard ratios with a 95% confidence interval (CI) and a p-value of less than or equal to 0.05 were used to declare statistical significance. Thus, Variables such as presence of co-morbidities, duration of time before seeking health care, antibiotic first administered and antibiotics change were statistically significant predictors of time to recovery from severe pneumonia in this study.

The recovery time of children with comorbidity is delayed by 30% as compared to children without comorbidity (AHR; 0.70, (95% CI, (0.54 - 0.92)). Regarding the treatment regimen, the recovery time of children treated with ceftazidime and vancomycin as first-line antibiotics during admission was delayed by 71% as compared to children treated with crystalline penicillin (AHR; 0.29, (95% CI, 0.145 - 0.614)). Children for whom antibiotics were changed were 26% less likely to recover quickly than children for whom antibiotics were not changed (AHR; 0.74, (95% CI, 0.57 - 0 .94)). Children with severe pneumonia who came between 4 and 7 days to the Hospital had 27% slower recovery time as compared to children who came within 3 days (AHR; 0.73(95% CI, (0.561-0.959)) and those who came after 7 days of their illness to the Hospital had 42% slower recovery time as compared to the reference group (AHR; 0.58, (95% CI, 0.43 - 0.79)).

Table 3: Multivariable Cox regression analysis for independent predictors of recovery time among children aged 2-59 months with severe pneumonia admitted to the pediatric ward of Jimma University Medical Center (JUMC) from Jan 1, 2018- Dec 31, 2022

Variable	Category	p-value	CHR, 95%CI	AHR, 95%CI
Sex	Male		1	1
	Female	0.038	0.79(0.645-0.988)	0.92(0.727 - 1.155)
Residence	Urban		1	1

	Rural	0.225	0.87(0.711-1.083)	0.94 (0.752 - 1.175)
Age	2-11 months<br>12-35 months<br>36-59 months	 0.226<br>0.204	1<br>0.86(0.684-1.093)<br>0.80(0.576-1.124)	1<br>1.00(0.736 - 1.382)<br>1.05(0.678 - 1.636)
Grunting	No<br>Yes	 0.122	1<br>0.84(0.686-1.045)	1<br>0.85 (0 .680 - 1.071)
Antibiotics first administered	Cr. penicillin<br>Ampicillin & Gentamycin<br>Ceftriaxone<br>Ceftazidime & Vancomycin	 0.000<br>0.000<br>0.000	1<br>0.50(0.379-0.665)<br>0.55(0.432-0.714)<br>0.23(0.117-0.457)	1<br>**0.59(0.439- 0.798) ****<br>**0.56(0.426 - 0.727) ****<br>**0.29(0.143- 0.600) ****
Antibiotic change	No<br>Yes	 0.001	1<br>0.66(0.532-0.839)	1<br>**0.74(0.581 - 0.953) ***
Vaccination status	Fully vaccinated<br>Partially vaccinated<br>unvaccinated	 0.734<br>0.001	1<br>1.04(0.829-1.303)<br>0.57(0.411-0.799)	1<br>1.01 (0.728 - 1.402)<br>0.70(0.489 - 1.029)
Time prior to seeking health care	Up to 3 days<br>4 - 7days<br>Above 7 days	 0.003<br>0.000	1<br>0.67(0.525-0.872)<br>0.51(0.392-0.669)	1<br>**0.73 (0.561- 0.959) ***<br>**0.58 (0.432 - 0.789) ****
Comorbidities	No<br>Yes	 0.000	1<br>0.60(0.473-0.774)	1<br>**0.70 (0.543 - 0.914) ***

Height for age	Normal<br>Stunted	0.059	1<br>0.81(0.651-1.007)	1<br>0.85(0.671- 1.072)
Weight for height	Normal<br>Wasted	0.055	1<br>1.23(.995-1.528)	1<br>1.15(0.919 - 1.439)
Weight for age	Normal<br>Underweight	0.003	1<br>0.71(0.576-0.890)	1<br>0.89(0.699 - 1.134)

P-Value ≤0.05(*) and ** for p-value < 0.01

DISCUSSION

Children between the ages of 2-59 months with severe pneumonia who were admitted to the pediatric ward of Jimma University Medical Center (JUMC) were assessed for recovery time and its predictors. In the current study, the median recovery time from severe pneumonia was 4 days IQR (3, 7). Different predictors were found to contribute to the recovery time of children admitted with severe pneumonia in this study. Predictors like antibiotic first administered, antibiotic change, duration of time before seeking health care, and comorbidity had significant associations with recovery time.

According to the current study, among children who were admitted to the pediatric ward of Jimma University Medical Center between the ages of 2-59 months, the median recovery time from severe pneumonia was 4 days. This finding is in line with other studies conducted in Debre Markos [(29)], Hosanna [(8)] and Gambia. .[(12)] This finding is longer than the British Thoracic Society guideline [(30)] and the study conducted in Nepal that estimates the recovery time of 2 days.[(31)] The variation in the results may be explained by the difference in socioeconomic status of the research areas, unique

circumstances, such as health care setting, and the age of the study participants, as the study involved Nepalese children between the ages of 2-35 months.

The current finding is lower than a study conducted in Tanzania which estimated the median recovery time of 10 days. [32] The discrepancy could be a result of the time difference between study periods. The difference in the age of the study population could also explain the discrepancy, as the Tanzanian study was conducted among children aged between the ages of 0 and 144 months. The analysis approach (model) used, in the Tanzanian study was logistic regression, which does not take the censoring of study participants into account. The study design in Tanzanian study was a prospective cohort study which is better in generating strong evidence could explain the variability.

According to the study conducted in Poland, patients stay for an average of 8.2 to 10.1 days. [33] This variation might be due to high bed occupancy rate in Ethiopia and patients are discharged, as soon as their signs and symptoms subside without sending and waiting for any laboratory confirmation. On the contrary, Poland is high-income country compared to Ethiopia, and their health care system might be well organized and developed.[34] The bed occupancy rate in Poland is probably low and people can pay any charge at the hospital that may create an opportunity for patients to stay longer at the hospital until complete stability from all signs and symptoms of the disease.

Duration of time prior to seeking health care was an independent significant predictor of the recovery time of severe pneumonia. Children who came to the hospital within three days of illness recovered quicker than those children who came after seven days of illness. This finding is supported by a prospective follow-up study conducted in the Gambia [12] and other retrospective studies conducted in Debre Markos Hospital [29], Ayder Comprehensive Specialized Hospital [22],

and Central and North Gondar zones. [21] Lately presented cases of pneumonia might develop a complication that needs further investigation to treat and this condition in turn increases the length of time required to recover.[35]

The existence of co-morbidity was another significant predictor that was connected with the length of time needed to recover from severe pneumonia in this study. Those children with any type of co-morbidity were found to stay longer in the hospital than their counterparts who were free of comorbidities. This finding is consistent with other studies conducted at Debre Markos [29], Central and North Gondar zones [21], and Nepal. [31] The reason why children with co-morbidity stay for a prolonged time at the hospital might be due to the fact that Co-morbidities significantly increase the severity of pneumonia, including the need for oxygen, and are significantly associated with more complications, that increases the recovery time.[32] Since the major comorbidity in the current study was severe acute malnutrition, as adequate protein and energy are needed for proper immune system functioning; undernourished children have a compromised immune system and are more vulnerable to developing serious infections and complications.[36]

The antibiotic first administered was one of the significant clinical and treatment-related predictors in this study. Children with severe pneumonia who were treated with ceftriaxone and ampicillin and gentamicin had a prolonged recovery time. This might be due to ampicillin and gentamicin being administered together for patients presented with severe acute malnutrition in addition to severe pneumonia.[37] Children with severe pneumonia who were treated with ceftazidime and vancomycin as the first antibiotic at the time of admission stayed longer in the hospital. This might be because ceftazidime and vancomycin together administered for children with comorbidity, particularly for severe bacterial infections like endocarditis and invasive methicillin- resistance staphylococcus aureus infections.[38, 39]

Antibiotic change is also a significant predictor of recovery time. Compared to children whose medications were not altered, those whose antibiotics were changed required longer hospital stays to recover from severe pneumonia. Antibiotics are altered whenever there is treatment failure on first-line drugs due to drug resistance or selection of inappropriate drug to treat the patients.[40] For this reason, the additional time required for second-line drugs to act could explain the longer recovery time. This finding is supported by other study conducted in Hosanna.[8]

Limitations of the Study

Although this study uses five years of data, important variables like parent's income(Wealth index) and educational status were not included. The study also didn't take any laboratory investigation into account such as baseline complete blood count, blood culture, and electrolyte sedimentation rate.

CONCLUSION

This study assessed the recovery time from severe pneumonia and its predictors among children aged 2-59 months admitted to the pediatric ward of Jimma University Medical Center (JUMC). The finding of this study revealed that the median recovery time was longer than other similar studies. Late presentation to hospital, antibiotic first administered, antibiotic change and presence of co-morbidities were significant predictors that prolonged recovery time. Healthcare providers should have to give due attention to the identified predictors of recovery time. Measures that help children to recover quickly from their illness should be strengthened.

ABBREVIATIONS

AHR: Adjusted Hazard Ratio; **BSc:** Bachelor of Science; **CEO:** Chief Executive Officer; **CI:** Confidence Interval; **CHR**: Crude Hazard Ratio; **FMOHE:** Federal Ministry of Health of

Ethiopia; **HIV:** Human Immune Virus; **Hib:** Haemophilus influenzae type b; **ICCM:** Integrated Community Case Management**; IMNCI:** Integrated Management of Newborn and Childhood Illness**; IQR:** Interquartile range**; JUMC:** Jimma University Medical Center**; MRN:** Medical Registration Number**; ORS:** Oral Rehydration Solution**; PCV:** Pneumococcal Conjugate Vaccine**; SAM:** Severe Acute Malnutrition**; SD:** Standard Deviation**; SSA:** Sub-Saharan Africa**; TB:** Tuberculosis**; WHO:** World Health Organization

Ethical Considerations

Prior to data collection, this research protocol has been submitted to the Institutional Review Board (IRB) of Jimma University and meets the ethical and scientific standards outlined in national and international guidelines. Jimma University, Institute of Health (Reference No JUIH/IRB/361/23) approved the consent wavier. Then a letter of permission was written to Jimma University Medical Center managers. Letter to access the patient's chart was obtained from the chief executive officer (CEO) of Jimma University Medical Center and the health management information system focal person verbally consented to use the patients' medical chart for data collection. The study was conducted following the relevant international guidelines, regulations, and principles of Helsinki declaration. Verbal or written consent was not obtained from the study participants due to the fact that the data were collected from patients' medical charts and there was no direct contact between patients and data collectors. Throughout the study, the information obtained from the patient's medical records was kept entirely confidential and only accessed by the research team, and solely for the purpose of the research. For data collection purposes, medical record number were utilized, and no personal identifiers were gathered or utilized in the research report.

Acknowledgments

The authors would like to acknowledge Jimma University Ethical Review Board for providing the ethical clearance letter and Shenen Gibe General Hospital for their valuable and preliminary support. We also extend our heart full gratitude to all the data collectors and supervisors for their efforts in the data collection and chart review process.

Author contributions

Getu Girma Bekele: Conceptualizations, Data curation, Formal analysis, Investigations, Methodology, Supervision, Validation, Visualization, writing an original draft, and Writing Review and editing the manuscript.

Masrie Getnet: Formal analysis, Investigations, Methodology, Supervision, Visualization, Writing Review and editing the manuscript.

Dawit Regassa: Formal analysis, Investigations, Methodology, Supervision, Visualization, Writing Review and editing the manuscript.

Tola Getachew: Formal analysis, Investigations, Methodology, Supervision, Visualization, Writing Review and editing the manuscript.

Funding

No funds were obtained for this particular study.

Disclosure

No conflict of interest in this particular study.

References

1. McAllister DA, Liu L, Shi T, Chu Y, Reed C, Burrows J, et al. Global, regional, and national estimates of pneumonia morbidity and mortality in children younger than 5 years between 2000 and 2015: a systematic analysis. Lancet Glob Health. 2019;7(1):e47-e57.

2. Nair1 IRH, KLOB, Li Liu2, E, Lukšić4 T, SQ. Epidemiology and etiology of childhood pneumonia in 2010: estimates of incidence, severe morbidity, mortality, underlying risk factors and causative pathogens for 192 countries.

3. UNICEF. A fair chance for every child 2016 [Available from: https://www.unicef.org/serbia/media/1216/file/State%20of%20the%20world%27s%20children%202016.pdf.

4. Pneumonia in children [Available from: https://www.who.int/news-room/fact-sheets/detail/pneumonia.

5. Naheed A, Breiman RF, Islam MS, Saha SK, Tabassum Naved R. Disparities by sex in care-seeking behaviors and treatment outcomes for pneumonia among children admitted to hospitals in Bangladesh. PLoS One. 2019;14(3):e0213238.

6. Helle Ostergaard Madsen MH, Ashima Rani Das, Prabhakar D Moses, Winsley Rose, Mammen Puliyel, Flemming Konradsen, K R John, Anuradha Bose. Costing of severe pneumonia in hospitalized infants and children aged 2-36 months, at a secondary and tertiary level hospital of a not-for-profit organization. 2019.

7. Hauck K ZX. How Dangerous is a Day in Hospital?: A Model of Adverse Events and Length of Stay for Medical Inpatients. Medical Care. 2011.

8. Tirore LL, Abame DE, Sedoro T, Ermias D, Arega A, Tadesse T, et al. Time to Recovery from Severe Pneumonia and Its Predictors Among Children 2-59 Months of Age Admitted to

Pediatric Ward of Nigist Eleni Mohammed Memorial Comprehensive Specialized Hospital, Hossana, Ethiopia: Retrospective Cohort Study. Pediatric Health Med Ther. 2021;12:347-57.

9. Mulholland E, Smith L, Carneiro I, Becher H, Lehmann D. Equity and child-survival strategies. Bull World Health Organ. 2008;86(5):399-407.

10. Zhang S, Sammon PM, King I, Andrade AL, Toscano CM, Araujo SN, et al. Cost of management of severe pneumonia in young children: systematic analysis. Journal of Global Health. 2016;6(1).

11. Kamolz L-P, Marfil-Garza BA, Belaunzarán-Zamudio PF, Gulias-Herrero A, Zuñiga AC, Caro-Vega Y, et al. Risk factors associated with prolonged hospital length-of-stay: 18-year retrospective study of hospitalizations in a tertiary healthcare center in Mexico. Plos One. 2018;13(11).

12. Kuti BP, Adegoke SA, Oyelami OA, Ota MO. Predictors of prolonged hospitalisation in childhood pneumonia in a rural health centre. South African Journal of Child Health. 2014;8(1).

13. Black RE, Morris SS, Bryce J. Where and why are 10 million children dying every year? Lancet. 2003;361(9376):2226-34.

14. WHO. Revised WHO classification and treatment of childhood pneumonia at health facilities.

15. Ministry of Health, Federal Democratic Republic of Ethiopia (2015). Ethiopia: Health Sector Transformation Plan 2015/16 – 2019/20

(2008-2012 EFY). [Available from: https://ephi.gov.et/wp-content/uploads/2023/01/2.-Ethiopia-Health-Sector-Transformation-Plan-I-2015-2020-Endline-Review.pdf.

16. Alamneh YM, Adane F. Magnitude and Predictors of Pneumonia among Under-Five Children in Ethiopia: A Systematic Review and Meta-Analysis. J Environ Public Health. 2020;2020:1606783.

17. Tegenu K, Geleto G, Tilahun D, Bayana E, Bereke B. Severe pneumonia: Treatment outcome and its determinant factors among under-five patients, Jimma, Ethiopia. SAGE Open Med. 2022;10:20503121221078445.

18. Jimma University Medical Center (JUMC) [Available from: https://www.lucaincroccifoundation.org/JUMC.

19. Jimma university medical center health managment information system. 2023.

20. Schoenfeld DA. Sample size formula for the proportional hazards regression model.

21. Tamirat ZG, Gelagay AA, Boke MM. Time to recovery and its predictors among children age of 2 up to 59 months admitted with severe community acquired pneumonia in Public Hospitals of Central and North Gondar Zones, Ethiopia 2021. 2022.

22. Amare RA, Fisseha G, Berhe A, Tirore LL. Incidence of recovery from severe pneumonia and its predictors among children 2-59 months admitted to pediatric ward of Ayder Comprehensive Specialized Hospital, Tigray, Ethiopia: A retrospective Cohort study. J Family Med Prim Care. 2022;11(9):5285-92.

23. what-is-pneumonia [Available from: https://www.thoracic.org/patients/patient-resources/resources/what-is-pneumonia.pdf.

24. Ndu IK, Ekwochi U, Osuorah CD, Onah KS, Obuoha E, Odetunde OI, et al. Danger Signs of Childhood Pneumonia: Caregiver Awareness and Care Seeking Behavior in a Developing Country. Int J Pediatr. 2015;2015:167261.

25. Ayalew T. Exclusive breastfeeding practice and associated factors among first-time mothers in Bahir Dar city, North West Ethiopia, removed: A community based cross sectional study. Heliyon. 2020;6(9):e04732.

26. Tiruneh CM, Emiru TD, Tibebu NS, Abate MW, Nigat AB, Bantie B, et al. Underweight and its associated factors among pediatrics attending HIV Care in South Gondar Zone public health facilities, Northwest Ethiopia, 2021. BMC Pediatrics. 2022;22(1).

27. Luzingu JK, Stroupe N, Alaofe H, Jacobs E, Ernst K. Risk factors associated with under-five stunting, wasting, and underweight in four provinces of the Democratic Republic of Congo: analysis of the ASSP project baseline data. BMC Public Health. 2022;22(1).

28. Lee SW. Kaplan-Meier and Cox proportional hazards regression in survival analysis: statistical standard and guideline of Life Cycle Committee. Life Cycle. 2023;3.

29. Patman S, Mengist B, Tesfa M, Kassie B. Time to recovery and predictors of severe community-acquired pneumonia among pediatric patients in Debre Markos referral hospital, North West Ethiopia: A retrospective follow-up study. Plos One. 2020;15(9).

30. Michael Harris JC, Nicky Coote, Penny Fletcher et al. British Thoracic Society Guidelines for the Management of Community Acquired Pneumonia in Children: Update 2011.

31. Thorne C, Basnet S, Sharma A, Mathisen M, Shrestha PS, Ghimire RK, et al. Predictors of Duration and Treatment Failure of Severe Pneumonia in Hospitalized Young Nepalese Children. Plos One. 2015;10(3).

32. Caggiano S, Ullmann N, De Vitis E, Trivelli M, Mariani C, Podagrosi M, et al. Factors That Negatively Affect the Prognosis of Pediatric Community-Acquired Pneumonia in District Hospital in Tanzania. Int J Mol Sci. 2017;18(3).

33. Gajewska M, Lewtak K, Scheres J, Albrecht P, Gorynski P. Trends in Hospitalization of Children with Bacterial Pneumonia in Poland. Cent Eur J Public Health. 2016;24(3):188-92.

34. Smarzewska D, Wereda WS, Jonczyk JA. Assessment of the Health Care System in Poland and Other OECD Countries Using the Hellwig Method. Int J Environ Res Public Health. 2022;19(24).

35. MacKian S. A review of health seeking behaviour: problems and prospects.

36. Kidane GF, Zereabruk K, Aberhe W, Hailay A, Mebrahtom G, Gebremeskel GG, et al. Time to recovery from severe acute malnutrition and its predictors among under five children admitted to therapeutic feeding units of general and referral hospitals in Tigray, Ethiopia, 2020: a prospective cohort study. BMC Pediatr. 2023;23(1):325.

37. UNICEF. Mangment of severe acute malnutrition in children: working towards resultsat sale: 2015.

38. Matsumoto K, Takesue Y, Ohmagari N, Mochizuki T, Mikamo H, Seki M, et al. Practice guidelines for therapeutic drug monitoring of vancomycin: a consensus review of the Japanese Society of Chemotherapy and the Japanese Society of Therapeutic Drug Monitoring. J Infect Chemother. 2013;19(3):365-80.

39. Frymoyer A, Hersh AL, Benet LZ, Guglielmo BJ. Current recommended dosing of vancomycin for children with invasive methicillin-resistant Staphylococcus aureus infections is inadequate. Pediatr Infect Dis J. 2009;28(5):398-402.

40. Berkley JA, Muro RP, Masoza TS, Kasanga G, Kayange N, Kidenya BR. Predictors and outcome of first line treatment failure among under-five children with community acquired severe pneumonia at Bugando Medical Centre, Mwanza, Tanzania: A prospective cohort study. Plos One. 2020;15(12).

Figure Click here to access/download;Figure;Figure 1.docx

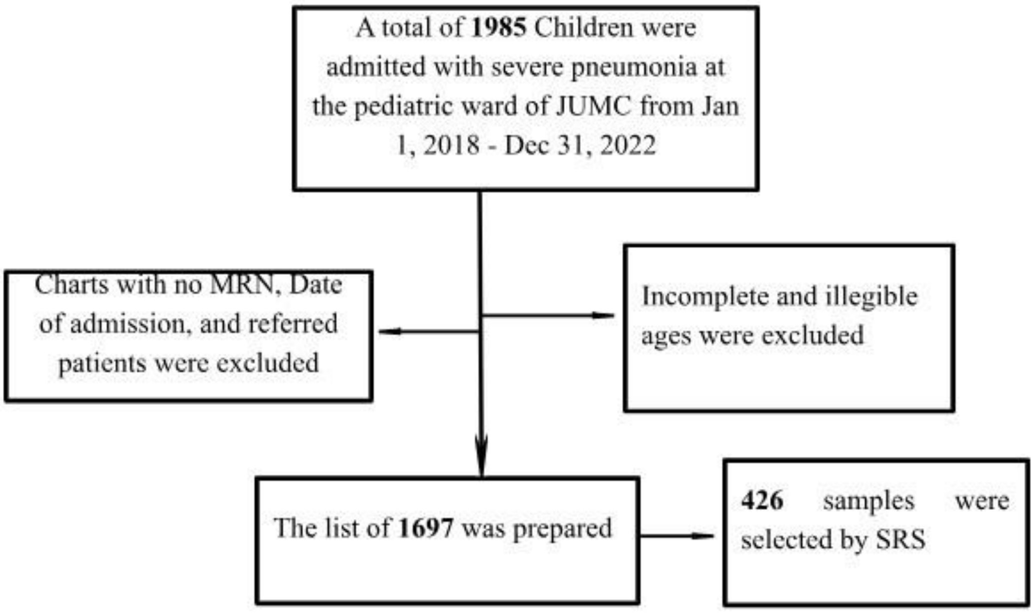

Figure 1: A schematic representation of the sampling procedure for selecting the study population to evaluate the duration of recovery from severe pneumonia and its predictors among 2-59 months of age admitted to the pediatric ward of JUMC.

Click here to access/download;Figure;Figure 2.docx

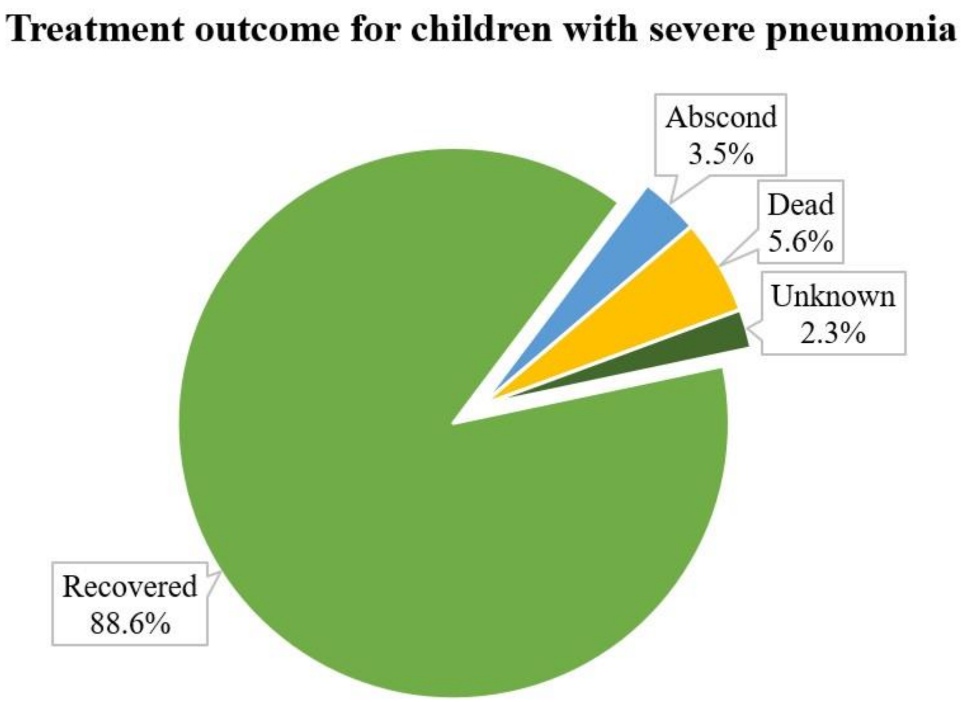

Figure 1: Shows the results of the treatment outcome for children with severe pneumonia who were hospitalized to the pediatric ward of the Jimma University Medical Center between January 1, 2018, and December 31, 2022 (n=394).

Figure Click here to access/download;Figure;Figure 3.docx

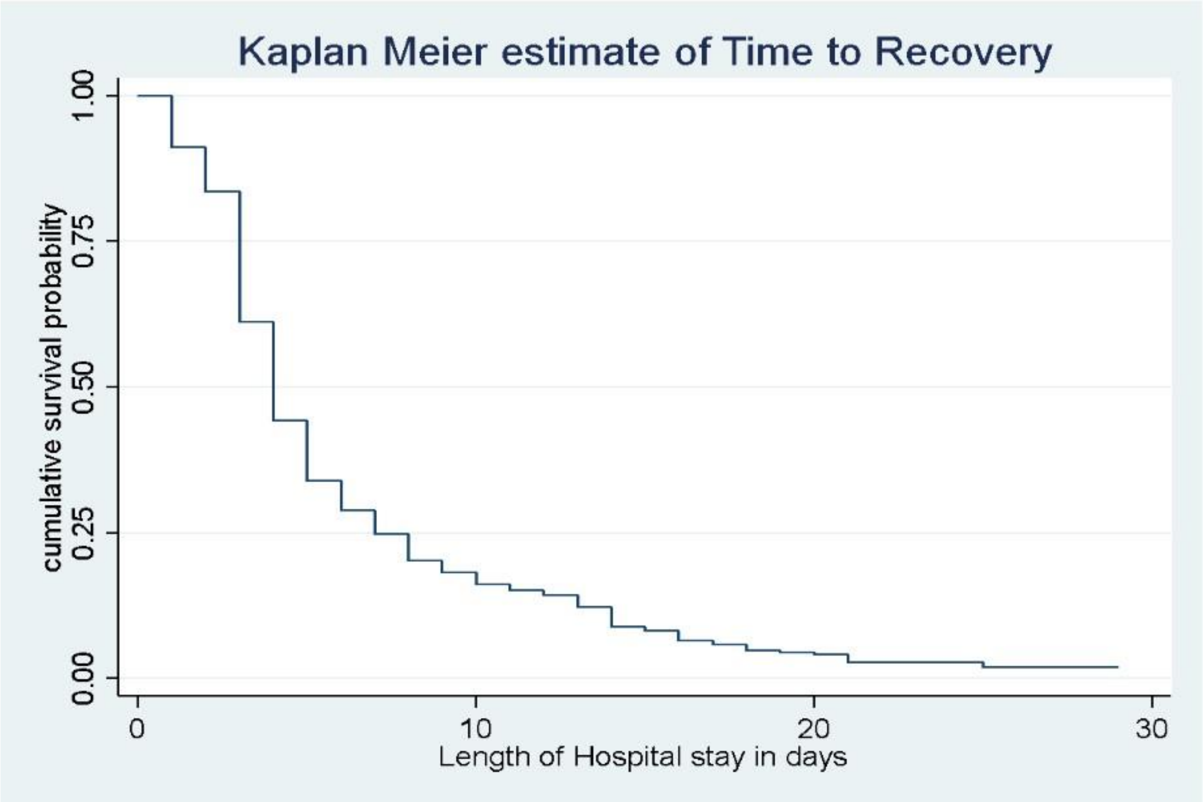

Figure 1: The Kaplan-Meier survival distribution of child aged 2-59 months with severe pneumonia admitted to pediatric ward of JUMC from Jan 1, 2018-Dec 31, 2022(n=394).

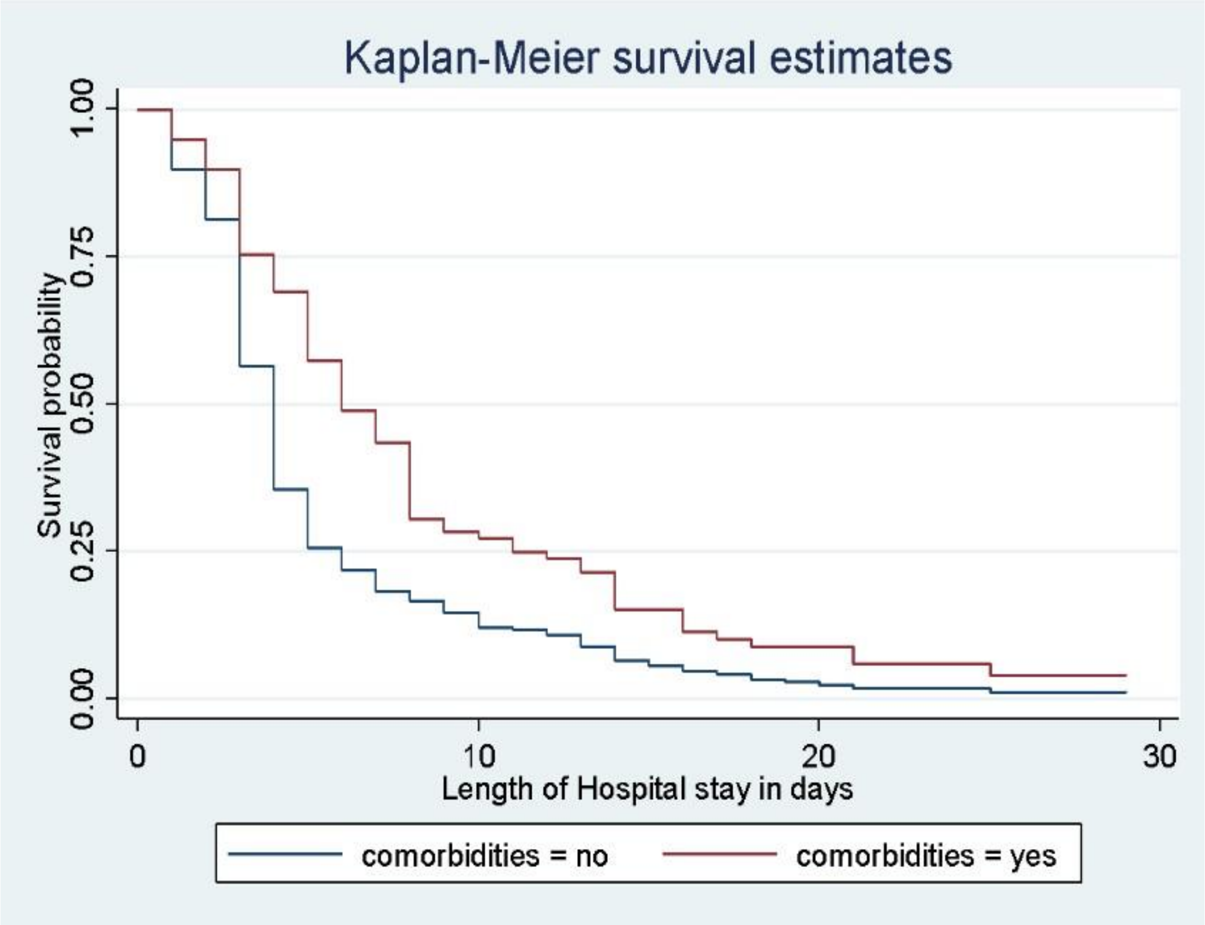

Figure 1: The cumulative survival function by comorbidity among children aged 2-59 months with severe pneumonia admitted to the pediatric ward of JUMC from Jan 1, 2018-Dec 31, 2022(n=394).

Figure Click here to access/download;Figure;Figure 5.docx

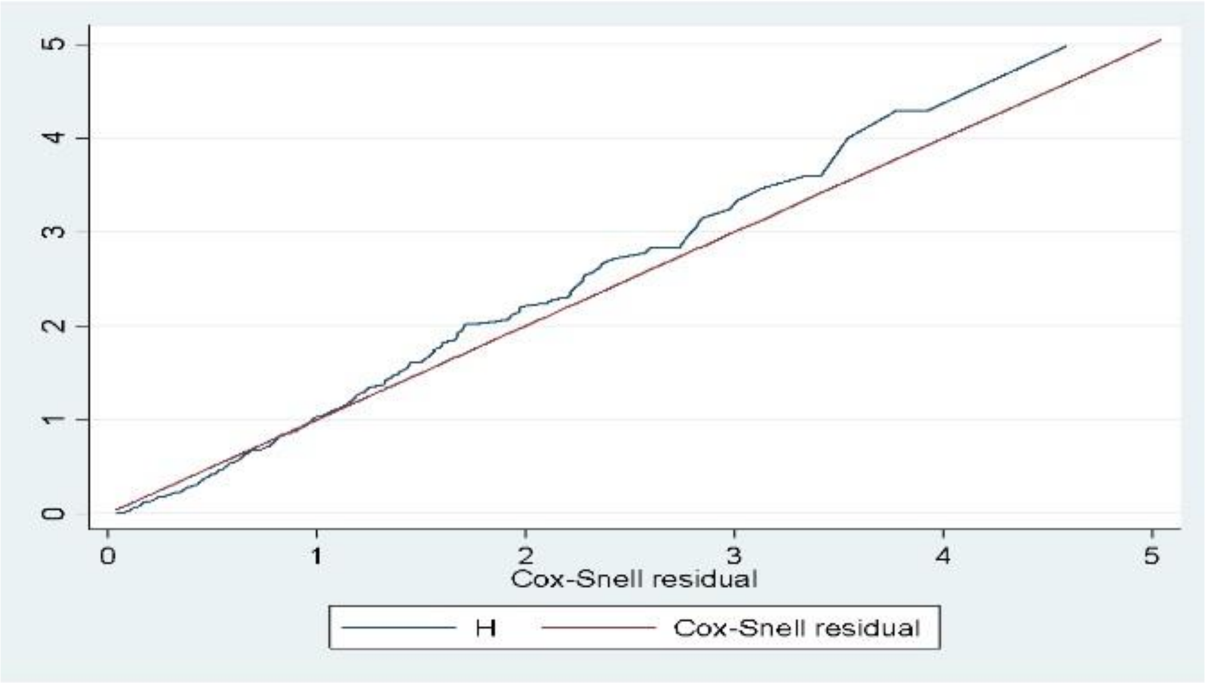

Figure 1: A cumulative hazard plot of cox snell residuals for model fitness

Revised manuscript with track changes

Time-to-Recovery from Severe Pneumonia and iIts Predictors aAmong Children 2-59 Months of Age Admitted to Pediatric Ward of Jimma University Medical Center, Southwest, Ethiopia, 2023: A Retrospective Cohort Study; 2018-2022.

Getu Girma Bekele[1*], Masrie Getnet[2], Dawit Regassa[2], Tola Getachew Bekele[1]

Authors' Affiliation

[1] Jimma University Medical Center, Jimma, Ethiopia

[2]Jimma University, Institute of Health, Faculty of Public Health, Department of Epidemiology, Jimma, Ethiopia

Authors' Email Addresses

Getu Girma Bekele: Email:getgirma2013@gmail.com, ORCID: https://orcid.org/0009-0003-2864-4414, Jimma, Ethiopia

Masrie Getnet: masriegetnet16biostat@gmail.com, Jimma, Ethiopia

Dawit Regassa: dawitregassa4@gmail.com, Jimma, Ethiopia

Tola Getachew Bekele: tolagetachew613@gmail.com, ORCID: https://orcid.org/0000-0003-1374-0166, Jimma, Ethiopia

Corresponding Author: Getu Girma Bekele **Email:** getgirma2013@gmail.com

Phone: +251937640136

Abstract

Background: Pneumonia is an inflammation of ~~the~~ lung parenchyma. The World Health Organization estimated~~s~~ ~~that~~ 156 million cases of pneumonia occur annually. Out of them~~,~~, ~~including up to~~ 20 million cases severe enough to require hospitalization, and each year 1.2 million deaths occur~~each year~~ among under-five children. Despite studies and initiatives aimed at reducing pneumonia related deaths in children, Ethiopia is ranked sixth among top fifteen countries in terms of pneumonia related morbidity and mortality.

Objectives: This study aimed to assess the time to recovery from severe pneumonia and its predictors among children aged 2-59 months admitted to the pediatric ward of Jimma University Medical Center; Southwest, Ethiopia, 2023.

Methods: A facility-based retrospective cohort study was carried out among 426 children aged between 2 and 59 months. Five years of medical records, from 2018 – 2022, were reviewed. A simple random sampling technique was used. Data entry was done in~~using~~ epidata version 4.6 and exported to and analyzed by STATA version 15. Variables with p-value < 0.25 at Bivariable Cox regression analysis were selected for the multivariable Cox proportional model ~~Kaplan Meier's plot was used to compare the survival probability of different groups.~~ ~~and~~ A multivariable Cox regression model with 95% confidence interval and Adjusted Hazard Ratio was used to identify a significant predictor of time to recovery at a p-value < 0.05.~~the multivariable Cox regression model was used to investigate the independent effect of covariates on recovery time.~~

Result: The median recovery time was 4 days (IQR: 3, 7). Incidence rate of recovery was 15.78 per 100-person day (95% CI 14.2 – 17.5). The presence of co-morbidity (AHR; 0.7, 95% CI (0.54 – 0.91)), being treated with ceftazidime and vancomycin (AHR; 0.29, 95% CI (0.14 – 0.60)), antibiotic change (AHR; 0.74, 95% CI (0.58 – 0.95)) and late presentation to the Hospital (AHR; 0.58, 95% CI (0.43 -0.78)) were statistically significant predictors that prolong recovery time.

Conclusion: The median recovery time was longer than other similar studies. Therefore, due attention should be given to ~~concerning~~ the identified predictors of the recovery time.

Keywords: Time to recovery~~Recovery~~, Severe Pneumonia, predictors, Children 2-59 Months, Jimma.

INTRODUCTION

The World Health Organization estimated~~s~~ that 156 million cases of pneumonia occur annually in children under~~ the~~ age of five, including up to 20 million cases severe enough to require hospitalization, and 1.2 million deaths each year.[1] The greatest incidence was occurring in south Asia (2,500cases per 100,000 children) and west and central Africa (1,620 cases per 100,000 children).[2] Approximately 490,000 children under the age of five died of pneumonia in Sub Saharan Africa (SSA) in the year 2016.[3] In Ethiopia, approximately 3,370,000 children contract pneumonia each year which~~ and~~ accounts for 14% of all deaths of children under five years old. [4]

Severe pneumonia is the major cause of hospitalization in under-five children.~~,~~ A~~a~~mong 156,847 admitted children identified in seven hospitals in Bangladesh,~~;~~ pneumonia was the most diagnosed (17.9%).[5] Hospitalization alone was found to contribute to 46.8% of a household price for one severe pneumonia episode.[6] Effective management of pneumonia is not just only the ratio of the children recovered, but also the time of recovery. Spending each night in the hospital increases the

risk of developing adverse medication reactions, ulcers, and infections by 0.5%, 0.5%, and 1.6%, respectively.[7] Tremendous independent clinical factors marked up mortality and duration of stay associated with pneumonia, with significant variability among hospitals. Some studies identify different forms of malnutrition, co-morbidities, diarrhea, antibiotics first administered, being underweight, and age are the major factors that affect recovery time.[8]

A longer hospital stay has a significant negative impact on the admitted child, his or her parents, and the healthcare system, particularly in societies with resource-limited settings and no health insurance scheme.[9] Between 26.6% and 115.8% of monthly household income is spent on the direct medical costs of treating severe pneumonia in a hospital setting. Non-medical costs (such as travel and food) during a child's hospital stay for severe pneumonia account for 9.0% and 31.0% of the household's monthly income in Kenya and Guinea, respectively.[10] Patients with prolonged length of stay extremely account for the consumption of more hospital resources, Hospital acquired infection, and denies critically ill patients' timely access to treatment and contributes to capacity shortages.[11] Hence, these children are often discharged against medical advice without receiving full therapy, which results in an increased rate of readmission and complications such as pneumonic effusion, empyema, necrotizing pneumonia, and lung abscess.[12]

The Federal Ministry of Health of Ethiopia (FMOHE) has been working to prevent and reduce under-five mortality by introducing Pneumococcal Conjugate Vaccine (PCV) and Haemophilus influenzae type b (Hib) vaccines. The ministry is also implementing key ~~, as well as by putting into practice~~ strategies such as integrated community case management (ICCM) to train, support, and supply community health workers to enhance~~provide~~ diagnostic, treatment and referral services and integrated management of newborn and child illnesses (IMNCI) to improve case management skills of health care providers, improving health systems to provide quality care and

improving family and community health practice for health, growth, and development.[13, 14] Ethiopian Health Sector Transformation Plan in 2015, to reduce the mortality rate of children to 31 and 41 per 1000 live births in 2025 and 2035 respectively.[15] Despite these ~~those mentioned~~ program intervention, pneumonia remain the major causes~~s~~ and ~~endeavors, still the main causes~~ of illness and death in children under ~~the~~ age of five in Ethiopia ~~is pneumonia~~; with an approximated contribution of around 40,000 fatalities per year.[16]

Formatted: Superscript

P~~The reason the researcher is interested in this study is that p~~neumonia is one of the major public health importances. D~~and d~~espite the intervention that has been implemented in Ethiopia, ~~;~~ pneumonia continues as one of the main reasons of hospitalization and mortality among under-five children.[9] The widespread nature of the problem in Ethiopia has already killed thousands of children hospitalized with severe pneumonia, which requires everlasting solutions.[17] Understanding the average length of stay in hospitals ~~the recovery time~~ helps the health managers in evaluating the quality of health care delivered by a~~the~~ given health institution because the average length of stay indicates~~provides~~ information about ~~the~~ efficiency of care service delivery, and the standard of care being provided, and ~~the~~ effective use of resources. Furthermore, it can also provide valuable information to physicians and other healthcare providers to~~for~~ counsel~~ing~~ parents or caregivers about the duration of hospitalization so that the caregivers can timely mobilize resources for a child's hospital care.

To ~~Up to~~ the best of our knowledge and as far as our literature search is concerned, there are limited studies and evidence on ~~the~~ recovery time from severe pneumonia and its predictors in the current study area. As a result, the aim of this study was to estimate the median time of recovery from severe pneumonia and its predictors in children aged 2-59 months admitted to the pediatric ward of Jimma University Medical Center, Southwest, Ethiopia, 2023.

MATERIALS AND METHODS AND MATERIALS

Study Aarea and Period

The study was conducted at Jimma University Medical Center (JUMC). It is the only teaching and tertiary federal hospital found in southwest Ethiopia. Geographically, it is located in Jimma town, Oromia region, 353 km southwest of Addis Ababa, the capital city of Ethiopia. It was established in 1930 E.C. by Italian invaders for the service of their soldiers. Currently, JUMC provides services for approximately 18,289 inpatients on 800 beds, 232,000 outpatient attendants, 79,000 emergency service cases, and 6,500 deliveries in a year coming to the hospital from the catchment area. JUMC serves as a referral hospital in southwest Ethiopia. The population in the catchment area is estimated to be 15 million.[18, 19]

The pediatric department of JUMC has a pediatric outpatient, pediatric ward, and pediatric intensive care unit. The pediatric ward has three units namely level I, level II, and nutritional rehabilitation unit (NRU) with a total of 78 beds, and provides services for an average of 55-65 patients including severe pneumonia. On average over 400 children were diagnosed with severe pneumonia each year in this hospital. Children diagnosed with severe pneumonia were directly admitted to level I and treated by senior Pediatricians, Residents, Nurses, and Medical Interns. The pediatricantic ward has a total of 60 health professionals with 35 nurses, 7 senior physicians, and 16 residents. There are also pharmacy professionals and laboratory technologists who were working there by rotation.[19] The five-year data were reviewed from January 1, 2018, to December 31, 2022. The patient charts were retrieved from May 14- June 15, 2023.

Study Ddesign

A facility-based retrospective cohort study was conducted.

Population

Source population

All children 2 to 59 months of age who were admitted to the pediatric ward of Jimma University Medical Center (JUMC) with severe pneumonia were the target population.

Study population

All children with severe pneumonia aged 2-59 months who were managed at the pediatric ward of Jimma University Medical Center (JUMC) from January 1, 2018- December 31, 2022 were the study population.

Eligibility Criteria

Inclusion cCriteria

All children with severe pneumonia aged 2-59 months who were admitted to the pediatric ward of Jimma University Medical Center (JUMC) from January 1, 2018- December 31, 2022, were included.

Exclusion cCriteria

Children's charts with no Medical Registration Number (MRN), admission date, discharge date, and no or ambiguous age were excluded.

Sample size determination

The sample size was calculated by using the Schoenfeld formula for survival analysis by taking the following assumptions into account.[20]

Formatted: Superscript

- Significance level (α) (two-sided) =0.05, $Z_{\alpha/2} = 1.96$
- Power = 80%,$\beta = 0.2$ $Z_{\beta=}$0.842
- AHR = Adjusted hazard ratio was used from different similar studies.

- E = number of events (recovery).
- Pr(E) = probability of recovery was used from different similar studies.
- Finally, a 10% non-response rate (NRR) for an incomplete chart was added.
- $E = (z\alpha + z\beta)2$ / (ln AHR)^2 P (1-P), n = E/Pr(E).[20]

The final largest sample size **426** was considered to conduct the study.

Sampling Technique and Procedure

Sampling tTechnique

A simple random sampling technique was used to select the study populations.

Sampling pProcedure

The registry logbook of all children who were admitted with severe pneumonia to the pediatric ward of Jimma University Medical Center (JUMC) from January 1, 2018 – December 31, 2022, was reviewed. Since there was no case-specific registration log book, the list of all children aged 2–59 months admitted with severe pneumonia from January 1, 2018 – December 31, 2022, was prepared by excluding those with no MRN, admission date, discharge date, incomplete and ambiguous age. A total of 1985 children with severe pneumonia were noted from the registration logbook during the study period, of which 1697 completed charts were available. Then, 426 charts were sampled using a simple random sampling technique viaby a computer-generated method. (Figure 1).

Data collection tool and procedure

Data collection tool

The data werewas collected by using a structured checklist adapted from previous similar studies.[8, 12, 21, 22] The tool was designed based on the study objectives and contains socio-demographic

characteristics, clinical and treatment-related factors, vaccination and nutritional status as well as co-morbidities.

Data collection procedure

The unique medical record number of each patient was used to get their records (charts). All available information on the patient chart was checked. Then all the relevant variables were extracted from patient charts using an English version data extraction tool to meet the study objectives. Two skilled BSc nurses working in the pediatric ward of Jimma University Medical Center (JUMC) extracted the data from patient charts under the supervision of an a supervisor who is the MScC holder in pediatric and child health working in the pediatric ward of JUMC as a Head Nurse. Training was given to data extractors and supervisor regarding the significance of the study and ways of the data extraction process by the principal investigator.

Study variables

Outcome variable

Time to recovery from severe pneumonia measured in days.

Independent variables

Socio-demographic characteristics

The socio-demographic characteristics include the age, sex of the children, residence, and health insurance scheme.

Clinical and treatment-related factors

Under clinical and treatment-related factors Presence of Danger signs at admission, the duration time before seeking health care, Grunting, Head nodding, HIV status, Antibiotics first administered, Antibiotic change, and Convulsion were used.

Nutrition and Vaccination status

Nutrition and Vaccination status include Vaccination status, height for age, Weight for height, weight for age, Exclusive breastfeeding, Inability to feed, and different co-morbidities.

Operational definition

Severe Pneumonia: children presented with inability to drink, persistent vomiting, oxygen saturation less than 90%, convulsion, and lethargy or reduced level of consciousness.[23]

Time to Recovery: The time from admission to when the child is discharged from the hospital due to improvement in symptoms calculated by number of days.

Median Recovery Time: the time in which half (50%) of the children had recovered.

Censored: A child with severe pneumonia who has been admitted to the ward and happens to be dead, self-discharged, or with unknown outcome status.

~~**Survival Time:** is the time starting from the date of admission to recovery from severe pneumonia.~~

Comorbidity: Any disease acute or chronic present during admission in addition to severe pneumonia.

Danger signs: Children with difficult~~y~~ breathing, chest in drawing, oxygen saturation less than 90%, unconsciousness.[24]

Vaccination Status: Fully Vaccinated which refers to Children who had received all forms of vaccination; Partially Vaccinated (Children who had taken at least one PCV dose) and Unvaccinated (Children who have never received PCV or any form of vaccination).

Discharge status: indicate the condition (reason) for the patient’s termination from the program.

Exclusive Breastfeeding: Children who were introduced to only breast milk except for drugs, ORS (Oral Rehydration Solution), during the first six months of age.[25]

Underweight: Children having weight for age is less than -2SD (standard deviation).[26]

Wasting: Children having weight for height is less than -2SD (standard deviation).[27]

Stunting: Children having height for age is less than -2SD (standard deviation).[27]

Data Management

Data Quality Assurance

Data collectors were given orientation on the content of the data extraction tool and procedure. Two skillful BSc nurses working in the pediatric ward of Jimma University Medical Center (JUMC) reviewed the records after they were given training by the principal investigator. Prior toBefore the -actual data collection, the data extraction checklist was tested on 21 medical charts at Shenen Gibe Hospital to ensure that the data extraction format is agreed with the objectivesneeds of the study. During the data collection process, regular supervision was performeddone by the supervisor to ensure the quality of the data. Throughout the data collection process, the primary investigator and the supervisor routinely examined the accuracy and completeness of the data collected. Data entry and cleaning were done daily and timely feedback was being providedcommunicated to the data collectors.

Data analysis procedure

The data were entered inusing epidata version 4.6 exported to and analyzed using STATA version 15. The data were coded and cleaned prior tobefore the analysis. The data were described in terms of central tendency (median) and dispersion (interquartile range) for continuous data and frequency distribution for categorical data and presented using graphs, tables, and text. A day was

used to calculate the recovery time. Kaplan Meier's plot was used to compare the survival experience of different groups of patients by using survival curves. The association between the independent variable and time to recovery from severe pneumonia was analyzed using the Cox proportional hazard regression ~~model~~. Log rank test was used to identify the presence of significant difference in median survival time between different groups.[28]

Formatted: Superscript

Formatted: Superscript

Univariate analysis was performed and predictors that have an association with the outcome variable at a p-value of 0.25 or less were entered into the multivariable Cox regression model. The presence of Multicollinearity was checked using the variance inflation factor (VIF). A Variance Inflation Factor value of <10 was used as a cutoff point for indicating the absence of multicollinearity. A Cox proportional hazard assumption was checked ~~for each predictor graphically using a log minus log plot of survival and~~ statistically using the global residual test. The final model adequacy was tested by using the Cox-Snell residual test and the baseline distribution of the cumulative hazard function of residuals against Cox-Snell residuals was follows 45 degree indicating adequately fitted data. Adjusted hazard ratio (AHR) with a 95% confidence interval (CI) was estimated by multivariable Cox regression and a p-value less than or equal to 0.05 was used to declare~~d~~ the presence of ~~a~~ statistically significant association between ~~the~~ recovery time and explanatory variable.

RESULTS

Socio-demographic and treatment-related characteristics of study populations

The study included records of children 2-59 months of age admitted to the pediatric ward of Jimma University Medical Center (JUMC) with a diagnosis of severe pneumonia for five consecutive years (2018-2022). From the sample~~s~~ of 426, information was extracted from 394 patient charts

and gives~~making~~ a ~~the~~ response rate of 92.5%. The female-to-male ratio was 1:1.21 and 52.28% of the study participants came from the countryside. The median age of the study participants was 10 months (IQR: ~~(~~5 - 21) and the majority (218: ~~(~~55.33%) of them were under the age group of 2-11 months. Almost two third (244: ~~(~~62%) of the study participants were not enrolled in health insurance schemes.

About 47.72% of the children visited the hospital within three days of onset of illness~~becoming ill~~. Danger signs were present in about (135: ~~(~~34.26%) of the children at admission. The commonest danger signs were difficult~~y~~ breathing and severe chest in-drawing accounting for (30: ~~(~~7.61%) and (25: ~~(~~6.35%) respectively. Among 394 children (188: ~~(~~47.72%) and (71: ~~(~~18.02%) had grunting and convulsion respectively. Crystalline penicillin was given to (166: ~~(~~42.35%) of the children at admission as a first-line treatment and antibiotics were changed for (128: ~~(~~33%) of the children (Table 2).

Table 1: Frequency distribution of socio-demographic and treatment-related characteristics of children aged between 2-59 months with severe pneumonia admitted to the pediatric ward of Jimma University Medical Center (JUMC) from Jan 1, 2018-Dec 31, 2022(n=394)

Variable	Category	Status	
		Recovered (%)	Censored (%)
Age in month	2-11	196 (49.7)	22 (5.6~~5~~)
	12-35	111 (28.2)	17 (4.3)
	36-59	42 (10.7~~6~~)	6 (1.5)
Sex	Male	191 (48.5)	25 (6.3)
	Female	158 (40.1)	20 (5.1)

Danger signs	No<br>Yes	238 (60.4)<br>111 (28.2)	21 (5.3)<br>24 (6.1~~0~~)
Residence	Urban<br>Rural	170(43.16)~~18 (4.5)~~<br>179(45.43)~~27 (6.8)~~	18(4.56)~~170 (43.1)~~<br>27(6.85)~~179 (45.4)~~
Antibiotics change	No<br>Yes	229 (59.0)<br>114 (29.4)	31 (7.99~~8~~)<br>14 (3.61~~0~~)
Antibiotic first given	Crystalline penicillin<br>Ampicillin and gentamicin<br>Ceftriaxone<br>Ceftazidime & vancomycin	152 (38.78~~7~~)<br>77 (19.64)<br>109 (27.81~~0~~)<br>9 (2.3~~29~~)	14 (3.57)<br>12 (3.06)<br>16 (4.08)<br>3 (0.77~~6~~)
Grunting	No<br>Yes	179 (45.4)<br>170 (43.19)	27 (6.85)<br>18 (4.56)

Vaccination, nutritional status, and co-morbidities of the study populations

Majority~~ost~~ (281: ~~(~~71.32%) of the children had a history of exclusive breastfeeding. Of the study participants, about (166: ~~(~~42.13%) of the children were fully vaccinated whereas (60: ~~(~~15.23%) were unvaccinated. Stunting, wasting, and being underweight were found in (143: ~~(~~36.29%), (186: ~~(~~47.21%), and (161: ~~(~~40.86%) of the study participants respectively. About (82: ~~(~~20.81%) of the children were born before the expected date of delivery. Almost (201:~~(~~51%) of the children tested non-reactive for HIV and (6: ~~(~~1.53%) of the children among the study participants were found

reactive for the HIV test. About (98: (24.87%) of the children admitted with severe pneumonia had different co-morbidities. Severe acute malnutrition was one of the most common diseases in addition to severe pneumonia among the study participants. Other medical conditions like Bronchial asthma, Down syndrome, and pertussis accounted for 9 (2.29%) (Table 3)

Table 2: Frequency distribution of vaccination, nutritional status, and co-morbidities of children aged between 2-59 months with severe pneumonia admitted to the pediatric ward of Jimma University Medical Center (JUMC) from Jan 1, 2018-Dec 31, 2022(n=394)

Variable	Category	Status	
		Recovered (%)	Censored (%)
Vaccination status	Fully vaccinated	152 (38.587)	14 (3.55)
	Partially vaccinated	151 (38.32)	17 (4.331)
	Unvaccinated	46 (11.67)	14 (3.55)
Weight for height	Normal	183 (46.454)	25 (6.34)
	Wasted	166 (42.13)	20 (5.087)
Exclusive breastfeeding	No	99 (25.132)	14 (3.55)
	Yes	250 (63.45)	31 (7.876)
Weight for age	Normal	210 (53.29)	23 (5.843)
	Underweight	139 (35.27)	22 (5.58)
Height for age	Normal	219 (55.58)	32 (8.142)
	Stunted	130 (32.99)	13 (3.29)
Comorbidities	No	262 (66.5)	34 (8.6)
	Yes	87 (22.1)	11 (2.8)

~~Vaccination status~~	~~Fully vaccinated~~	~~152 (38.6)~~	~~14 (3.5)~~
	~~Partially vaccinated~~	~~151 (38.3)~~	~~17 (4.3)~~
	~~Unvaccinated~~	~~46 (11.7)~~	~~14 (3.5)~~
HIV status	Reactive	5 (1.3)	1 (0.2)
	Non-reactive	187 (47.5)	14 (3.5)
	Unknown	157 (39.9)	30 (7.6)

Treatment Outcome and Incidence Rate of Recovery

Regarding the treatment outcome of severe pneumonia, the majority (349: ~~(~~88.58%) of ~~the~~ children recovered, (22: ~~(~~5.58%) died, (14: ~~(~~3.55%) were left against medical advice and (9: ~~(~~2.28%) were discharged with unknown condition (Figure 2). The shortest and longest length of hospital stay was 1 and 29 days respectively. The total person-day (person time risk) contributed by the study participants was 2,211 days. The overall incidence rate recovery from severe pneumonia was 15.78 per 100-person day (95% CI, 14.2 – 17.5) observation.

The median time to recovery from severe pneumonia

The Kaplan-Meier survival curve was used to estimate the survival status of the children with severe pneumonia. According to the Kaplan-Meier survival estimation, the median survival time without using any stratification factors was 4 days (IQR: ~~(~~3, 7). The graph shows a tendency to fall off quickly in the first five days, showing that most children recovered from severe pneumonia during this time period (Figure 3).

Kaplan Meier s~~S~~urvival plot was performed to compare survival probability between categories of different predictors. Accordingly, survival probability among different categorical predictors such

as co-morbidities, the duration of time before seeking health care, weight for age, antibiotics first administered, and antibiotic change were different (See Figure 4).

Comparison of Median Survival Time

To compare the significance difference in median survival times of various categorical predictors, the log-rank test was used. Accordingly, there was a significant difference in median survival time between the categorical predictors such aslike weight for age, height for age, co-morbidity, antibiotics first administered, and antibiotics change. For instance, the median recovery time of children without comorbidities was 4 days (IQR, 3 – 6) but for children with co-morbidities, it was 6 days (IQR, 4 – 11). Children who visited the Hospital within three days of onset of illnessafter their illness had a median recovery time of 4 days (IQR, 2 – 5) while those who were visited after seven days was 6 days (IQR, 4 – 13). For children for whom antibiotics were changed, the median recovery time was 5 days (IQR, 3 – 9) whereas for children for whom no antibiotics were changed, the median recovery time was 4 days (IQR, 3 – 6) but, there was no significant variation of median recovery time among categorical predictors like HIV status, residence, grunting, and age.

Proportional Hazard Assumption Test

The Cox proportional hazard assumption was checked by graphically (log log plot) and statistically (Global test; p-value = 0.37 indicating the Cox proportional hazard assumption was not violated). Finally, Cox-Snell residual test was performed to check the final model adequacy and confirmed that the model is good to fit the data (Figure 5).

Predictors of time to recovery from severe pneumonia

After running univariate Cox regression, predictors such as s~~S~~ex, age, residence, grunting, antibiotic first administered, antibiotic change, vaccination status, height for age, weight for age, weight for height, comorbidities, and duration of time before seeking healthcare were selected as a candidate variable for multivariable Cox regression analysis. After performing~~carrying out the~~ multivariable Cox regression, adjusted hazard ratios with a 95% confidence interval (CI) and a p-value of less than or equal to 0.05 were used to declare statistical significance. Thus, Variables such as~~like~~ ~~the~~ presence of co-morbidities, duration of time before seeking health care, antibiotic first administered and antibiotics change were statistically significant predictors of time to recovery from severe pneumonia in this study.

The recovery time of children with comorbidity is delayed by 30% as compared to children without comorbidity (AHR; 0.70, (95% CI, (0.54 - 0.92)). Regarding the treatment regimen, the recovery time of children treated with ceftazidime and vancomycin as first-line antibiotics during admission was delayed by 71% as compared to children treated with crystalline penicillin (AHR; 0.29, (95% CI, 0.145 - 0.614)). Children for whom antibiotics were changed were 26% less likely to recover quickly than children for whom antibiotics were not changed (AHR; 0.74, (95% CI, 0.57 - 0 .94)). Children with severe pneumonia who came between 4 and 7 days to the Hospital had 27% slower recovery time as compared to children who came within 3 days (AHR; 0.73(95% CI, (0.561-0.959)) and those who came after 7 days of their illness to the Hospital had 42% slower recovery time as compared to the reference group (AHR; 0.58, (95% CI, 0.43 - 0.79)).

Table 3: Multivariable Cox regression analysis for independent predictors of recovery time among children aged 2-59 months with severe pneumonia admitted to the pediatric ward of Jimma University Medical Center (JUMC) from Jan 1, 2018- Dec 31, 2022

Variable	Category	p-value	CHR, 95%CI	AHR, 95%CI
Sex	Male		1	1
	Female	0.038	0.79(0.645-0.988)	0.92(0.727 - 1.155)
Residence	Urban		1	1
	Rural	0.225	0.87(0.711-1.083)	0.94 (0.752 - 1.175)
Age	2-11 months		1	1
	12-35 months	0.226	0.86(0.684-1.093)	1.00(0.736 - 1.382)
	36-59 months	0.204	0.80(0.576-1.124)	1.05(0.678 - 1.636)
Grunting	No		1	1
	Yes	0.122	0.84(0.686-1.045)	0.85 (0 .680 - 1.071)
Antibiotics first administered	Cr. penicillin		1	1
	Ampicillin & Gentamycin	0.000	0.50(0.379-0.665)	**0.59(0.439- 0.798) ****
	Ceftriaxone	0.000	0.55(0.432-0.714)	**0.56(0.426 - 0.727) ****
	Ceftazidime & Vancomycin	0.000	0.23(0.117-0.457)	**0.29(0.143- 0.600) ****
Antibiotic change	No		1	1
	Yes	0.001	0.66(0.532-0.839)	**0.74(0.581 - 0.953) ***
Vaccination status	Fully vaccinated		1	1
	Partially vaccinated	0.734	1.04(0.829-1.303)	1.01 (0.728 - 1.402)
	unvaccinated	0.001	0.57(0.411-0.799)	0.70(0.489 - 1.029)

Time prior to seeking health care	Up to 3 days		1	1
	4 - 7days	0.003	0.67(0.525-0.872)	**0.73 (0.561- 0.959) ***
	Above 7 days	0.000	0.51(0.392-0.669)	**0.58 (0.432 - 0.789) ****
Comorbidities	No		1	1
	Yes	0.000	0.60(0.473-0.774)	**0.70 (0.543 - 0.914) ***
Height for age	Normal		1	1
	Stunted	0.059	0.81(0.651-1.007)	0.85(0.671- 1.072)
Weight for height	Normal		1	1
	Wasted	0.055	1.23(.995-1.528)	1.15(0.919 - 1.439)
Weight for age	Normal		1	1
	Underweight	0.003	0.71(0.576-0.890)	0.89(0.699 - 1.134)

P-Value ≤=< 0.05(*) and ** for p-value < 0.01

Formatted: Justified

DISCUSSION

Children between the ages of 2-59 months with severe pneumonia who were admitted to the pediatric ward of Jimma University Medical Center (JUMC) were assessed for recovery time and its predictors. In the current study, the median recovery time from severe pneumonia was 4 days IQR (3, 7). Different predictors were found to contribute to the recovery time of children admitted with severe pneumonia in this study. Predictors like antibiotic first administered, antibiotic change, duration of time before seeking health care, and comorbidity had significant associations with recovery time.

According to the current study, among children who were admitted to the pediatric ward of Jimma University Medical Center between the ages of 2-59 months, the median recovery time from severe pneumonia was 4 days. This finding is in line with other studies conducted in Debre Markos [29], Hosanna [8] and Gambia. .[12] This finding is longer than the British Thoracic Society guideline [30] and the study conducted in Nepal that estimates the recovery time of 2 days.[31] The variation in the results~~gap~~ may be explained by the difference in socioeconomic status of the research areas, unique circumstances, such as health care setting~~treatment protocols~~, and the age of the study participants, as the study involved Nepalese children between the ages of 2-35 months.

The current finding is lower than a study conducted in Tanzania which estimated the median recovery time of 10 days. [32] The discrepancy could be a result of the time difference between study periods. The difference in the age of the study population could also explain the discrepancy, as the Tanzanian study was conducted among children aged between the ages of 0 and 144 months. The analysis approach (model) used, in~~as~~ the Tanzanian study was logistic regression, which does not take the censoring of study participants into account.~~, and~~ T~~t~~he study design in Tanzanian study

wasn~~n~~ ~~as they conducted~~ a prospective cohort study which is better in generating strong evidence could explain the variability.

According to the study conducted in Poland, patients stay for~~an~~ an average of 8.2 to 10.1 days. [33] This variation might be due to high ~~the fact that in our country Ethiopia, the~~ bed occupancy rate in Ethiopia ~~is high~~ and patients are discharged, as soon as their signs and symptoms subside without sending and waiting for any laboratory confirmation. On the contrary, Poland is ~~but, this country~~ ~~is~~ high-income country compared to Ethiopia ~~our country~~, and their health care system might be well organized and developed.[34] The ~~Due to that the~~ bed occupancy rate in Poland is~~is~~ probably low and people can pay any charge at the hospital that~~; this~~ may create an opportunity for patients to stay longer at the hospital until complete stability from all signs and symptoms of the disease.

Formatted: Superscript

Duration of time prior to~~before~~ seeking health care was an independent significant predictor of the recovery time of severe pneumonia. Children who came to the hospital within three days of illness recovered quicker than those children who came after seven days of illness. This finding is supported by a prospective follow-up study conducted in the Gambia [12] and other retrospective studies conducted in Debre Markos Hospital [29], Ayder Comprehensive Specialized Hospital [22], and Central and North Gondar zones. [21] Lately presented cases of pneumonia~~This could be because the late presenter~~ might develop a complication that needs further investigation to treat and this~~also as the~~ condition in turn increases ~~of the disease increases,~~ the length of time required to recover ~~from it increases~~.[35]

The existence of co-morbidity was another significant predictor that was connected with the length of time needed to recover from severe pneumonia in this study. Those children with any type of co-morbidity were found to stay longer in the hospital than their counterparts who were free of

comorbidities. This finding is consistent with other studies conducted at Debre Markos [29], Central and North Gondar zones [21], and Nepal. [31] The reason why children with co-morbidity stay for a prolonged time at the hospital might be due to the fact that Co-morbidities significantly increase the severity of pneumonia, including the need for oxygen, and are significantly associated with more complications, that increases~~, thus reducing~~ the recovery time.[32] Since the major comorbidity in the current study was severe acute malnutrition, as adequate protein and energy are needed for proper immune system functioning; undernourished children have a compromised immune system and are more vulnerable to developing serious infections and complications~~ could be possible explanation for a prolonged stay among acutely malnourished children~~.[36]

The antibiotic first administered was one of the significant clinical and treatment-related predictors in this study. Children with severe pneumonia who were treated with ceftriaxone and ampicillin and gentamicin had a prolonged recovery time. This might be due to ampicillin and gentamicin being administered together for patients~~ with bacterial meningitis after testing for cerebrospinal fluid (CSF) [36] and for those children who~~ presented with severe acute malnutrition in addition to severe pneumonia.[37] Children with severe pneumonia who were treated with ceftazidime and vancomycin as the first antibiotic at the time of admission stayed longer in the hospital. This might be because~~ these drugs are used to treat seriously ill children and infections caused by *staphylococcus aureus* and gram-negative enteric bacilli pneumonia, especially those with penicillin resistance properties. Furthermore,~~ ceftazidime and vancomycin together administered for children with comorbidity, particularly for severe bacterial infections like endocarditis and invasive methicillin- resistance staphylococcus aureus infections.[38, 39]

Antibiotic change is also a significant predictor of recovery time. Compared to children whose medications were not altered, those whose antibiotics were changed required longer~~engthier~~

Formatted: Font: Italic

hospital stays to recover from severe pneumonia. Antibiotics are altered whenever there is treatment failure on first-line drugs due to drug resistance or selection of inappropriate drug to treat the patients.[40] For this reason, the additional time required forby second-line drugs to act could explain the longer recovery time. This finding is supported by other study conducted in Hosanna.[8]

Strength and Limitations of the Study

Although this study uses five years of data, important variables like parent's income(Wealth index) and educational status were not included. The study also didn't take any laboratory investigation into account such aslike baseline complete blood count, blood culture, and electrolyte sedimentation rate.

CONCLUSION

This study assessed the recovery time from severe pneumonia and its predictors among children aged 2-59 months admitted to the pediatric ward of Jimma University Medical Center (JUMC). The finding of this study revealed that the median recovery time was longer than other similar studies. Late presentation to the hospital, antibiotic first administered, antibiotic change and the presence of co-morbidities were significant predictors that prolonged the recovery time. Healthcare providers should have to give due attention to the identified predictors of recovery time. Measures that help children to recover quickly from their illness should be strengthened.

ABBREVIATIONS

AHR: Adjusted Hazard Ratio; **BSc:** Bachelor of Science; **CEO:** Chief Executive Officer; **CI:** Confidence Interval; **CHR**: Crude Hazard Ratio; **CSF:** Cerebrospinal Fluid; **FMOHE:** Federal Ministry of Health of Ethiopia; **HIV:** Human Immune Virus; **Hib:** Haemophilus influenzae type

b; **ICCM:** Integrated Community Case Management**; IMNCI:** Integrated Management of Newborn and Childhood Illness**; IQR:** Interquartile range**; JUMC:** Jimma University Medical Center**; MRN:** Medical Registration Number**; ORS:** Oral Rehydration Solution**; PCV:** Pneumococcal Conjugate Vaccine**; SAM:** Severe Acute Malnutrition**; SD:** Standard Deviation**; SSA:** Sub-Saharan Africa**; TB:** Tuberculosis**; WHO:** World Health Organization

Ethical Considerations

Prior to~~Before~~ data collection, this research protocol has been submitted to the Institutional Review Board (IRB) of Jimma University and meets the ethical and scientific standards outlined in national and international guidelines. Jimma University, Institute of Health (Reference No JUIH/IRB/361/23) approved the consent wavier. Then a letter of permission was written to Jimma University Medical Center managers. Letter to access the patient's chart was obtained from the chief executive officer (CEO) of Jimma University Medical Center and the health management information system focal person verbally consented to use the patients' medical chart for data collection. The study was conducted following the relevant international guidelines, regulations, and principles of Helsinki declaration. Verbal or written consent was not obtained from the study participants due to the fact that the data were collected from patients' medical charts and there was no direct contact between patients and data collectors. Throughout the study, the information obtained from the patient's medical records was kept entirely confidential and only accessed by the research team, and solely for the purpose of the research. For data collection purposes, medical record number were utilized, and no personal identifiers were gathered or utilized in the research report.

Acknowledgments

The authors would like to acknowledge Jimma University Ethical Review Board for providing the ethical clearance letter and Shenen Gibe General Hospital for their valuable and preliminary support. We also extend our heart full gratitude to all the data collectors and supervisors for their efforts in the data collection and chart review process.

Author contributions

Getu Girma Bekele: Conceptualizations, Data curation, Formal analysis, Investigations, Methodology, Supervision, Validation, Visualization, writing an original draft, and Writing Review and editing the manuscript.

Masrie Getnet: Formal analysis, Investigations, Methodology, Supervision, Visualization, Writing Review and editing the manuscript.

Dawit Regassa: Formal analysis, Investigations, Methodology, Supervision, Visualization, Writing Review and editing the manuscript.

Tola Getachew: Formal analysis, Investigations, Methodology, Supervision, Visualization, Writing Review and editing the manuscript.

Funding

No funds were obtained for this particular study.

Disclosure

No conflict of interest in this particular study.

Formatted: Normal

References

1. McAllister DA, Liu L, Shi T, Chu Y, Reed C, Burrows J, et al. Global, regional, and national estimates of pneumonia morbidity and mortality in children younger than 5 years between 2000 and 2015: a systematic analysis. Lancet Glob Health. 2019;7(1):e47-e57.

2. Nair1 IRH, KLOB, Li Liu2, E, Lukšić4 T, SQ. Epidemiology and etiology of childhood pneumonia in 2010: estimates of incidence, severe morbidity, mortality, underlying risk factors and causative pathogens for 192 countries.

3. UNICEF. A fair chance for every child 2016 [Available from: https://www.unicef.org/serbia/media/1216/file/State%20of%20the%20world%27s%20children%202016.pdf.

4. Pneumonia in children [Available from: https://www.who.int/news-room/fact-sheets/detail/pneumonia.

Formatted: Justified

Formatted: Justified, Line spacing: Double

Formatted: Font: (Default) Times New Roman, 12 pt

Formatted: Font: (Default) Times New Roman, 12 pt

Formatted: Font: (Default) Times New Roman, 12 pt

Formatted: Font: (Default) Times New Roman, 12 pt

Formatted: Font: (Default) Times New Roman, 12 pt

5. Naheed A, Breiman RF, Islam MS, Saha SK, Tabassum Naved R. Disparities by sex in care-seeking behaviors and treatment outcomes for pneumonia among children admitted to hospitals in Bangladesh. PLoS One. 2019;14(3):e0213238.

6. Helle Ostergaard Madsen MH, Ashima Rani Das, Prabhakar D Moses, Winsley Rose, Mammen Puliyel, Flemming Konradsen, K R John, Anuradha Bose. Costing of severe pneumonia in hospitalized infants and children aged 2-36 months, at a secondary and tertiary level hospital of a not-for-profit organization. 2019.

7. Hauck K ZX. How Dangerous is a Day in Hospital?: A Model of Adverse Events and Length of Stay for Medical Inpatients. Medical Care. 2011.

8. Tirore LL, Abame DE, Sedoro T, Ermias D, Arega A, Tadesse T, et al. Time to Recovery from Severe Pneumonia and Its Predictors Among Children 2-59 Months of Age Admitted to Pediatric Ward of Nigist Eleni Mohammed Memorial Comprehensive Specialized Hospital, Hossana, Ethiopia: Retrospective Cohort Study. Pediatric Health Med Ther. 2021;12:347-57.

9. Mulholland E, Smith L, Carneiro I, Becher H, Lehmann D. Equity and child-survival strategies. Bull World Health Organ. 2008;86(5):399-407.

10. Zhang S, Sammon PM, King I, Andrade AL, Toscano CM, Araujo SN, et al. Cost of management of severe pneumonia in young children: systematic analysis. Journal of Global Health. 2016;6(1).

11. Kamolz L-P, Marfil-Garza BA, Belaunzarán-Zamudio PF, Gulias-Herrero A, Zuñiga AC, Caro-Vega Y, et al. Risk factors associated with prolonged hospital length-of-stay: 18-year retrospective study of hospitalizations in a tertiary healthcare center in Mexico. Plos One. 2018;13(11).

12. Kuti BP, Adegoke SA, Oyelami OA, Ota MO. Predictors of prolonged hospitalisation in childhood pneumonia in a rural health centre. South African Journal of Child Health. 2014;8(1).

13. Black RE, Morris SS, Bryce J. Where and why are 10 million children dying every year? Lancet. 2003;361(9376):2226-34.

14. WHO. Revised WHO classification and treatment of childhood pneumonia at health facilities.

15. Ministry of Health, Federal Democratic Republic of Ethiopia (2015). Ethiopia: Health Sector Transformation Plan 2015/16 – 2019/20

(2008-2012 EFY). [Available from: https://ephi.gov.et/wp-content/uploads/2023/01/2.-Ethiopia-Health-Sector-Transformation-Plan-I-2015-2020-Endline-Review.pdf.

16. Alamneh YM, Adane F. Magnitude and Predictors of Pneumonia among Under-Five Children in Ethiopia: A Systematic Review and Meta-Analysis. J Environ Public Health. 2020;2020:1606783.

17. Tegenu K, Geleto G, Tilahun D, Bayana E, Bereke B. Severe pneumonia: Treatment outcome and its determinant factors among under-five patients, Jimma, Ethiopia. SAGE Open Med. 2022;10:20503121221078445.

18. Jimma University Medical Center (JUMC) [Available from: https://www.lucaincroccifoundation.org/JUMC.

19. Jimma university medical center health managment information system. 2023.

20. Schoenfeld DA. Sample size formula for the proportional hazards regression model.

21. Tamirat ZG, Gelagay AA, Boke MM. Time to recovery and its predictors among children age of 2 up to 59 months admitted with severe community acquired pneumonia in Public Hospitals of Central and North Gondar Zones, Ethiopia 2021. 2022.

Formatted: Font: (Default) Times New Roman, 12 pt

Formatted: Font: (Default) Times New Roman, 12 pt

Formatted: Font: (Default) Times New Roman, 12 pt

Formatted: Font: (Default) Times New Roman, 12 pt

22. Amare RA, Fisseha G, Berhe A, Tirore LL. Incidence of recovery from severe pneumonia and its predictors among children 2-59 months admitted to pediatric ward of Ayder Comprehensive Specialized Hospital, Tigray, Ethiopia: A retrospective Cohort study. J Family Med Prim Care. 2022;11(9):5285-92.

23. what-is-pneumonia [Available from: https://www.thoracic.org/patients/patient-resources/resources/what-is-pneumonia.pdf.

24. Ndu IK, Ekwochi U, Osuorah CD, Onah KS, Obuoha E, Odetunde OI, et al. Danger Signs of Childhood Pneumonia: Caregiver Awareness and Care Seeking Behavior in a Developing Country. Int J Pediatr. 2015;2015:167261.

25. Ayalew T. Exclusive breastfeeding practice and associated factors among first-time mothers in Bahir Dar city, North West Ethiopia, removed: A community based cross sectional study. Heliyon. 2020;6(9):e04732.

26. Tiruneh CM, Emiru TD, Tibebu NS, Abate MW, Nigat AB, Bantie B, et al. Underweight and its associated factors among pediatrics attending HIV Care in South Gondar Zone public health facilities, Northwest Ethiopia, 2021. BMC Pediatrics. 2022;22(1).

27. Luzingu JK, Stroupe N, Alaofe H, Jacobs E, Ernst K. Risk factors associated with under-five stunting, wasting, and underweight in four provinces of the Democratic Republic of Congo: analysis of the ASSP project baseline data. BMC Public Health. 2022;22(1).

28. Lee SW. Kaplan-Meier and Cox proportional hazards regression in survival analysis: statistical standard and guideline of Life Cycle Committee. Life Cycle. 2023;3.

29. Patman S, Mengist B, Tesfa M, Kassie B. Time to recovery and predictors of severe community-acquired pneumonia among pediatric patients in Debre Markos referral hospital, North West Ethiopia: A retrospective follow-up study. Plos One. 2020;15(9).

Formatted: Font: (Default) Times New Roman, 12 pt

Formatted: Font: (Default) Times New Roman, 12 pt

30. Michael Harris JC, Nicky Coote, Penny Fletcher et al. British Thoracic Society Guidelines for the Management of Community Acquired Pneumonia in Children: Update 2011.

31. Thorne C, Basnet S, Sharma A, Mathisen M, Shrestha PS, Ghimire RK, et al. Predictors of Duration and Treatment Failure of Severe Pneumonia in Hospitalized Young Nepalese Children. Plos One. 2015;10(3).

32. Caggiano S, Ullmann N, De Vitis E, Trivelli M, Mariani C, Podagrosi M, et al. Factors That Negatively Affect the Prognosis of Pediatric Community-Acquired Pneumonia in District Hospital in Tanzania. Int J Mol Sci. 2017;18(3).

33. Gajewska M, Lewtak K, Scheres J, Albrecht P, Gorynski P. Trends in Hospitalization of Children with Bacterial Pneumonia in Poland. Cent Eur J Public Health. 2016;24(3):188-92.

34. Smarzewska D, Wereda WS, Jonczyk JA. Assessment of the Health Care System in Poland and Other OECD Countries Using the Hellwig Method. Int J Environ Res Public Health. 2022;19(24).

35. MacKian S. A review of health seeking behaviour: problems and prospects.

36. Kidane GF, Zereabruk K, Aberhe W, Hailay A, Mebrahtom G, Gebremeskel GG, et al. Time to recovery from severe acute malnutrition and its predictors among under five children admitted to therapeutic feeding units of general and referral hospitals in Tigray, Ethiopia, 2020: a prospective cohort study. BMC Pediatr. 2023;23(1):325.

37. UNICEF. Mangment of severe acute malnutrition in children: working towards resultsat sale: 2015.

38. Matsumoto K, Takesue Y, Ohmagari N, Mochizuki T, Mikamo H, Seki M, et al. Practice guidelines for therapeutic drug monitoring of vancomycin: a consensus review of the Japanese

Society of Chemotherapy and the Japanese Society of Therapeutic Drug Monitoring. J Infect Chemother. 2013;19(3):365-80.

39. Frymoyer A, Hersh AL, Benet LZ, Guglielmo BJ. Current recommended dosing of vancomycin for children with invasive methicillin-resistant Staphylococcus aureus infections is inadequate. Pediatr Infect Dis J. 2009;28(5):398-402.

40. Berkley JA, Muro RP, Masoza TS, Kasanga G, Kayange N, Kidenya BR. Predictors and outcome of first line treatment failure among under-five children with community acquired severe pneumonia at Bugando Medical Centre, Mwanza, Tanzania: A prospective cohort study. Plos One. 2020;15(12).

Manuscript Title: Time-to-Recovery from Severe Pneumonia and its Predictors among Children 2-59 Months of Age Admitted to Pediatric Ward of Jimma University Medical Center, Southwest Ethiopia, 2023: A Retrospective Cohort Study.

Manuscript ID: PONE-D-2404566

Authors: Getu Girma, Masrie Getnet, Dawit Regassa, Tola Getachew

Journal: PLOS ONE

Article type: Original Research

Submitted on: November 29, 2024, 11:59pm

Dear Respected Editors/ Reviewers,

First of all, we would like to appreciate the reviewers for your complimentary comments and suggestions to our manuscript entitled **"Time to Recovery from Severe Pneumonia and its Predictors among Children 2-59 Months of Age Admitted to Pediatric Ward of Jimma University Medical Center, Southwest, Ethiopia, 2023: A Retrospective Cohort Study"**. We highly appreciate the comprehensive feedback throughout the reviewing process and are grateful for the endorsement of this manuscript for publication in your journal **"PLOS ONE".** In this revised version of the manuscript, we did our best to address all the comments and suggestions raised by the editors and the reviewers. We have submitted a revised copy of the manuscript both in track-changed and without track-changed (clean file) including a response to the reviewer's file, and supplementary files.

PLOS ONE| Editorial Office - Collaborative Peer Review Team Comments to Author:

Independent Review Report, Reviewer 1

Abstract: inadequately clear

Response: We appreciate the comments and suggestions; the recommendation you raised were briefly written but we deleted them for the sake of word limit and now we have made corrections in the specified section as per the comments.

Background: needs further enrichment for relevance and contextual factors

Response: we have made corrections in the specified section as per the comments.

Methods: should be presented in explicit and comprehensive manner including ethical considerations.

Response: Thank you for your constructive comments and we made appropriate corrections in the specified section as per the comments

Operational definition: Good if all are referenced with WHO standards or any other = optional

Response: Thank you. We made corrections in the section

Survival time: What is its difference from that of time-to-recovery?

Response: Thank you for the comments, survival time is the general term that indicates the time it takes for the certain event(out come of interest) to occur and our event in this particular study was recovery. Indeed there is no such difference between them and we have deleted survival time

Do you have graph?

Response: Thank you for the comments, yes we do have graphs like Kaplan Meier survival curve and Cox-snell.

Why? What is unique about residence?

Response: Thank you for the comments, in this particular study event(recovery) was higher than censoring but we made a mistake about residence during typing (simply typing error) and so we made corrections in the section.

Results and discussion: lacks brief and comprehensive presentation with appropriate references and justification

Response: Thank you. We made corrections in the section.

Table 2 Revisit your percentages

Response: Thank you. We made corrections in the section.

Vaccination status: Repeated

Response: Thank you. We mistakenly repeated and we deleted repetition.

After performing multivariable Cox regression, adjusted hazard ratios with a 95% confidence interval (CI) and a p-value of less than or equal to 0.05. Thus, variables like the presence of comorbidities, duration of time before seeking health care, antibiotic first administered and antibiotics change were statistically significant predictors of time to recovery from severe pneumonia in this study. Seems complete?

Response: Thank you. We made corrections in the section as After performing multivariable Cox regression, adjusted hazard ratios with a 95% confidence interval (CI) and a p-value of less than or equal to 0.05 were used to declare statistical significance. Thus, Variables such as presence of co-morbidities, duration of time before seeking health care, antibiotic first administered and antibiotics change were statistically significant predictors of time to recovery from severe pneumonia in this study.

Table 3: Age did not contribute. Why?

Response: Thank you. After controlling the potential confounding factors, there is no siginficant difference in the age between 2-11 months and 12-35 months, indicating that the crude effect was likely not due to the factors being studied, but rather the other confounding factors.

In discussion part:

Have you read any single article that tells you this information?

Response: Thank you for the comments and we cited the references as per the comments in our manuscript.

Rewrite. It is a long sentence to capture your idea. So, split into two and rewrite it again

Rewrite in a clear way

Response: Thank you. We made corrections in the section.
