# Supplementary material for: Time-to-recovery from severe pneumonia and its predictors among children 2–59 months of age admitted to the pediatric ward of Jimma University Medical Center, Southwest Ethiopia, 2023: A retrospective cohort study
Source: PLoS One. 2025 Apr 15;20(4):e0316839. doi: 10.1371/journal.pone.0316839 (PMC11999157; doi:10.1371/journal.pone.0316839)
Supplement: Attachment — Submitted filename: Manuscript PLOS one.docx [file pone.0316839.s001.docx]

Time-to-recovery from Severe Pneumonia and its Predictors	among Children 2-59	Months	of Age Admitted to Pediatric Ward of Jimma University Medical Center, Southwest Ethiopia, 2023: A Retrospective CohortStudy

Getu Girma Bekele^1*^, Masrie Getnet^2^, Dawit Regassa^2^, Tola Getachew Bekele^1^

**Authors’ Affiliation**

^1^ Jimma University Medical Center, Jimma, Ethiopia

^2^Jimma University, Institute of Health, Faculty of Public Health, Department of Epidemiology, Jimma, Ethiopia

**Authors’ Email Addresses**

Getu Girma Bekele: Email:[getgirma2013@gmail.com](mailto:getgirma2013@gmail.com), Jimma, Ethiopia

Masrie Getnet: [masriegetnet16biostat@gmail.com](mailto:masriegetnet16biostat@gmail.com), Jimma, Ethiopia

Dawit Regassa**:** [dawitregassa4@gmail.com](mailto:dawitregassa4@gmail.com), Jimma, Ethiopia

Tola Getachew Bekele: [tolagetachew613@gmail.com](mailto:tolagetachew613@gmail.com), Jimma, Ethiopia

**Corresponding Author:** Getu Girma Bekele **Email:** [getgirma2013@gmail.com](mailto:getgirma2013@gmail.com)

Phone: +251937640136

**Abstract**

**Background:** Pneumonia is an inflammation of the lung parenchyma. The World Health Organization estimates 156 million cases of pneumonia, including up to 20 million cases severe enough to require hospitalization, and 1.2 million deaths each year among under-five children. Ethiopia is ranked sixth among top fifteen countries in terms of pneumonia related morbidity and mortality. The study aimed to assess the time to recovery from severe pneumonia and its predictors among children aged 2-59 months admitted to the pediatric ward of Jimma University Medical Center; Southwest Ethiopia, 2023.

**Methods:** A facility-based retrospective cohort study was carried out among 426 children aged between 2 and 59 months. Five years of medical records, from 2018 – 2022, were reviewed. A simple random sampling technique was used. Data entry was done using epidata version 4.6 and exported to and analyzed by STATA version 15. Kaplan Meier’s plot was used to compare the survival probability of different groups and the multivariable Cox regression model was used to investigate the independent effect of covariates on recovery time.

**Result:** The median recovery time was 4 days (IQR: 3, 7). Incidence rate of recovery was 15.78 per 100-person day (95% CI 14.2 – 17.5). The presence of comorbidity (AHR; 0.7, 95% CI (0.54 – 0.91)), being treated with ceftazidime and vancomycin (AHR; 0.29, 95% CI (0.14 – 0.60)), antibiotic change (AHR; 0.74, 95% CI (0.58 – 0.95)) and late presentation to the Hospital (AHR; 0.58, 95% CI (0.43 -0.78)) were statistically significant predictors that prolong recovery time.

**Conclusion:** The median recovery time was longer than other similar studies. Therefore, due attention should be given to the identified predictors of the recovery time.

**Keywords:** Time to recovery, Severe Pneumonia, predictors, Children 2-59 Months, Jimma

# **INTRODUCTION**

The World Health Organization estimates that 156 million cases of pneumonia occur annually in children under the age of five, including up to 20 million cases severe enough to require hospitalization, and 1.2 million deaths each year.^(1)^ The greatest incidence was occurring in south Asia (2,500cases per 100,000 children) and west and central Africa (1,620 cases per 100,000 children).^(2)^ Approximately 490,000 children under the age of five died of pneumonia in Sub Saharan Africa (SSA) in 2016.^(3)^ In Ethiopia, approximately 3,370,000 children contract pneumonia each year which accounts for 14% of all deaths of children under five years old.^(4)^

Severe pneumonia is the major cause of hospitalization in under-five children. Among 156,847 admitted children identified in seven hospitals in Bangladesh, pneumonia was the most diagnosed (17.9%).^(5)^ Hospitalization alone was found to contribute to 46.8% of a household price for one severe pneumonia episode.^(6)^ Effective management of pneumonia is not just only the ratio of the children recovered, but also the time of recovery. Spending each night in the hospital increases the risk of adverse medication reactions, ulcers, and infections by 0.5%, 0.5%, and 1.6%, respectively.^(7)^ Tremendous independent clinical factors marked up mortality and duration of stay associated with pneumonia, with significant variability among hospitals. Some studies identify different forms of malnutrition, comorbidities, diarrhea, antibiotics first administered, being underweight, and age are the major factors that affect recovery time.^(8)^

A longer hospital stay has a significant negative impact on the admitted child, his or her parents, and the healthcare system, particularly in societies with resource-limited settings and no health insurance scheme.^(9)^ Between 26.6% and 115.8% of monthly household income is spent on the direct medical costs of treating severe pneumonia in a hospital setting. Non-medical costs (such as travel and food) during a child's hospital stay for severe pneumonia account for 9.0% and 31.0% of the household's monthly income in Kenya and Guinea, respectively.^(10)^ Patients with prolonged length of stay extremely account for the consumption of more hospital resources, Hospital acquired infection, and denies critically ill patients’ timely access to treatment contribute to capacity shortages.^(11)^ Hence, these children are often discharged against medical advice without receiving full therapy, which results in an increased rate of readmission and complications such as pneumonic effusion, empyema, necrotizing pneumonia, and lung abscess.^(12)^

The Federal Ministry of Health of Ethiopia (FMOHE) has been working to prevent and reduce under-five mortality by introducing Pneumococcal Conjugate Vaccine (PCV) and Haemophilus influenzae type b (Hib) vaccine, as well as by putting into practice strategies such as integrated community case management (ICCM) to train, support, and supply community health workers to provide diagnostic, treatment and referral services and integrated management of newborn and child illnesses (IMNCI) to improve case management skills of health care providers, improving health systems to provide quality care and improving family and community health practice for health, growth, and development.^(13, 14)^ Despite those-mentioned programs and endeavors, still the main causes of illness and death in children under the age of five in Ethiopia is pneumonia; with an approximated contribution of around 40,000 fatalities per year.^(15)^

Pneumonia is one of the major public health importances. Despite the intervention that has been implemented in Ethiopia, pneumonia continues as one of the main reasons of hospitalization and mortality among under-five children ^(9)^. The widespread nature of the problem in Ethiopia has already killed thousands of children hospitalized with severe pneumonia, which requires everlasting solutions^(16)^. Understanding the recovery time helps the health managers in evaluating the quality of health care delivered by the given health institution because the average length of stay provides information about the efficiency of care delivery, the standard of care provided, and the effective use of resources. Further, it can provide valuable information to physicians and other healthcare providers for counseling parents or caregivers about the duration of hospitalization so that the caregivers can timely mobilize resources for a child’s hospital care.

To the best of our knowledge and as far as our literature search is concerned, there are limited studies and evidence on the recovery time from severe pneumonia and its predictors in the current study area. As a result, the aim of this study was to estimate the median time of recovery from severe pneumonia and its predictors in children aged 2-59 months admitted to the pediatric ward of Jimma University Medical Center, Southwest Ethiopia, 2023.

# **MATERIALS AND METHODS**

## Study Area and Period

The study was conducted at Jimma University Medical Center (JUMC). It is the only teaching and tertiary federal hospital found in southwest Ethiopia. Geographically, it is located in Jimma town, Oromia region, 353 km southwest of Addis Ababa, the capital city of Ethiopia. It was established in 1930 E.C. by Italian invaders for the service of their soldiers. Currently, JUMC provides services for approximately 18,289 inpatients on 800 beds, 232,000 outpatient attendants, 79,000 emergency service cases, and 6,500 deliveries in a year coming to the hospital from the catchment area. JUMC serves as a referral hospital in southwest Ethiopia. The population in the catchment area is estimated to be 15 million.^(17, 18)^

The pediatric department of JUMC has a pediatric outpatient, pediatric ward, and pediatric intensive care unit. The pediatric ward has three units namely level I, level II, and nutritional rehabilitation unit (NRU) with a total of 78 beds, and provides services for an average of 55-65 patients including severe pneumonia. On average, over 400 children were diagnosed with severe pneumonia each year in this hospital. Children diagnosed with severe pneumonia were directly admitted to level I and treated by senior Pediatricians, Residents, Nurses, and Medical Interns. The pedantic ward has a total of 60 health professionals with 35 nurses, 7 senior physicians, and 16 residents. There are also pharmacy professionals and laboratory technologists who were working there by rotation.^(18)^ The five-year data were reviewed from January 1, 2018, to December 31, 2022. The patient charts were retrieved from May 14- June 15, 2023.

## Study Design

A facility-based retrospective cohort study was conducted.

## Population

### **Source population**

All children 2 to 59 months of age who were admitted to the pediatric ward of Jimma University Medical Center (JUMC) with severe pneumonia were the target population.

### **Study population**

All children with severe pneumonia aged 2-59 months who were managed at the pediatric ward of Jimma University Medical Center (JUMC) from January 1, 2018- December 31, 2022 were the study population.

## Eligibility Criteria

### **Inclusion criteria**

All children with severe pneumonia aged 2-59 months who were admitted to the pediatric ward of Jimma University Medical Center (JUMC) from January 1, 2018- December 31, 2022, were included.

### **Exclusion criteria**

Children’s charts with no Medical Registration Number (MRN), admission date, discharge date, and no or ambiguous age were excluded.

## Sample Size Determination

The sample size was calculated by using the Schoenfeld formula for survival analysis by taking the following assumptions into account (Ref).

- Significance level (α) (two-sided) =0.05, $Z_{\alpha/2}=1.96$
- Power = 80%,$\beta=0.2$ $Z_{\beta=}$0.842
- AHR = Adjusted hazard ratio was used from different similar studies
- E = number of events (recovery)
- Pr(E) = probability of recovery was used from different similar studies
- Finally, a 10% non-response rate (NRR) for an incomplete chart was added
- $E=\left( z\alpha+z\beta\right)2$/ (ln AHR) ^2^ P (1-P), n = E/Pr(E)^(19)^

The final largest sample size **426** was considered to conduct the study.

## Sampling Technique and Procedure

### **Sampling technique**

A simple random sampling technique was used to select the study population.

### **Sampling procedure**

The registry logbook of all children who were admitted with severe pneumonia to the pediatric ward of Jimma University Medical Center (JUMC) from January 1, 2018 – December 31, 2022, was reviewed. Since there was no case-specific registration logbook, the list of all children aged 2–59 months admitted with severe pneumonia from January 1, 2018 – December 31, 2022, was prepared by excluding those with no MRN, admission date, incomplete and ambiguous age. A total of 1985 children with severe pneumonia were noted from the registration logbook during the study period, of which 1697 completed charts were available. Then, 426 charts were sampled using a simple random sampling technique by a computer-generated method (Figure 1).

## Data Collection Tool and Procedure

### **Data collection tool**

The data were collected by using a structured checklist adapted from previous similar studies (^12, 20, 21, 22)^. The tool was designed based on the study objectives and contains socio-demographic characteristics, clinical and treatment-related factors, vaccination and nutritional status as well as comorbidities.

### **Data collection procedure**

The unique medical record number of each patient was used to get their records (charts). All available information on the patient chart was checked. Then, all the relevant variables were extracted from patient charts using an English version data extraction tool to meet the study objectives. Two skilled BSc nurses working in the pediatric ward of Jimma University Medical Center (JUMC) extracted the data from patient charts under the supervision of an MSc holder in pediatric and child health working in the pediatric ward of JUMC as a Head Nurse. Training was given to data extractors and supervisor regarding the significance of the study and ways of the data extraction process by the principal investigator.

## Study Variables

### **Outcome variable**

Time to recovery from severe pneumonia measured in days.

### **Independent variables**

#### Socio-demographic characteristics

The socio-demographic characteristics include the age, sex of the children, residence, and health insurance scheme.

#### Clinical and treatment-related factors

Under clinical and treatment-related factors presence of danger signs at admission, the duration time before seeking health care, grunting, head nodding, HIV status, antibiotics first administered, antibiotic change, and convulsion were used.

#### Nutrition and vaccination status

Nutrition and vaccination status include vaccination status, height for age, weight for height, weight for age, exclusive breastfeeding, inability to feed, and different comorbidities.

## Operational Definition

**Severe pneumonia:** children presented with inability to drink, persistent vomiting, oxygen saturation less than 90%, convulsion, and lethargy or reduced level of consciousness.^(23)^

**Time to recovery:** The time from admission to when the child is discharged from the hospital due to improvement in symptoms calculated by number of days.

**Median recovery time:** the time in which half (50%) of the children had recovered.

**Censored:** A child with severe pneumonia who has been admitted to the ward and happens to be dead, self‑discharged, or with unknown outcome status.

**Survival time:** is the time starting from the date of admission to recovery from severe pneumonia.

**Comorbidity**: Any disease, acute or chronic, present during admission in addition to severe pneumonia.

**Danger signs**: Children with difficult breathing, chest in drawing, oxygen saturation less than 90%, unconsciousness ^(24)^.

**Vaccination status:** Fully vaccinated which refers to children who had received all forms of vaccination; Partially vaccinated (Children who had taken at least one PCV dose) and unvaccinated (Children who have never received PCV or any form of vaccination).

**Discharge status:** indicate the condition (reason) for the patient’s termination from the program.

**Exclusive breastfeeding**: Children who were introduced to only breast milk except for drugs, ORS (Oral Rehydration Solution), during the first six months of age.^(25)^

**Underweight:** Children having weight for age is less than -2SD (standard deviation).^(26)^

**Wasting:** Children having weight for height is less than -2SD (standard deviation).^(27)^

**Stunting:** Children having height for age is less than -2SD (standard deviation).^(27)^

## Data Management

### Data quality assurance

Data collectors were given orientation on the content of the data extraction tool and procedure. Two skillful BSc nurses working in the pediatric ward of Jimma University Medical Center (JUMC) reviewed the records after they were given training by the principal investigator. Before the actual data collection, the data extraction checklist was tested on 21 medical charts at Shenen Gibe Hospital to ensure that the data extraction format agreed with the objectives of the study. During the data collection process, regular supervision was done by the supervisor to ensure the quality of the data. Throughout the data collection process, the primary investigator and the supervisor routinely examined the accuracy and completeness of the data collected. Data entry and cleaning were done daily and timely feedback was being provided to the data collectors.

### **Data Analysis Procedure**

The data were entered using epidata version 4.6, exported to and analyzed using STATA version 15. The data were coded and cleaned before the analysis. The data were described in terms of central tendency (median) and dispersion (interquartile range) for continuous data and frequency distribution for categorical data and presented using graphs, tables, and text. A day was used to calculate the recovery time. Kaplan Meier’s plot was used to compare the survival experience of different groups of patients by using survival curves. The association between the independent variable and time to recovery from severe pneumonia was analyzed using the Cox proportional hazard regression. Log rank test was used to identify the presence of significant difference in median survival time between different groups (Ref).

Univariate analysis was performed and predictors that have an association with the outcome variable at a p-value of 0.25 or less were entered into the multivariable Cox regression model. The presence of Multicollinearity was checked using the variance inflation factor. A variance inflation factor value of <10 was used as a cutoff point for indicating the absence of multicollinearity. A Cox proportional hazard assumption was checked for each predictor graphically using a log minus log plot of survival and statistically using the global residual test. The final model adequacy was tested by using the Cox-Snell residual test and the baseline distribution of the cumulative hazard function of residuals against Cox-Snell residuals follows 45 degree indicating adequately fitted data. Adjusted hazard ratio (AHR) with a 95% confidence interval (CI) was estimated by multivariable Cox regression and a p-value less than or equal to 0.05 was declared the presence of a statistical significant association between the recovery time and explanatory variable.

# **Results**

## Socio-demographic and Treatment-related characteristics of Study Population

The study included records of children 2-59 months of age admitted to the pediatric ward of Jimma University Medical Center (JUMC) with a diagnosis of severe pneumonia for five consecutive years (2018-2022). From the samples of 426, information was extracted from 394 patient charts making the response rate 92.5%. The female-to-male ratio was 1:1.21 and 52.28% of the study participants came from the countryside. The median age of the study participants was 10 months (IQR: 5 - 21) and the majority (218: 55.33%) of them were under the age group of 2-11 months. Almost two third (244: 62%) of the study participants were not enrolled in health insurance schemes.

About 47.72% of the children visited the hospital within three days of becoming ill. Danger signs were present in about 135 (34.26%) of the children at admission. The commonest danger signs were difficult breathing and severe chest in-drawing accounting for 30 (7.61%) and 25(6.35%), respectively. Among 394 children, 188 (47.72%) and 71 (18.02%) had grunting and convulsion, respectively. Crystalline penicillin was given to 166 (42.35%) of the children at admission as a first-line treatment and antibiotics were changed for 128 (33%) of the children (Table 1).

Table 1: Frequency distribution of socio-demographic and treatment-related characteristics of children aged between 2-59 months with severe pneumonia admitted to the pediatric ward of Jimma University Medical Center (JUMC) from Jan 1, 2018-Dec 31, 2022(n=394)

| Variable | Category | Status | |
| --- | --- | --- | --- |
|  |  | Recovered (%) | Censored (%) |
| Age in month | 2-11  12-35  36-59 | 196 (49.7)  111 (28.2)  42 (10.7) | 22 (5.6)  17 (4.3)  6 (1.5) |
| Sex | Male  Female | 191 (48.5)  158 (40.1) | 25 (6.3)  20 (5.1) |
| Danger signs | No  Yes | 238 (60.4)  111 (28.2) | 21 (5.3)  24 (6.1) |
| Residence | Urban  Rural | 18 (4.6)  27 (6.9) | 170 (43.1)  179 (45.4) |
| Antibiotics change | No  Yes | 229 (59.0)  114 (29.4) | 31 (7.99)  14 (3.61) |
| Antibiotic first given | Crystalline penicillin Ampicillin and gentamicin Ceftriaxone Ceftazidime & vancomycin | 152 (38.78)  77 (19.64)  109 (27.81)  9 (2.3) | 14 (3.57)  12 (3.06)  16 (4.08)  3 (0.77) |
| Grunting | No  Yes | 179 (45.43)  170 (43.15) | 27 (6.85)  18 (4.57) |

## Vaccination, Nutritional Status, and Comorbidities of the Study Population

Most (281: 71.32%) of the children had a history of exclusive breastfeeding. Of the study participants, about 166(42.13%) of the children were fully vaccinated whereas 60(15.23%) were unvaccinated. Stunting, wasting, and being underweight were found in 143 (36.29%), 186 (47.21%), and 161 (40.86%) of the study participants, respectively. About 82 (20.81%) of the children were born before the expected date of delivery. Almost 201(51%) of the children tested non-reactive for HIV and 6 (1.53%) of the children among the study participants were found reactive for the HIV test. About 98 (24.87%) of the children admitted with severe pneumonia had different comorbidities. Severe acute malnutrition was one of the most common diseases in addition to severe pneumonia among the study participants. Other medical conditions like bronchial asthma, down syndrome, and pertussis accounted for 9 (2.29%) (Table 2).

Table 2: Frequency distribution of vaccination, nutritional status, and comorbidities of children aged between 2-59 months with severe pneumonia admitted to the pediatric ward of Jimma University Medical Center (JUMC) from Jan 1, 2018-Dec 31, 2022(n=394)

| Variable | | Category | | Status | |
| --- | --- | --- | --- | --- | --- |
|  |  |  |  | Recovered (%) | Censored (%) |
| Vaccination status | | Fully vaccinated  Partially vaccinated  Unvaccinated | | 152 (38.57)  151 (38.32)  46 (11.67) | 14 (3.55)  17 (4.31)  14 (3.55) |
| Weight for height | | Normal  Wasted | | 183 (46.44)  166 (42.13) | 25 (6.3)  20 (5.07) |
| Exclusive breastfeeding | | No  Yes | | 99 (25.12)  250 (63.45) | 14 (3.55)  31 (7.86) |
| Weight for age | | Normal  Underweight | | 210 (53.2)  139 (35.2) | 23 (5.83)  22 (5.58) |
| Height for age | | Normal  Stunted | | 219 (55.5)  130 (32.9) | 32 (8.12)  13 (3.29) |
| Comorbidities | No  Yes | | 262 (66.5)  87 (22.1) | | 34 (8.6)  11 (2.8) |
| Vaccination status | Fully vaccinated  Partially vaccinated  Unvaccinated | | 152 (38.6)  151 (38.3)  46 (11.7) | | 14 (3.5)  17 (4.3)  14 (3.5) |
| HIV status | Reactive  Non-reactive  Unknown | | 5 (1.3)  187 (47.5)  157 (39.9) | | 1 (0.2)  14 (3.5)  30 (7.6) |

## Treatment Outcome and Incidence Rate of Recovery

Regarding the treatment outcome of severe pneumonia, the majority (349: 88.58%) of children recovered, 22 (5.58%) died, 14 (3.55%) were left against medical advice and 9 (2.28%) were discharged with unknown condition (Figure 2). The shortest and longest length of hospital stay was 1 and 29 days, respectively. The total person-day (person time risk) contributed by the study participants was 2,211 days. The overall incidence rate recovery from severe pneumonia was 15.78 per 100-person day (95% CI, 14.2 – 17.5) observation.

## The Median Time to Recovery from Severe Pneumonia

The Kaplan-Meier survival curve was used to estimate the survival status of children with severe pneumonia. According to the Kaplan-Meier survival estimation, the median survival time without using any stratification factors was 4 days (IQR: 3, 7). The graph shows a tendency to fall off quickly in the first five days, showing that most children recovered from severe pneumonia during this time period (Figure 3).

Kaplan Meier survival plot was performed to compare survival probability between categories of different predictors. Accordingly, survival probability among different categorical predictors such as comorbidities, the duration of time before seeking health care, weight for age, antibiotics first administered, and antibiotic change were different (Figure 4).

## Comparison of Median Survival Time

To compare the significance difference in median survival times of various categorical predictors, the log-rank test was used. Accordingly, there was a significant difference in median survival time between the categorical predictors like weight for age, height for age, comorbidity, antibiotics first administered, and antibiotics change. For instance, the median recovery time of children without comorbidities was 4 days (IQR, 3 – 6) but for children with comorbidities, it was 6 days (IQR, 4 – 11). Children who visited the Hospital within three days after their illness had a median recovery time of 4 days (IQR, 2 – 5) while those who were visited after seven days was 6 days (IQR, 4 – 13). For children for whom antibiotics were changed, the median recovery time was 5 days (IQR, 3 – 9) whereas for children for whom no antibiotics were changed, the median recovery time was 4 days (IQR, 3 – 6) but, there was no significant variation of median recovery time among categorical predictors like HIV status, residence, grunting, and age.

## Proportional Hazard Assumption Test

The Cox proportional hazard assumption was checked graphically (log-log plot) and statistically (Global test; p-value = 0.37 indicating the Cox proportional hazard assumption was not violated). Finally, Cox-Snell residual test was performed to check the final model adequacy and confirmed that the model is good to fit the data (Figure 5).

## Predictors of Time to Recovery from Severe Pneumonia

After running univariate Cox regression, predictors such as sex, age, residence, grunting, antibiotic first administered, antibiotic change, vaccination status, height for age, weight for age, weight for height, comorbidities, and duration of time before seeking healthcare were selected as a candidate variable for multivariable Cox regression analysis. After carrying out the multivariable Cox regression, hazard ratios with a 95% confidence interval (CI) and a p-value less than or equal to 0.05. Thus, variables like the presence of comorbidities, duration of time before seeking health care, antibiotic first administered and antibiotics change were statistically significant predictors of time to recovery from severe pneumonia in this study.

The recovery time of children with comorbidity is delayed by 30% as compared to children without comorbidity (AHR; 0.70, (95% CI, (0.54 - 0.92)). Regarding the treatment regimen, the recovery time of children treated with ceftazidime and vancomycin as first-line antibiotics during admission was delayed by 71% as compared to children treated with crystalline penicillin (AHR; 0.29, (95% CI, 0.145 - 0.614)). Children for whom antibiotics were changed were 26% less likely to recover quickly than children for whom antibiotics were not changed (AHR; 0.74, (95% CI, 0.57 - 0 .94)). Children with severe pneumonia who came between 4 and 7 days to the Hospital had 27% slower recovery time as compared to children who came within 3 days (AHR; 0.73(95% CI, (0.561- 0.959)) and those who came after 7 days of their illness to the Hospital had 42% slower recovery time as compared to the reference group (AHR; 0.58, (95% CI, 0.43 - 0.79)) (Table 3).

Table 3: Multivariable Cox regression analysis for independent predictors of recovery time among children aged 2-59 months with severe pneumonia admitted to the pediatric ward of Jimma University Medical Center (JUMC) from Jan 1, 2018- Dec 31, 2022

| Variable | | Category | p-value | CHR, 95%CI | AHR, 95%CI |
| --- | --- | --- | --- | --- | --- |
| Sex | | Male  Female | 0.038 | 1  0.79(0.645-0.988) | 1  0.92(0.727 - 1.155) |
| Residence | | Urban  Rural | 0.225 | 1  0.87(0.711-1.083) | 1  0.94 (0.752 - 1.175) |
| Age | | 2-11 months  12-35 months  36-59 months | 0.226  0.204 | 1  0.86(0.684-1.093)  0.80(0.576-1.124) | 1  1.00(0.736 - 1.382)  1.05(0.678 - 1.636) |
| Grunting | | No  Yes | 0.122 | 1  0.84(0.686-1.045) | 1  0.85 (0 .680 - 1.071) |
| Antibiotics first administered | Cr. penicillin  Ampicillin & Gentamycin  Ceftriaxone  Ceftazidime & Vancomycin | | 0.000  0.000  0.000 | 1  0.50(0.379-0.665)  0.55(0.432-0.714)  0.23(0.117-0.457) | 1  **0.59(0.439- 0.798) ****  **0.56(0.426 - 0.727) ****  **0.29(0.143- 0.600) **** |
| Antibiotic change | No  Yes | | 0.001 | 1  0.66(0.532-0.839) | 1  **0.74(0.581 - 0.953) *** |
| Vaccination status | Fully vaccinated  Partially vaccinated  unvaccinated | | 0.734  0.001 | 1  1.04(0.829-1.303)  0.57(0.411-0.799) | 1  1.01 (0.728 - 1.402)  0.70(0.489 - 1.029) |
| Time prior to seeking health care | Up to 3 days  4 - 7days  Above 7 days | | 0.003  0.000 | 1  0.67(0.525-0.872)  0.51(0.392-0.669) | 1  **0.73 (0.561- 0.959) ***  **0.58 (0.432 - 0.789) **** |
| Comorbidities | No  Yes | | 0.000 | 1  0.60(0.473-0.774) | 1  **0.70 (0.543 - 0.914) *** |
| Height for age | Normal  Stunted | | 0.059 | 1  0.81(0.651-1.007) | 1  0.85(0.671- 1.072) |
| Weight for height | Normal  Wasted | | 0.055 | 1  1.23(.995-1.528) | 1  1.15(0.919 - 1.439) |
| Weight for age | Normal  Underweight | | 0.003 | 1  0.71(0.576-0.890) | 1  0.89(0.699 - 1.134) |

P-Value ≤ 0.05(*) and ** for p-value < 0.01

# **Discussion**

Children between the ages of 2-59 months with severe pneumonia who were admitted to the pediatric ward of Jimma University Medical Center (JUMC) were assessed for recovery time and its predictors. In the current study, the median recovery time from severe pneumonia was 4 days IQR (3, 7). Different predictors were found to contribute to the recovery time of children admitted with severe pneumonia in this study. Predictors like antibiotic first administered, antibiotic change, duration of time before seeking health care, and comorbidity had significant associations with recovery time.

According to the current study, among children who were admitted to the pediatric ward of Jimma University Medical Center between the ages of 2-59 months, the median recovery time from severe pneumonia was 4 days. This finding is in line with other studies conducted in Debre Markos^(28)^, Hosanna ^(8)^ and Gambia. ^(12)^ This finding is longer than the British Thoracic Society guideline ^(29)^ and the study conducted in Nepal that estimates the recovery time of 2 days.^(30)^ The variation in the results may be explained by the difference in socioeconomic status of the research areas, unique circumstances, such as treatment protocols, and the age of the study participants, as the study involved Nepalese children between the ages of 2-35 months.

The current finding is lower than a study conducted in Tanzania which estimated the median recovery time of 10 days.^(31)^ The discrepancy could be a result of the time difference between study periods. The difference in the age of the study population could also explain the discrepancy, as the Tanzanian study was conducted among children aged between the ages of 0 and 144 months. The analysis approach (model) used, in the Tanzanian study was logistic regression, which does not take the censoring of study participants into account. The study design in Tanzanian study was a prospective cohort which is better in generating strong evidence.

According to the study conducted in Poland, patients stay for an average of 8.2 to 10.1 days.^(32)^ This variation might be due to high bed occupancy rate in Ethiopia and patients are discharged as soon as their signs and symptoms subside without sending and waiting for any laboratory confirmation. On the contrary, Poland is high-income country compared to Ethiopia, and their health care system might be well organized and developed. The bed occupancy rate in Poland is probably low and people can pay any charge at the hospital that may create an opportunity for patients to stay longer at the hospital until complete stability from all signs and symptoms of the disease.

Duration of time before seeking health care was an independent significant predictor of the recovery time of severe pneumonia. Children who came to the hospital within three days of illness recovered quicker than those children who came after seven days of illness. This finding is supported by a prospective follow-up study conducted in the Gambia ^(12)^ and other retrospective studies conducted in Debre Markos Hospital ^(28)^, Ayder Comprehensive Specialized Hospital ^(22)^, and Central and North Gondar zones.^(33)^ Lately presented cases of pneumonia might develop a complication that needs further investigation to treat and this condition in turn increases the length of time required to recover.^(34)^

The existence of co-morbidity was another significant predictor that was connected with the length of time needed to recover from severe pneumonia in this study. Those children with any type of co-morbidity were found to stay longer in the hospital than their counterparts who were free of comorbidities. This finding is consistent with other studies conducted at Debre Markos ^(28)^, Central and North Gondar zones ^(33)^, and Nepal.^(30)^ The reason why children with comorbidity stay for a prolonged time at the hospital might be due to the fact that comorbidities significantly increase the severity of pneumonia, including the need for oxygen, and are significantly associated with more complications, that increases the recovery time.^(31)^ Since the major comorbidity in the current study was severe acute malnutrition, as adequate protein and energy are needed for proper immune system functioning; undernourished children have a compromised immune system and are more vulnerable to developing serious infections and complications.^(35)^

The antibiotic first administered was one of the significant clinical and treatment-related predictors in this study. Children with severe pneumonia who were treated with ceftriaxone and ampicillin and gentamicin had a prolonged recovery time. This might be due to ampicillin and gentamicin being administered together for patients with bacterial meningitis after testing for cerebrospinal fluid (CSF)^(36)^ and for those children who were presented with severe acute malnutrition in addition to severe pneumonia.^(37)^ Children with severe pneumonia who were treated with ceftazidime and vancomycin as the first antibiotic at the time of admission stayed longer in the hospital. This might be because these drugs are used to treat seriously ill children and infections caused by *staphylococcus aureus* and gram-negative enteric bacilli pneumonia, especially those with penicillin resistance properties. Furthermore, ceftazidime and vancomycin together administered for children with comorbidity, particularly for severe bacterial infections like endocarditis. ^(38)^

Antibiotic change is also a significant predictor of recovery time. Compared to children whose medications were not altered, those whose antibiotics were changed required longer hospital stays to recover from severe pneumonia. Antibiotics are altered whenever there is treatment failure on first-line drugs due to drug resistance or selection of inappropriate drug to treat the patients.^(39)^ For this reason, the additional time required for second-line drugs to act could explain the longer recovery time. This finding is supported by other study conducted in Hosanna.^(8)^

## Limitations of the Study

Although this study uses five years of data, important variables like parent’s income and educational status were not included. The study also didn’t take any laboratory investigation into account like baseline complete blood count, blood culture, and electrolyte sedimentation rate.

# **Conclusion**

This study assessed the recovery time from severe pneumonia and its predictors among children aged 2-59 months admitted to the pediatric ward of Jimma University Medical Center (JUMC). The finding of this study revealed that the median recovery time was longer than other similar studies. Late presentation to the hospital, antibiotic first administered, antibiotic change and the presence of comorbidities were significant predictors that prolonged the recovery time. Healthcare providers should have to give due attention to the identified predictors of recovery time. Measures that help children to recover quickly from their illness should be strengthened.

# **Abbreviations**

**AHR:** Adjusted Hazard Ratio; **BSc:** Bachelor of Science; **CEO:** Chief Executive Officer; **CI:** Confidence Interval; **CHR**: Crude Hazard Ratio; **CSF:** Cerebrospinal Fluid; **FMOHE:** Federal Ministry of Health of Ethiopia; **HIV:** Human Immune Virus; **Hib:** Haemophilus influenzae type b; **ICCM:** Integrated Community Case Management**; IMNCI:** Integrated Management of Newborn and Childhood Illness**; IQR:** Interquartile range**; JUMC:** Jimma University Medical Center**; MRN:** Medical Registration Number**; ORS:** Oral Rehydration Solution**; PCV:** Pneumococcal Conjugate Vaccine**; SAM:** Severe Acute Malnutrition**; SD:** Standard Deviation**; SSA:** Sub-Saharan Africa**; TB:** Tuberculosis**; WHO:** World Health Organization

## Ethical Considerations

Before data collection, this research protocol has been submitted to the Institutional Review Board (IRB) of Jimma University and meets the ethical and scientific standards outlined in national and international guidelines. Jimma University, Institute of Health (Reference No JUIH/IRB/361/23) approved the consent wavier. The letter of permission was written to Jimma University Medical Center managers. Letter to access the patient’s chart was obtained from the chief executive officer (CEO) of Jimma University Medical Center and the health management information system focal person verbally consented to use the patients’ medical chart for data collection. The study was conducted following the relevant international guidelines, regulations, and principles of Helsinki declaration. Verbal or written consent was not obtained from the study participants due to the fact that the data were collected from patients’ medical charts and there was no direct contact between patients and data collectors. Throughout the study, the information obtained from the patient's medical records was kept entirely confidential and only accessed by the research team, and solely for the purpose of the research. For data collection purposes, medical record number were utilized, and no personal identifiers were gathered or utilized in the research report.

# **Acknowledgments**

The authors would like to acknowledge Jimma University Ethical Review Board for providing the ethical clearance letter and Shenen Gibe General Hospital for their valuable and preliminary support. We also extend our heart full gratitude to all the data collectors and supervisors for their efforts in the data collection and chart review process.

# **Funding**

No funds were obtained for this particular study.

# **Disclosure**

No conflict of interest in this particular study.

# **References**

1.	McAllister DA, Liu L, Shi T, Chu Y, Reed C, Burrows J, et al. Global, regional, and national estimates of pneumonia morbidity and mortality in children younger than 5 years between 2000 and 2015: a systematic analysis. Lancet Glob Health. 2019;7(1):e47-e57.

2.	Rudan I, O'Brien KL, Nair H, Liu L, Theodoratou E, Qazi S, et al. Epidemiology and etiology of childhood pneumonia in 2010: estimates of incidence, severe morbidity, mortality, underlying risk factors and causative pathogens for 192 countries. J Glob Health. 2013;3(1):010401.

3.	UNICEF. A fair chance for every child. 2016. 2016.

4.	Pneumonia in children 2019 [Available from: <https://www.who.int/news>.

5.	Naheed A, Breiman RF, Islam MS, Saha SK, Tabassum Naved R. Disparities by sex in care-seeking behaviors and treatment outcomes for pneumonia among children admitted to hospitals in Bangladesh. PLoS One. 2019;14(3):e0213238.

6.	Madsen HO, Hanehøj M, Das AR, Moses PD, Rose W, Puliyel M, et al. Costing of severe pneumonia in hospitalized infants and children aged 2-36 months, at a secondary and tertiary level hospital of a not-for-profit organization. Tropical Medicine & International Health. 2009;14(10):1315-22.

7.	Hauck K, Zhao X. How Dangerous is a Day in Hospital?: A Model of Adverse Events and Length of Stay for Medical Inpatients. Medical Care. 2011;49(12):1068-75.

8.	Tirore LL, Abame DE, Sedoro T, Ermias D, Arega A, Tadesse T, et al. Time to Recovery from Severe Pneumonia and Its Predictors Among Children 2–59 Months of Age Admitted to Pediatric Ward of Nigist Eleni Mohammed Memorial Comprehensive Specialized Hospital, Hossana, Ethiopia: Retrospective Cohort Study. Pediatric Health, Medicine and Therapeutics. 2021;Volume 12:347-57.

9.	Mulholland E, Smith L, Carneiro I, Becher H, Lehmann D. Equity and child-survival strategies. Bull World Health Organ. 2008;86(5):399-407.

10.	Zhang S, Sammon PM, King I, Andrade AL, Toscano CM, Araujo SN, et al. Cost of management of severe pneumonia in young children: systematic analysis. J Glob Health. 2016;6(1):010408.

11.	Marfil-Garza BA, Belaunzaran-Zamudio PF, Gulias-Herrero A, Zuniga AC, Caro-Vega Y, Kershenobich-Stalnikowitz D, et al. Risk factors associated with prolonged hospital length-of-stay: 18-year retrospective study of hospitalizations in a tertiary healthcare center in Mexico. PLoS One. 2018;13(11):e0207203.

12.	Kuti BP, Adegoke SA, Oyelami OA, Ota MO. Predictors of prolonged hospitalisation in childhood pneumonia in a rural health centre. South African Journal of Child Health. 2014;8(1).

13.	Black RE, Morris SS, Bryce J. Where and why are 10 million children dying every year? Lancet. 2003;361(9376):2226-34.

14.	WHO. Revised WHO classification and treatment of childhood pneumonia at health facilities.

15.	Alamneh YM, Adane F. Magnitude and Predictors of Pneumonia among Under-Five Children in Ethiopia: A Systematic Review and Meta-Analysis. J Environ Public Health. 2020;2020:1606783.

16.	Tegenu K, Geleto G, Tilahun D, Bayana E, Bereke B. Severe pneumonia: Treatment outcome and its determinant factors among under-five patients, Jimma, Ethiopia. SAGE Open Med. 2022;10:20503121221078445.

17.	Jimma University Medical Center (JUMC): lucca incrocci foundation; [Available from: <https://www.lucaincroccifoundation.org/JUMC>.

18.	Jimma university medical center health managment information system. 2023.

19.	D D. Sample-Size Formula for the Proportional-Hazards Regression Model. 1983.

20.	Tamirat ZG, Gelagay AA, Boke MM. Time to recovery and its predictors among children	age of 2 up to 59 months admitted with severe community acquired pneumonia in Public Hospitals of Central and North Gondar Zones, Ethiopia 2021. 2022.

21.	Tirore LL, Abame DE, Sedoro T, Ermias D, Arega A, Tadesse T, et al. Time to Recovery	from Severe Pneumonia and Its Predictors Among Children 2-59 Months of Age Admitted to Pediatric Ward of Nigist Eleni Mohammed Memorial Comprehensive Specialized Hospital, Hossana, Ethiopia: Retrospective Cohort Study. Pediatric Health Med Ther. 2021;12:347-57.

22.	Amare RA, Fisseha G, Berhe A, Tirore LL. Incidence of recovery from severe pneumonia and its predictors among children 2-59 months admitted to pediatric ward of Ayder Comprehensive Specialized Hospital, Tigray, Ethiopia: A retrospective Cohort study. J Family Med Prim Care. 2022;11(9):5285-92.

23.	WHO. pneumonia fact sheet. American thoracic society. 2016.

24.	Ndu IK, Ekwochi U, Osuorah CD, Onah KS, Obuoha E, Odetunde OI, et al. Danger Signs of Childhood Pneumonia: Caregiver Awareness and Care Seeking Behavior in a Developing Country. Int J Pediatr. 2015;2015:167261.

25.	Ayalew T. Exclusive breastfeeding practice and associated factors among first-time mothers in Bahir Dar city, North West Ethiopia, removed: A community based cross sectional study. Heliyon. 2020;6(9):e04732.

26.	Tiruneh CM, Emiru TD, Tibebu NS, Abate MW, Nigat AB, Bantie B, et al. Underweight and its associated factors among pediatrics attending HIV Care in South Gondar Zone public health facilities, Northwest Ethiopia, 2021. BMC Pediatr. 2022;22(1):575.

27.	Luzingu JK, Stroupe N, Alaofe H, Jacobs E, Ernst K. Risk factors associated with under-five stunting, wasting, and underweight in four provinces of the Democratic Republic of Congo: analysis of the ASSP project baseline data. BMC Public Health. 2022;22(1):2422.

28.	Mengist B, Tesfa M, Kassie B. Time to recovery and predictors of severe community-acquired pneumonia among pediatric patients in Debre Markos referral hospital, North West Ethiopia: A retrospective follow-up study. PLoS One. 2020;15(9):e0239655.

29.	Harris M, Clark J, Coote N, Fletcher P, Harnden A, McKean M, et al. British Thoracic Society guidelines for the management of community acquired pneumonia in children: update 2011. Thorax. 2011;66 Suppl 2:ii1-23.

30.	Basnet S, Sharma A, Mathisen M, Shrestha PS, Ghimire RK, Shrestha DM, et al. Predictors of duration and treatment failure of severe pneumonia in hospitalized young Nepalese children. PLoS One. 2015;10(3):e0122052.

31.	Caggiano S, Ullmann N, De Vitis E, Trivelli M, Mariani C, Podagrosi M, et al. Factors That Negatively Affect the Prognosis of Pediatric Community-Acquired Pneumonia in District Hospital in Tanzania. Int J Mol Sci. 2017;18(3).

32.	Gajewska M, Lewtak K, Scheres J, Albrecht P, Gorynski P. Trends in Hospitalization of Children with Bacterial Pneumonia in Poland. Cent Eur J Public Health. 2016;24(3):188-92.

33.	Tamirat ZG, Gelagay AA, Boke MM. Time to recovery and its predictors among children age of 2 up to 59 months admitted with severe community acquired pneumonia in Public Hospitals of Central and North Gondar Zones, Ethiopia 2021. 2022.

34.	MacKian S. A review of health seeking behaviour: problems and prospects. 2018.

35.	Kidane GF, Zereabruk K, Aberhe W, Hailay A, Mebrahtom G, Gebremeskel GG, et al. Time to recovery from severe acute malnutrition and its predictors among under five children admitted to therapeutic feeding units of general and referral hospitals in Tigray, Ethiopia, 2020: a prospective cohort study. BMC Pediatr. 2023;23(1):325.

36.	Asghar R, Banajeh S, Egas J, Hibberd P, Iqbal I, Katep-Bwalya M, et al. Chloramphenicol versus ampicillin plus gentamicin for community acquired very severe pneumonia among children aged 2-59 months in low resource settings: multicentre randomised controlled trial (SPEAR study). BMJ. 2008;336(7635):80-4.

37.	UNICEF. Management of Severe Acute Malnutrition in children: working towards results at scale. 2015.

38.	Matsumoto K, Takesue Y, Ohmagari N, Mochizuki T, Mikamo H, Seki M, et al. Practice guidelines for therapeutic drug monitoring of vancomycin: a consensus review of the Japanese Society of Chemotherapy and the Japanese Society of Therapeutic Drug Monitoring. J Infect Chemother. 2013;19(3):365-80.

39.	Muro RP, Masoza TS, Kasanga G, Kayange N, Kidenya BR. Predictors and outcome of first line treatment failure among under-five children with community acquired severe pneumonia at Bugando Medical Centre, Mwanza, Tanzania: A prospective cohort study. PLoS One. 2020;15(12):e0243636.
