# Supplementary material for: Time-to-recovery from severe pneumonia and its predictors among children 2–59 months of age admitted to the pediatric ward of Jimma University Medical Center, Southwest Ethiopia, 2023: A retrospective cohort study
Source: PLoS One. 2025 Apr 15;20(4):e0316839. doi: 10.1371/journal.pone.0316839 (PMC11999157; doi:10.1371/journal.pone.0316839)
Supplement: Attachment — Submitted filename: Response to reviewers.docx [file pone.0316839.s002.docx]

**Manuscript Title**: Time-to-Recovery from Severe Pneumonia and its Predictors	among Children 2-59	Months	of Age Admitted to Pediatric Ward of Jimma University Medical Center, Southwest Ethiopia, 2023: A Retrospective Cohort Study.

**Manuscript ID:** PONE-D-2404566

**Authors:** Getu Girma, Masrie Getnet, Dawit Regassa, Tola Getachew

**Journal:** PLOS ONE

**Article type:** Original Research

**Submitted on:** November 29, 2024, 11:59pm

**Dear Respected Editors/ Reviewers,**

First of all, we would like to appreciate the reviewers for your complimentary comments and suggestions to our manuscript entitled **“Time to Recovery from Severe Pneumonia and its Predictors among Children 2-59 Months of Age Admitted to Pediatric Ward of Jimma University Medical Center, Southwest, Ethiopia, 2023: A Retrospective Cohort Study”**. We highly appreciate the comprehensive feedback throughout the reviewing process and are grateful for the endorsement of this manuscript for publication in your journal **“PLOS ONE”.** In this revised version of the manuscript, we did our best to address all the comments and suggestions raised by the editors and the reviewers. We have submitted a revised copy of the manuscript both in track-changed and without track-changed (clean file) including a response to the reviewer’s file, and supplementary files.

**PLOS ONE| Editorial Office - Collaborative Peer Review Team Comments to Author:**

**Independent Review Report, Reviewer 1**

Abstract: inadequately clear

Response: We appreciate the comments and suggestions; the recommendation you raised were briefly written but we deleted them for the sake of word limit and now we have made corrections in the specified section as per the comments.

Background: needs further enrichment for relevance and contextual factors

Response: we have made corrections in the specified section as per the comments.

Methods: should be presented in explicit and comprehensive manner including ethical considerations.

Response: Thank you for your constructive comments and we made appropriate corrections in the specified section as per the comments

Operational definition: Good if all are referenced with WHO standards or any other = optional

Response: Thank you. We made corrections in the section

Survival time: What is its difference from that of time-to-recovery?

Response: Thank you for the comments, survival time is the general term that indicates the time it takes for the certain event(out come of interest) to occur and our event in this particular study was recovery. Indeed there is no such difference between them and we have deleted survival time

Do you have graph?

Response: Thank you for the comments, yes we do have graphs like Kaplan Meier survival curve and Cox-snell.

Why? What is unique about residence?

Response: Thank you for the comments, in this particular study event(recovery) was higher than censoring but we made a mistake about residence during typing (simply typing error) and so we made corrections in the section.

Results and discussion: lacks brief and comprehensive presentation with appropriate references and justification

Response: Thank you. We made corrections in the section.

Table 2 Revisit your percentages

Response: Thank you. We made corrections in the section.

Vaccination status: Repeated

Response: Thank you. We mistakenly repeated and we deleted repetition.

After performing multivariable Cox regression, adjusted hazard ratios with a 95% confidence interval (CI) and a p-value of less than or equal to 0.05. Thus, variables like the presence of comorbidities, duration of time before seeking health care, antibiotic first administered and antibiotics change were statistically significant predictors of time to recovery from severe pneumonia in this study. Seems complete?

Response: Thank you. We made corrections in the section as After performing multivariable Cox regression, adjusted hazard ratios with a 95% confidence interval (CI) and a p-value of less than or equal to 0.05 were used to declare statistical significance. Thus, Variables such as presence of co-morbidities, duration of time before seeking health care, antibiotic first administered and antibiotics change were statistically significant predictors of time to recovery from severe pneumonia in this study.

Table 3: Age did not contribute. Why?

Response: Thank you. After controlling the potential confounding factors, there is no siginficant difference in the age between 2-11 months and 12-35 months, indicating that the crude effect was likely not due to the factors being studied, but rather the other confounding factors.

In discussion part:

Have you read any single article that tells you this information?

Response: Thank you for the comments and we cited the references as per the comments in our manuscript.

Rewrite. It is a long sentence to capture your idea. So, split into two and rewrite it again

Rewrite in a clear way

Response: Thank you. We made corrections in the section.
